# Integrative taxonomy resolves species identities within the *Macrobiotus pallarii* complex (Eutardigrada: Macrobiotidae)

**DOI:** 10.1186/s40851-021-00176-w

**Published:** 2021-05-27

**Authors:** Daniel Stec, Matteo Vecchi, Magdalena Dudziak, Paul J. Bartels, Sara Calhim, Łukasz Michalczyk

**Affiliations:** 1 Department of Invertebrate Evolution, Institute of Zoology and Biomedical Research, Faculty of Biology, Jagiellonian University, Gronostajowa 9, 30-387 Kraków, Poland; 2 Department of Biological and Environmental Science, University of Jyväskylä, PO Box 35, FI-40014 Jyväskylä, Finland; 3 Department of Biology, Warren Wilson College, Asheville, NC 28815 USA

**Keywords:** cryptic taxa, DNA barcoding, egg ornamentation, new species, species complex, species delimitation

## Abstract

The taxonomy of many groups of meiofauna is challenging due to their low number of diagnostic morphological characters and their small body size. Therefore, with the advent of molecular techniques that provide a new source of traits, many cryptic species have started to be discovered. Tardigrades are not an exception, and many once thought to be cosmopolitan taxa are being found to be complexes of phenotypically similar species. *Macrobiotus pallarii* Maucci, 1954 was originally described in South Italy and has been subsequently recorded in Europe, America, and Asia. This allegedly wide geographic range suggests that multiple species may be hidden under this name. Moreover, recently, genetic evidence to support this was put forward, and the *Macrobiotus pallarii* complex has been proposed to accommodate putative species related to *M. pallarii*. Here, we describe three new pseudocryptic species based on populations that would have been all classified as *Macrobiotus pallarii* if molecular methods were not employed. Using an integrative taxonomy approach, we analyzed animals and eggs from the topotypic population of *Macrobiotus pallarii*, together with four other populations of the complex. We recovered four distinct phylogenetic lineages that, despite the overlap of morphometric traits, can be separated phenotypically by subtle but discrete morphological characters. One lineage corresponds to *Macrobiotus pallarii*, whereas the other three are newly described as *Macrobiotus margoae* Stec, Vecchi & Bartels, **sp. nov**. from the USA, *Macrobiotus ripperi* Stec, Vecchi & Michalczyk, **sp. nov**. from Poland and Finland, and *Macrobiotus pseudopallarii* Stec, Vecchi & Michalczyk, **sp. nov**. from Montenegro. To facilitate species identification, we provide a dichotomous key for species of the *M. pallarii* complex. Delimitation of these pseudocryptic taxa highlights the need for an integrative approach to uncover the phylum’s diversity in full.

**Supplementary Information:**

The online version contains supplementary material available at 10.1186/s40851-021-00176-w.

## Background

Tardigrades constitute a phylum of microinvertebrates inhabiting both terrestrial and aquatic (freshwater and marine) habitats worldwide [1], with approximately 1300 species known so far [2–4]. Tardigrade taxonomy is challenging due to their small size, limited number of taxonomically informative traits, and many outdated species descriptions that do not comply with modern taxonomic standards. Advances have been made possible with the recent advent of integrative taxonomy (*e.g.,* [5–10]) that includes the use of DNA sequencing with detailed morphological techniques such as scanning electron microscopy (SEM) and sophisticated analysis of morphometric data. Many studies using an integrative taxonomy approach have recently revealed that various tardigrade species once thought to be widespread are actually complexes of cryptic species with more localized distributions [5, 6, 11, 12]. In addition to their distribution, tardigrade cryptic species have also been shown to diverge based on reproductive mode [13], ploidy [5], and anhydrobiotic survival [14]. Three classes have been recognized in the phylum Tardigrada: Heterotardigrada, Mesotardigrada, and Eutardigrada [15]. The class Heterotardigrada encompasses both primarily marine tardigrades (Arthrotardigrada) and mostly limnoterrestrial armoured tardigrades (Echiniscoidea). The class Mesotardigrada has been found only once from a thermal spring in Japan and is considered a *classis dubium* [16]. The class Eutardigrada comprises two mostly limnoterrestrial orders, Apochela and Parachela. The order Parachela, which is the most common, widespread, and speciose group with a wide range of dietary preferences [17], comprises the superfamily Macrobiotoidea. The nominal genus for the superfamily *Macrobiotus* CAS Schultze, 1834 [18] is characterized by the presence of symmetrical diploclaws, a rigid buccal tube with a straight ventral bar lacking a ventral hook, two macroplacoids and one microplacoid in the pharynx, 10 peribuccal lamellae, pores in the cuticle, and freely laid ornamented eggs [19]. *Macrobiotus* is one of the most species-rich and widespread genera in the phylum, and it was also the first formally described tardigrade genus [20]. Animals of the *Macrobiotus pallarii* complex have the very typical morphology of *Macrobiotus*. However, this group is characterized by egg ornamentation composed of large conical processes separated by a single row of areolation (such eggs are also known in other macrobiotid genera, such as *Paramacrobiotus* Guidetti et al., 2009 [21] but have not been found in other *Macrobiotus* species). To date, three described species belong to this complex, with the type species being *Macrobiotus pallarii* Maucci, 1954 [22], described from South Italy and a senior synonym of *Macrobiotus aviglianae* Robotti, 1970 [23, 24], which was described from North Italy. *Macrobiotus pallarii* has been found in various localities in Europe, Asia and North America [25]; however, none of the records outside the type locality have been genetically verified; thus, the true geographic range of this species is unknown. The second species, *Macrobiotus ragonesei* Binda et al., 2001 [26]*,* was described and found only in Central Africa [26, 27], whereas the third species, *Macrobiotus caymanensis* Meyer, 2011 [28], was found only in the Cayman Islands (the Caribbean). Additionally, three phylogenetically distinct but undescribed and morphologically very similar species of this group have been recently detected in several localities in Europe and North America [19]. Given that the existence of these species and morphological characters that allow for their phenotypic identification became apparent only after the molecular analysis had been performed, they can be considered pseudocryptic species [29, 30]. To elucidate the taxonomy of this species complex, we analyzed (and resequenced) the populations reported by Stec et al. [19], as well as individuals and eggs from the type locality of *M. pallarii*, from phylogenetic (multilocus phylogeny and species delimitation), morphometric (principal component analysis; PCA), and morphological perspectives. Morphometric traits alone do not allow for a separation of *M. pallarii* and the three new pseudocryptic species, as almost all their biometric values overlap considerably. However, our analysis identified some qualitative phenotypic characters that could be used to separate and formally describe three new species within this morphologically uniform species complex. Last, to facilitate species identification, we provide a dichotomous key for species of the *M. pallarii* complex.

## Materials and methods

### Samples and specimens

Five populations of the *Macrobiotus pallarii* complex were isolated from moss samples collected from five localities in Europe and North America (see Table 1 for details). The samples were examined for tardigrades using the protocol by [31], with modifications described in detail in [32]. The live animals and eggs were placed into culture. Specimens were reared in plastic Petri dishes according to the protocol by [32]. To perform the taxonomic analysis of these species/populations, animals and eggs were taken from the cultures and split into several groups for specific analyses: (i) morphological and morphometric analyses with light contrast microscopy (LCM), (ii) morphological analysis with SEM, and (iii) DNA sequencing (for details see sections “Material examined” provided below for each description).

**Table 1:** Information on moss samples with the populations of the *Macrobiotus pallarii* complex analyzed in the present study. *Type locality of *Macrobiotus pallarii*

Sample/population code	Locality	Coordinates and altitude	Collectors
FI.066	Finland, Jyväskylä, Graniitti	62°13′24.6″N 25°46′20.4″E 84 m asl	Matteo Vecchi
ME.007	Montenegro, Crkvine	42°47′57.54″N 19°27′18.47″E 1015 m asl	Aleksandra Rysiewska
PL.015	Poland, Malinówka, Yew Reserve	49°42′09″N 21°55′53″E 382 m asl	Piotr Gąsiorek
US.057	USA, Great Smoky Mountains National Park, Purchase Knob	35°35′7.84″N 83°4′26.47″W 1492 m asl	Nate Gross & Mackenzie McClay
IT.337*	Italy, Cosenza, Silvana Mansio	39°18′34.5″N 16°32′19.9″E 1436 m asl	Francesco Squillace

### Comparative materials

Measurements of the type series of *M. caymanensis* from the Cayman Islands (KY) and additional information on morphological traits were kindly provided by Harry Meyer (McNeese State University, USA). Photomicrographs of the animals and eggs from the type series of *M. pallarii* from the Maucci collection (Civic Museum of Natural History of Verona, Italy) were taken by MV and Roberto Guidetti (University of Modena and Reggio Emilia, Italy). Moreover, several eggs of a *Macrobiotus polyopus* group species collected in Papua New Guinea (ca. 8°20′S, 146°16′E, 10 m asl) in 2007 by Anna Millard (University of East Anglia, UK) were compared with the *Macrobiotus pallarii* complex populations analyzed herein.

### Genotyping

DNA was extracted from individual animals following a *Chelex® 100* resin (*BioRad*) extraction method by [33] with modifications described in detail in [34]. Individuals from the same populations that were sequenced by Stec *et al*. [19] (populations FI.066-PL.015-ME.007-US.057) were resequenced in the present study, as [19] provided sequences at the haplotype level, whereas species delimitation methods require sequences at the level of individuals. Each specimen was mounted in water and examined under LCM prior to DNA extraction. We sequenced four DNA fragments, three nuclear fragments (18S rRNA, 28S rRNA, ITS-2) and one mitochondrial fragment (COI). All fragments were amplified and sequenced according to the protocols described in [34]; primers with original references are listed in Table 2. Sequencing products were read with an ABI 3130xl sequencer at the Molecular Ecology Lab, Institute of Environmental Sciences of Jagiellonian University, Kraków, Poland. Sequences were processed in BioEdit ver. 7.2.5 [42] and submitted to NCBI GenBank [43]. For accession numbers see Table 3.

**Table 2:** Primers with their original references used for amplification of the four DNA fragments sequenced in the study. The primer set LCO1490-JJ + HCO2198-JJ was used for COI amplification in four populations (FI. 066, IT. 337, PL. 015, US. 057), whereas LCO1490 + HCOoutout was used for one population (ME.007)

DNA marker	Primer name and source	Primer direction	Primer sequence (5’-3’)
**18S rRNA**	18S_Tar_Ff1 [35]	forward	AGGCGAAACCGCGAATGGCTC
	18S_Tar_Rr1 [35]	reverse	GCCGCAGGCTCCACTCCTGG
**28S rRNA**	28S_Eutar_F [36]	forward	ACCCGCTGAACTTAAGCATAT
	28SR0990 [37]	reverse	CCTTGGTCCGTGTTTCAAGAC
**ITS-2**	ITS2_Eutar_Ff [38]	forward	CGTAACGTGAATTGCAGGAC
	ITS2_Eutar_Rr [38]	reverse	TCCTCCGCTTATTGATATGC
**COI**	LCO1490-JJ [39]	forward	CHACWAAYCATAAAGATATYGG
	HCO2198-JJ [39]	reverse	AWACTTCVGGRTGVCCAAARAATCA
	LCO1490 [40]	forward	GGTCAACAAATCATAAAGATATTGG
	HCOoutout [41]	reverse	GTAAATATATGRTGDGCTC

**Table 3:** GenBank accession numbers of sequences used in the present study. Newly generated sequences are in bold

Taxon	Individual	18S	28S	COI	ITS2
*Macrobiotus caelestis*		MK737073	MK737071	MK737922	MK737072
*Macrobiotus pallarii* complex	FI.066.1	**MT809075**	**MT809088**	**MT807929**	
	FI.066.2	**MT809076**	**MT809089**	**MT807930**	**MT809103**
	FI.066.3			**MT807931**	**MT809104**
	FI.066.4			**MT807932**	**MT809105**
	IT.337.1	**MT809069**	**MT809081**	**MT807924**	**MT809094**
	IT.337.2	**MT809070**	**MT809082**	**MT807925**	**MT809095**
	IT.337.3	**MT809071**	**MT809083**	**MT807926**	**MT809096**
	ME.007.1	**MT809065**	**MT809077**		**MT809090**
	ME.007.2	**MT809066**	**MT809078**	**MT807920**	
	ME.007.3	**MT809067**	**MT809079**	**MT807921**	**MT809091**
	ME.007.4	**MT809068**	**MT809080**	**MT807922**	**MT809092**
	ME.007.5			**MT807923**	**MT809093**
	PL.015.1	**MT809074**	**MT809086**		**MT809100**
	PL.015.2		**MT809087**	**MT807933**	**MT809101**
	PL.015.3			**MT807934**	
	PL.015.4			**MT807935**	**MT809102**
	US.057.1	**MT809072**	**MT809084**	**MT807927**	**MT809098**
	US.057.2	**MT809073**	**MT809085**		
	US.057.3			**MT807928**	**MT809099**
	US.057.4				**MT809097**

### Phylogenetic analysis

To reconstruct the phylogeny, we used sequences representing four markers (18S rRNA, 28S rRNA, ITS-2, and COI) from five different populations of the *Macrobiotus pallarii* complex. Sequences were downloaded from GenBank or produced *de novo* (Table 3). Type sequences of *Macrobiotus caelestis* Coughlan, Michalczyk & Stec, 2019 [44] were used as the outgroup. 18S rRNA, 28S rRNA, and ITS-2 sequences were aligned with MAFFT ver. 7 [45, 46] with the G-INS-i method (thread=4, threadtb=5, threadit=0, reorder, adjustdirection, anysymbol, maxiterate=1000, retree 1, globalpair input). COI sequences were aligned according to their amino acid sequences (translated with the invertebrate mitochondrial code) with the MUSCLE algorithm [47] in MEGA ver. 7.0.26 [48] with default settings (all gap penalties=0, max iterations=8, clustering method=UPGMB, lambda=24) and then translated back to nucleotide sequences. Alignments were visually inspected and trimmed in MEGA ver. 7.0.26 [48]. The absence of saturation in the COI and ITS-2 alignments was confirmed with transition/transversion and saturation plots (SM.0^1^) produced with the R package “ape ver. 5.0” [49]. Aligned sequences were concatenated with an in-house R script (written by MV, available upon request to the author). Model selection and phylogenetic reconstructions were performed on the CIPRES Science Gateway [50]. Model selection was performed for each alignment partition (6 in total: 18S, 28S, ITS-2, and three COI codons) with PartitionFinder ver. 2 [51]. Bayesian inference (BI) phylogenetic reconstruction was performed with MrBayes ver. 3.2.6 [52] without BEAGLE. Four runs with one cold chain and three heated chains were run for 20 million generations with a 10% burn-in, sampling a tree every 10000 generations. Posterior distribution was checked with Tracer ver. 1.7 [53]. The MrBayes input file is available as Supplementary Material (SM.0^2^). The phylogenetic tree was visualized with FigTree ver. 1.4.4 [54], and the image was edited with Inkscape ver. 0.92.3 [55].

### Species delimitation

Following the suggestion of [56], species delimitation was performed with both a tree-based (bPTP) and a distance-based (ABGD) method. Tree-based species delimitation was performed with bPTP software [57] on both ITS-2 and COI trees. Single gene alignments and BI trees were produced as described above (see Phylogenetic Analysis section). One thousand trees were sampled randomly from the posterior tree distribution after discarding the burn in (250 from each chain) and used as input for bPTP with 100,000 generations, a thinning of 100 generations and 10% burn in. Distance-based species delineation was performed with the ABGD online server [58] on both ITS-2 and COI alignments obtained for the phylogenetic analysis as described above. For both markers, simple distance was used, with 10 steps, a relative gap width of 1.5, and 20 bins for distance distribution. For COI, Pmin and Pmax were 0.01 and 0.1, respectively, while for ITS-2, they were 0.0001 and 0.1, respectively, to accommodate the difference in divergence between these loci.

### The p-distances

As the species of the *M. pallarii* complex are phylogenetically and morphologically distinct [19], the p-distances for the genetic differential diagnosis were calculated between species of the *M. pallarii* complex for the four sequenced markers (18S, 28S, ITS2, and COI) using the alignment used for the phylogenetic analysis. Pairwise distances were calculated with the software MEGA ver. 7.0.26 [48] using pairwise deletion for the Gap/Missing Data Treatment option. Detailed p-distance tables are provided in SM.0^3^.

### Microscopy and imaging

Specimens for LCM were mounted on microscope slides in a small drop of Hoyer’s medium and secured with a cover slip, following the protocol by [59]. Slides were examined under an Olympus BX53 light microscope with phase (PCM) and Nomarski differential interference contrast (NCM) associated with an Olympus DP74 digital camera. To obtain clean and extended specimens for SEM, tardigrades were processed according to the protocol by [32]. Specimens were examined under high vacuum in a Versa 3D DualBeam Scanning Electron Microscope (SEM) at the ATOMIN facility of Jagiellonian University, Kraków, Poland. All figures were assembled in Corel Photo-Paint X6, ver. 16.4.1.1281. For structures that could not be satisfactorily focused in a single LCM photograph (PCM and NCM), a stack of 2–6 images was taken with an equidistance of ca. 0.2 μm and assembled manually into a single deep-focus image in Corel Photo-Paint X6, ver. 16.4.1.1281.

### Morphometrics and morphological nomenclature

All measurements are given in micrometers (μm). Sample size was adjusted following recommendations by [60]. Structures were measured only if their orientation was suitable. Body length was measured from the anterior extremity to the end of the body, excluding the hind legs. The terminology used to describe oral cavity armature and eggshell morphology follows [61, 62]. Macroplacoid length sequence is given according to [63]. Buccal tube length and the level of the stylet support insertion point were measured according to [64]. The *pt* index is the ratio of the length of a given structure to the length of the buccal tube expressed as a ratio [64]. Measurements of buccal tube widths, heights of claws and eggs follow [62]. Morphometric data were handled using the “Parachela” ver. 1.7 template available from the Tardigrada Register [65]. Tardigrade taxonomy followed [19, 66].

Morphometric data for eggs and animals were analyzed with PCA. All analyses were performed with the base R software package [67]. For eggs, absolute values (raw measurements in μm) were used for the analysis, whereas for the animals, relative (*pt*) values were analyzed. Missing data in the animal data set were imputed with the PCA imputation technique with the “imputePCA” function of the R package “missMDA ver. 1.17” [68]. The number of components used to impute the missing values was determined by cross-validation (function “estim_ncpPCA”). PCAs were performed on the scaled data with the PCA function of the package “FactoMineR ver. 2.3” [69]. PCAs were visualized with the packages “ggplot2 ver. 3.3.2”, “plyr ver. 1.8.6” and “gridExtra ver. 2.3” [70, 71]. The presence of a structure in the PCA data was tested with a randomization procedure according to [72] on the eigenvalues and with the statistics ψ and ϕ using an in-house R script (written by MV, in Supplementary SM.04a-4b). PERMANOVA was performed on the PCs using the species hypothesis obtained by phylogenetic methods as the independent variable with the R package “vegan ver. 2.5.6” and “pairwiseAdonis ver. 0.3” [73]. The R script of all the morphometric analyses is available as Supplementary SM.4a-4b. Additionally, morphometric data from the type series of *Macrobiotus caymanensis* were included in this analysis (population name: Cayman). The use of Thorpe normalization proposed by [74] for the comparison of morphometric traits between different species was not used due to the low sample size for *M. pallarii* and *M. caymanensis*. The α-level for multiple post hoc comparisons was adjusted separately for adult and egg traits using the Benjamini-Hochberg correction [75].

## Availability of data and materials

The datasets supporting the conclusions of this article are available in the GenBank repository (https://www.ncbi.nlm.nih.gov/genbank; Accession Numbers in Table 3) and in the Tardigrada Register: under www.tardigrada.net/register/0103.htm (*M. pallarii*), www.tardigrada.net/register/0104.htm (*M. pseudopallarii* **sp. nov.**), www.tardigrada.net/register/0105.htm (*M. ripperi* **sp. nov.**) and www.tardigrada.net/register/0106.htm (*M. margoae* **sp. nov.**). New species nomenclatural acts were registered in ZooBank (http://zoobank.org, see Taxonomic Account for URLs). Raw morphometric measurements of the analyzed populations supporting the conclusions of this article are included within the article (Additional files: SM.0^5^, SM.0^6^, SM.0^7^, SM.0^8^, SM.0^9^).

## Results

### Phylogenetic analysis

**Fig. 1:**
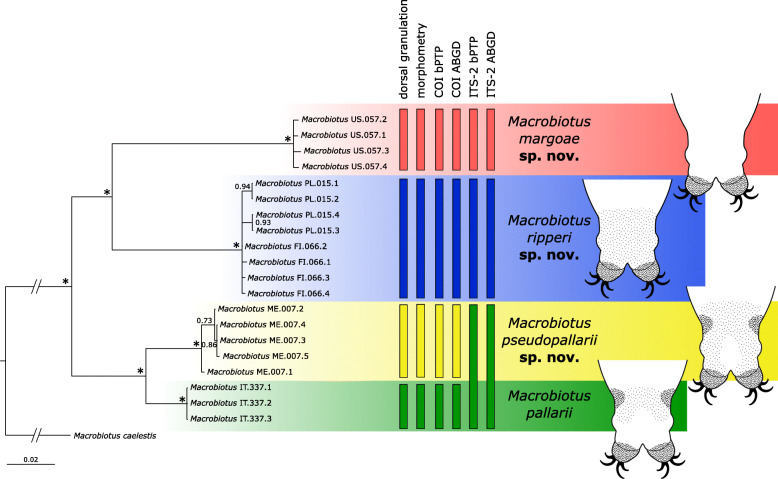
BI Phylogenetic reconstruction of the relationships between the analyzed specimens from the 5 populations. Nodes with posterior probability (pp) <0.70 were collapsed; asterisks indicate nodes with pp=1.00. Vertical bars show the results of different species delimitation methods or discriminant characters. Horizontal colored boxes highlight the identified species described in the Taxonomic Account. A schematic representation of the dorso-caudal granulation patterns for each described species is presented (for a detailed description, see the Taxonomic Account)

The BI phylogenetic reconstructions yielded a topology (Fig. 1) with four well-supported clades: the first clade comprised all individuals from the US population; the second clade contained all individuals from the Polish (PL) and Finnish (FI) populations; the third clade contained individuals from the Montenegrin (ME) population; and the fourth clade contained individuals from the IT population.

### Species delimitation

The bPTP analysis of COI and ITS-2 markers gave slightly different results (Fig. 1). The species identified based on the COI dataset are in agreement with the four clades found with the phylogenetic analysis. The ITS-2 dataset species delimitation, however, found only three species, as the IT and ME populations were delineated as a single species. The ABGD results were congruent with the bPTP results (Fig. 1). For COI, only for the smallest prior intraspecific divergence (0.01) were six species recovered (the PL and FI populations were delimited as two species); however, we did not consider this result to be valid, as it was not congruent with all the other nine evaluated prior intraspecific divergence values or with the bPTP result for the same marker.

### Morphometric analysis

**Fig. 2:**
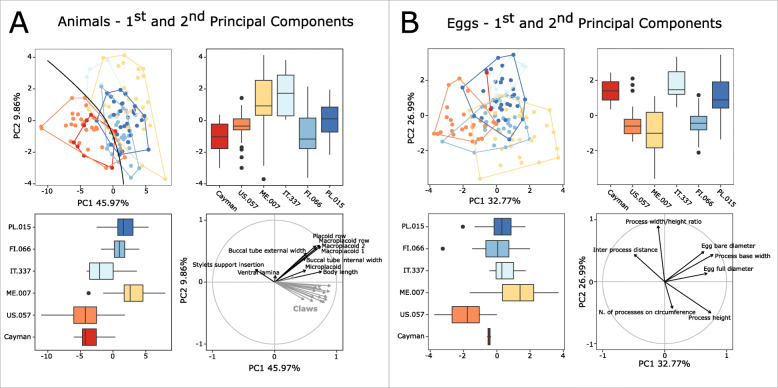
Results of PCA of animal *pt* indices and egg raw measurements. **a** Animal *pt* indices, 1^st^ and 2^nd^ Principal Components; **b** Egg measurements, 1^st^ and 2^nd^ Principal Components; Top-left quadrants: score scatterplots; Top-right and bottom-left quadrants: boxplots of single component scores; bottom-right: loading plot

The randomization test in the PCA revealed that for both animal and egg datasets, only the first two PCs explained more variation than expected by the null model (no data structure) (Supplementary SM.10); therefore, only the first two PCs were retained and used for further analysis and interpretation. Additionally, the ψ and ϕ statistics of the PCA were significantly different from their expectations under the null model (animals: ψ=132.75 p<0.001, ϕ=0.45 p<0.001; eggs: ψ=6.29 p<0.001, ϕ=0.39 p<0.001). Principal component analysis (PCA) of the *pt* indices of animals (Fig. 2a) described 56% of the total variance with the first two components (46.0% for PC1 and 9.9% for PC2). PERMANOVA showed an overall significant effect of species identity on the PCs (p<0.001, Table 4). The majority of the *post hoc* pairwise PERMANOVA comparisons were significant (Table 4); however, the species could not be separated by any of the analyzed traits (Fig. 2a), which was also indicated by low R^2^ values (Table 4), thus making morphometric indices impractical for traditional species identification. The only exception was represented by two groups of populations (*M. margoae* + *M. caymanensis vs M. pallarii* + *M. pseudopallarii* + *M. ripperi*) that showed some separation in the first and second PCs (Fig. 2a). According to the loading plot of PC1 and PC2 (Fig. 2a), the separation between these two groups was driven mainly by the *pt* indices related to the buccal apparatus structures. Principal component analysis (PCA) of egg measurements (Fig. 2b) described 60% of the total variance with the first two components (32.8% for PC1 and 27.0% for PC2). PERMANOVA showed an overall significant effect of the species on the PCs (p<0.001, Table 5). All of the *post hoc* pairwise PERMANOVA comparisons, except some concerning *M. caymanensis* (probably due to the very low sample size of this species), were significant (Table 5). However, similar to animal traits, egg measurements also did not separate the analyzed species (Fig. 2b).

**Table 4:** Results of PERMANOVA and *post hoc* pairwise PERMANOVA comparisons for the first two principal components (PCs) of animal *pt* values; significant *post hoc* p-values at the α-level of p<0.045 (i.e., adjusted with the Benjamini-Hochberg correction) are in bold

Term			df	SS	F	R^**2**^	p
Species			4	1066.73	38.49	0.54	**<0.001**
Residuals			131	907.59		0.46	
Total			135	1974.32		1.00	
***Post hoc*** **comparisons**							
*M. pallarii*	*vs*	*M. ripperi* **sp. nov.**	1	83.70	16.85	0.20	**<0.001**
*M. ripperi* **sp. nov.**	*vs*	*M. pseudopallarii* **sp. nov.**	1	89.40	15.00	0.14	**<0.001**
*M. ripperi* **sp. nov.**	*vs*	*M. margoae* **sp. nov.**	1	625.77	107.08	0.54	**<0.001**
*M. ripperi* **sp. nov.**	*vs*	*M. caymanensis*	1	166.50	36.56	0.35	**<0.001**
*M. pseudopallarii* **sp. nov.**	*vs*	*M. margoae* **sp. nov.**	1	754.91	82.24	0.58	**<0.001**
*M. pseudopallarii* **sp. nov.**	*vs*	*M. caymanensis*	1	270.68	30.58	0.45	**<0.001**
*M. pallarii*	*vs*	*M. pseudopallarii* **sp. nov.**	1	101.20	10.53	0.22	**<0.001**
*M. pallarii*	*vs*	*M. margoae* **sp. nov.**	1	83.99	9.00	0.20	**0.002**
*M. pallarii*	*vs*	*M. caymanensis*	1	51.05	5.85	0.29	**0.007**
*M. margoae* **sp. nov.**	*vs*	*M. caymanensis*	1	6.84	0.79	0.02	0.396

**Table 5:** Results of PERMANOVA and *post hoc* pairwise PERMANOVA comparisons for the first two principal components (PCs) of egg measurements; significant *post hoc* p-values at the α-level of p<0.040 (i.e., adjusted with the Benjamini-Hochberg correction) are in bold

Term			df	SS	F	R^**2**^	p
Species			4	272.16	25.097	0.44	**<0.001**
Residuals			125	338.88		0.55	
Total			129	611.03		1.00	
***Post hoc*** **comparisons**							
*M. pallarii*	*vs*	*M. pseudopallarii* **sp. nov.**	1	59.32	18.54	0.32	**<0.001**
*M. pallarii*	*vs*	*M. margoae* **sp. nov.**	1	91.88	43.88	0.54	**<0.001**
*M. ripperi* **sp. nov.**	*vs*	*M. pseudopallarii* **sp. nov.**	1	61.02	20.61	0.18	**<0.001**
*M. ripperi* **sp. nov.**	*vs*	*M. margoae* **sp. nov.**	1	119.54	47.97	0.35	**<0.001**
*M. pallarii*	*vs*	*M. ripperi* **sp. nov.**	1	18.26	7.42	0.09	**0.002**
*M. caymanensis*	*vs*	*M. pseudopallarii* **sp. nov.**	1	22.38	6.08	0.16	**0.007**
*M. caymanensis*	*vs*	*M. margoae* **sp. nov.**	1	11.20	4.88	0.14	**0.015**
*M. caymanensis*	*vs*	*M. pallarii*	1	3.74	2.16	0.17	0.115
*M. caymanensis*	*vs*	*M. ripperi* **sp. nov.**	1	5.65	2.17	0.03	0.114
*M. pseudopallarii* **sp. nov.**	*vs*	*M. margoae* **sp. nov.**	1	3.74	2.16	0.17	**<0.001**

## Taxonomic accounts

**Phylum:** Tardigrada Doyère, 1840 [76]

**Class:** Eutardigrada Richters, 1926 [77]

**Order:** Parachela Schuster et al., 1980 [78] (restored by [15])

**Superfamily:** Macrobiotoidea Thulin, 1928 [79] (in [80])

**Family:** Macrobiotidae Thulin, 1928 [79]

**Genus:** *Macrobiotus* C.A.S. Schultze, 1834 [18]

***Macrobiotus pallarii*** **Maucci, 1954** [22]

**Material examined:** 17 animals and 15 eggs. Specimens were mounted on microscope slides in Hoyer’s medium (9 animals + 10 eggs), fixed on SEM stubs (5+5), and processed for DNA sequencing (3+0).

**Locality:** 39°18′34.5″N, 16°32′19.9″E; 1436 m asl: Silvana Mansio, Cosenza, Italy: moss on rock in sparse forest; coll. 16 December 2019 by Francesco Squillace.

**Specimen depositories:** Fourteen animals (slides: IT.337.03–11; SEM stub: 19.19) and 15 eggs (slides: IT.337.01–02; SEM stub: 19.19) were deposited at the Institute of Zoology and Biomedical Research, Jagiellonian University, Gronostajowa 9, 30-387, Kraków, Poland.

## Integrative description of the topotypic population of the species

**Fig. 3:**
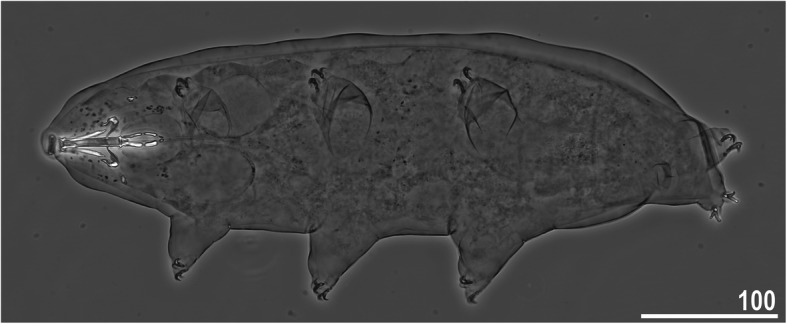
*Macrobiotus pallarii* Maucci, 1954 from the topotypic population (IT.337) – habitus, adult specimen in dorso-ventral projection. Scale bar in μm

**Fig. 4:**
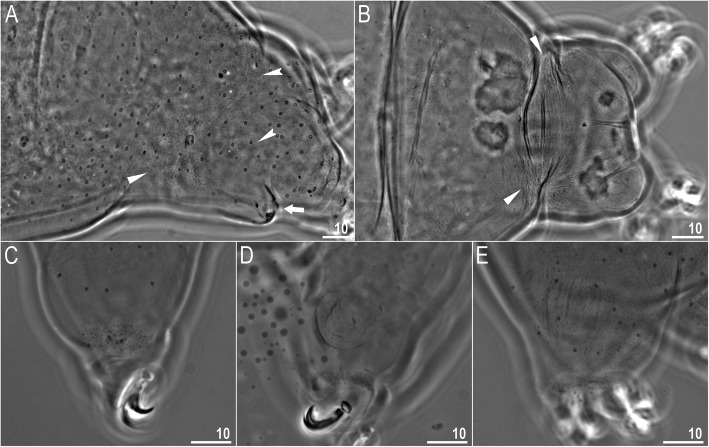
*Macrobiotus pallarii* Maucci, 1954 from the topotypic population (IT.337) – body and leg cuticle morphology seen with LCM: **a–b** – band of caudal granulation on the last body segment clearly visible in specimen with stretched cuticle (A) and hardly visible in specimen with wrinkled cuticle (**b**); **c** – granulation on the external surface of leg III; **d** – internal surface of leg III with evident pulvinus; **e** – granulation on dorsal surface of leg IV. Filled flat arrowheads indicate dense patches of granulation in the caudal band, filled indented arrowheads indicate sparse granulation in the caudal band, arrow indicates lateral gibbosity on the IV leg – a male secondary sexual character. Scale bar in μm

**Fig. 5:**
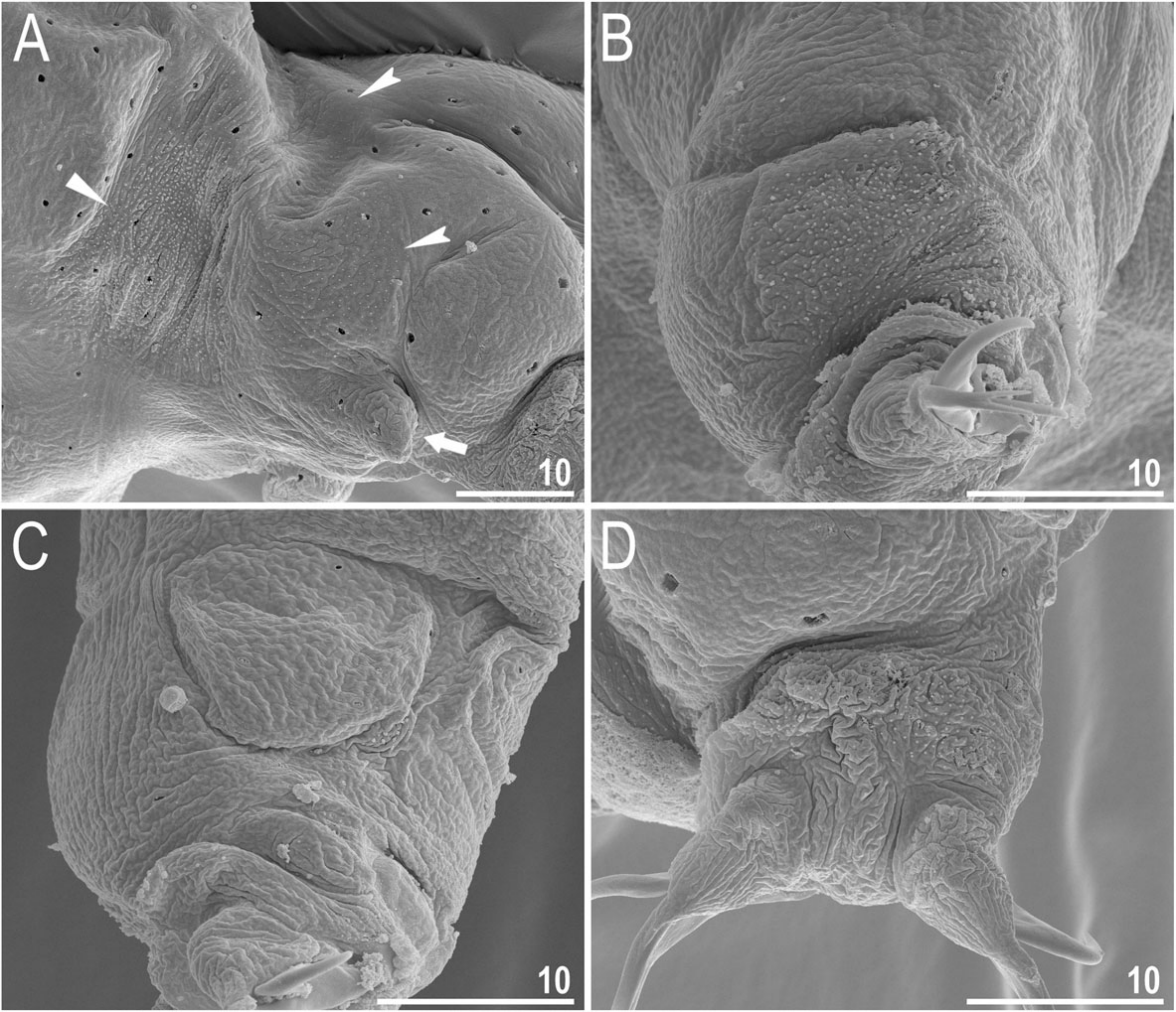
*Macrobiotus pallarii* Maucci, 1954 from the topotypic population (IT.337) – body and leg cuticle morphology seen with SEM: **a** – band of caudal granulation on the last body segment; **b** – granulation on the external surface of leg II; **c** – internal surface of leg II with evident pulvinus; **d** – granulation on dorsal surface of leg IV. Filled flat arrowheads indicate dense patches of granulation in the caudal band, filled indented arrowheads indicate sparse granulation in the caudal band, arrow indicates lateral gibbosity – a male secondary sexual character. Scale bar in μm

***Animals*** *(measurements and statistics in* Table 6*):* In live animals, body almost transparent in smaller specimens and whitish in larger animals; transparent after fixation in Hoyer’s medium (Fig. 3). Eyes present in live animals and after fixation in Hoyer’s medium. Small round and oval cuticular pores (0.5–1.5 μm in diameter) visible under both LCM and SEM scattered randomly throughout the entire body (Figs. 4a–e, 5a–d). Patches of fine granulation on the external surface of legs I–III as well as on the dorsal and dorsolateral sides of leg IV visible in LCM (Fig. 4c, e) and SEM (Fig. 5b, d). A pulvinus is present on the internal surface of legs I–III (Figs. 4d, 5c). In addition to the typical patches of leg granulation, a band of granulation is present on the dorso- and latero-caudal surface of the last body segment (Figs. 2, 4a, 5a). It consists of two lateral patches of dense granulation joined by a band of sparse dorsal granulation (Figs. 2, 4a, 5a). The sparse granulation also extends posteriorly from these dense granulation patches towards the granulation on leg IV, but they never connect (Figs. 2, 4a, 5a). This granulation can be poorly visible under LCM when the cuticle is wrinkled (Fig. 4b).

**Table 6:** Measurements [in μm] of selected morphological structures of individuals of *Macrobiotus pallarii* Maucci, 1954 from the topotypic population (IT.337) mounted in Hoyer’s medium (N–number of specimens/structures measured, RANGE refers to the smallest and the largest structures among all measured specimens; SD–standard deviation)

CHARACTER	N	RANGE		MEAN		SD	
		μm	***pt***	μm	***pt***	μm	***pt***
Body length	9	421 – 617	*1005 – 1413*	536	*1169*	77	*136*
Buccal tube							
Buccal tube length	8	41.7 – 48.1	–	44.9	*–*	2.6	*–*
Stylet support insertion point	8	32.5 – 37.3	*77.1 – 79.4*	35.0	*77.9*	1.9	*0.8*
Buccal tube external width	8	5.9 – 7.2	*13.9 – 16.6*	6.8	*15.2*	0.5	*1.0*
Buccal tube internal width	8	3.8 – 5.1	*9.1 – 11.8*	4.7	*10.4*	0.4	*0.9*
Ventral lamina length	8	22.6 – 28.2	*51.4 – 59.4*	25.3	*56.4*	1.8	*2.8*
Placoid lengths							
Macroplacoid 1	8	12.6 – 16.1	*28.5 – 34.6*	14.3	*31.9*	1.3	*2.1*
Macroplacoid 2	8	7.9 – 10.5	*18.5 – 21.9*	9.0	*20.1*	0.8	*1.1*
Microplacoid	8	3.3 – 5.1	*7.5 – 11.8*	4.2	*9.3*	0.6	*1.4*
Macroplacoid row	8	21.8 – 27.0	*52.0 – 60.8*	25.0	*55.7*	1.8	*2.8*
Placoid row	8	26.6 – 32.8	*61.3 – 75.6*	30.2	*67.4*	2.1	*4.2*
Claw 1 heights							
External primary branch	8	9.9 – 12.7	*23.6 – 27.7*	11.3	*24.9*	1.1	*1.5*
External secondary branch	7	7.9 – 10.0	*18.2 – 23.1*	8.6	*19.3*	0.8	*1.7*
Internal primary branch	8	8.4 – 12.7	*20.1 – 25.4*	10.5	*23.0*	1.4	*1.8*
Internal secondary branch	6	7.1 – 8.6	*16.6 – 19.9*	7.7	*17.6*	0.6	*1.2*
Claw 2 heights							
External primary branch	8	10.1 – 13.8	*23.5 – 30.3*	11.5	*25.2*	1.4	*2.3*
External secondary branch	8	8.4 – 10.3	*18.1 – 23.8*	9.1	*20.3*	0.8	*1.8*
Internal primary branch	8	9.3 – 12.4	*21.7 – 27.7*	10.7	*23.4*	1.3	*2.2*
Internal secondary branch	8	7.4 – 9.8	*17.0 – 22.6*	8.3	*18.6*	0.8	*1.9*
Claw 3 heights							
External primary branch	8	9.9 – 13.5	*22.5 – 30.9*	11.7	*25.8*	1.5	*2.8*
External secondary branch	8	7.7 – 10.4	*17.7 – 23.3*	9.1	*20.1*	1.1	*2.0*
Internal primary branch	8	8.7 – 12.6	*20.9 – 29.1*	10.9	*24.1*	1.5	*2.7*
Internal secondary branch	8	7.5 – 9.8	*17.3 – 22.6*	8.3	*18.6*	0.9	*1.9*
Claw 4 heights							
Anterior primary branch	7	11.7 – 14.3	*26.6 – 30.6*	13.0	*28.6*	0.9	*1.7*
Anterior secondary branch	6	8.8 – 10.2	*19.3 – 22.2*	9.5	*21.1*	0.5	*1.1*
Posterior primary branch	6	12.5 – 16.2	*29.5 – 34.6*	14.3	*31.5*	1.4	*2.0*
Posterior secondary branch	3	9.9 – 10.5	*22.1 – 24.2*	10.3	*23.0*	0.3	*1.1*

**Fig. 6:**
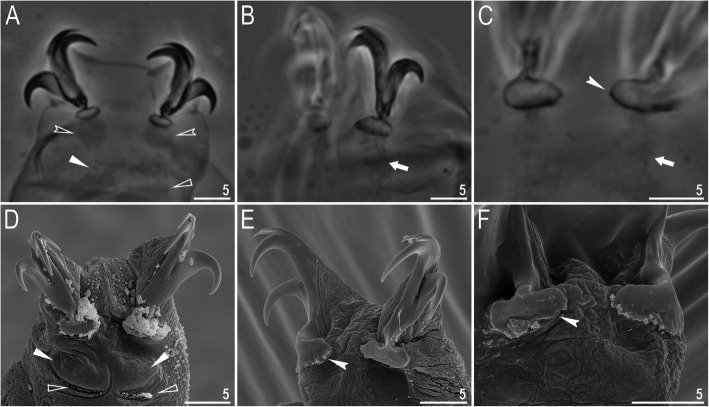
*Macrobiotus pallarii* Maucci, 1954 from the topotypic population (IT.337) – claw morphology: **a–b** – claws I and IV seen with LCM; **c** – magnification on lunulae IV seen with LCM; **d–e** – claws II and IV seen in SEM; **f** – magnification on lunulae IV seen with SEM. Empty indented arrowheads indicate dark circular areas under lunulae on the first three pairs of legs, filled flat arrowheads indicate cuticular bars above muscle attachments, empty flat arrowheads indicate double muscle attachments under claws, filled indented arrowheads indicate faintly visible indentations on lunulae IV, arrows indicate horseshoe structures connecting the anterior and posterior claws. Scale bars in μm

Claws slender, of the *hufelandi* type. Primary branches with distinct accessory points, a long common tract, and an evident stalk connecting the claw to the lunula (Fig. 6a–f). Lunulae on all legs smooth and only sometimes faintly crenulated on leg IV (Fig. 6a–f). Dark areas under each claw on legs I–III were often visible in LCM (Fig. 6a). Paired muscle attachments and faintly visible cuticular bars above them on legs I–III were often visible both with LCM and SEM (Fig. 6a, d), whereas the horseshoe-shaped structure connecting anterior and posterior claw IV was visible only in LCM (Fig. 6b–c).

**Fig. 7:**
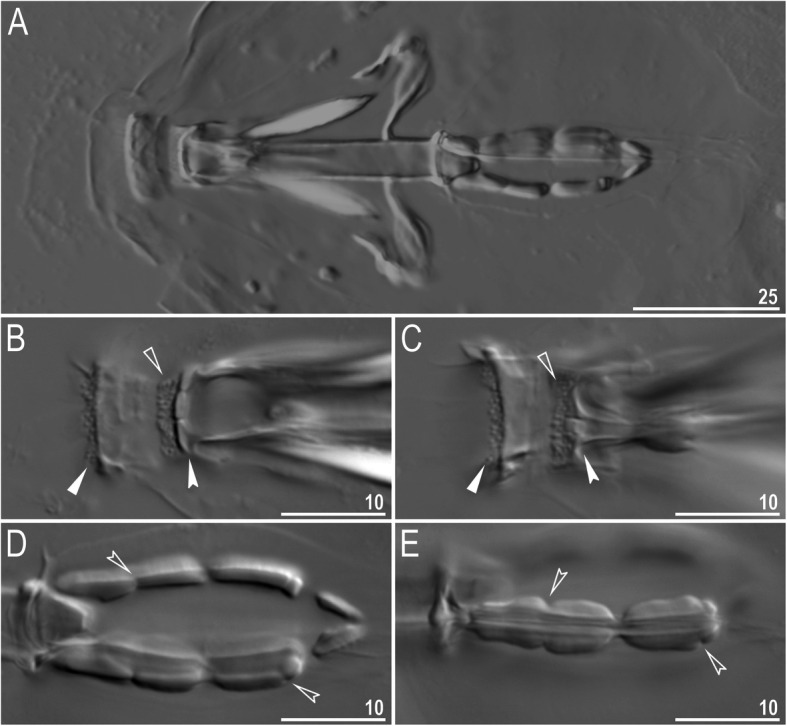
*Macrobiotus pallarii* Maucci, 1954 from the topotypic population (IT.337) – buccal apparatus seen with LCM: **a** – an entire buccal apparatus; **b–c** – the oral cavity armature, dorsal and ventral teeth, respectively; **d–e** – placoid morphology, dorsal and ventral placoids, respectively. Filled flat arrowheads indicate the first band of teeth, empty flat arrowheads indicate the second band of teeth, filled indented arrowheads indicate the third band of teeth and empty indented arrowheads indicate central and subterminal constrictions in the first and second macroplacoid. Scale bars in μm

**Fig. 8:**
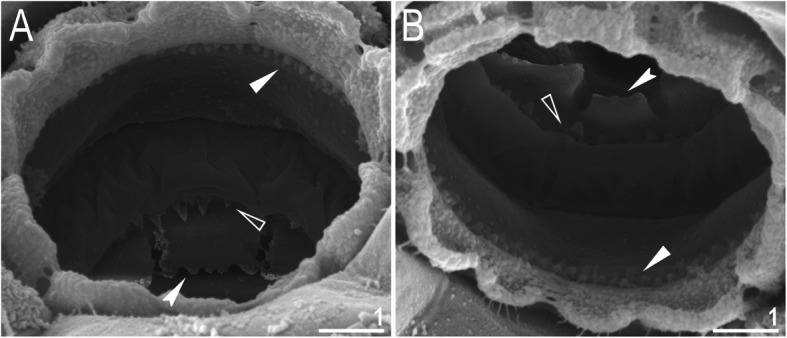
*Macrobiotus pallarii* Maucci, 1954 from the topotypic population (IT.337) – the oral cavity armature seen with SEM: **a–b** – the oral cavity armature of a single specimen seen with SEM from different angles showing dorsal and ventral portion, respectively. Filled flat arrowheads indicate the first band of teeth, empty flat arrowheads indicate the second band of teeth, and filled indented arrowheads indicate the third band of teeth. Scale bars in μm

Mouth antero-ventral. Buccal apparatus of the *Macrobiotus* type (Fig. 7a), with the ventral lamina and ten peribuccal lamellae (Fig. 8a–b). The oral cavity armature was well developed and composed of three bands of teeth, all always clearly visible under LCM (Fig. 7b–c). The first band of teeth is composed of numerous small teeth visible under LCM as granules (Fig. 7b–c) and under SEM as cones (Fig. 8a–b), arranged in several rows, situated anteriorly in the oral cavity, just behind the bases of the peribuccal lamellae. The second band of teeth is situated between the ring fold and the third band of teeth and comprises 3–4 rows of teeth visible with LCM as granules (Fig. 7b–c), and as cones in SEM (Fig. 8a–b) but larger than those in the first band. The most anterior row of teeth within the second band comprises larger teeth than the subsequent posterior rows (Fig. 7b–c). The teeth of the third band are located within the posterior portion of the oral cavity, between the second band of teeth and the buccal tube opening (Figs. 7b–c, 8a–b). The third band of teeth is divided into the dorsal and ventral portions. Under both LCM and SEM, the dorsal teeth are seen as three distinct transverse ridges, whereas the ventral teeth appear as two separate lateral transverse ridges, between which one large tooth (sometimes circular in LCM) is visible (Figs. 7b–c, 8a–b). In SEM, teeth of the third band have faintly indented margins (Fig. 8a–b). Pharyngeal bulb spherical, with triangular apophyses, two rod-shaped macroplacoids (2<1) and a microplacoid positioned close to them (i.e., the distance between the second macroplacoid and the microplacoid is shorter than the microplacoid length; Fig. 7a, d). The first macroplacoid is anteriorly narrowed and constricted in the middle, whereas the second has a subterminal constriction (Fig. 7d–e).

**Fig. 9:**
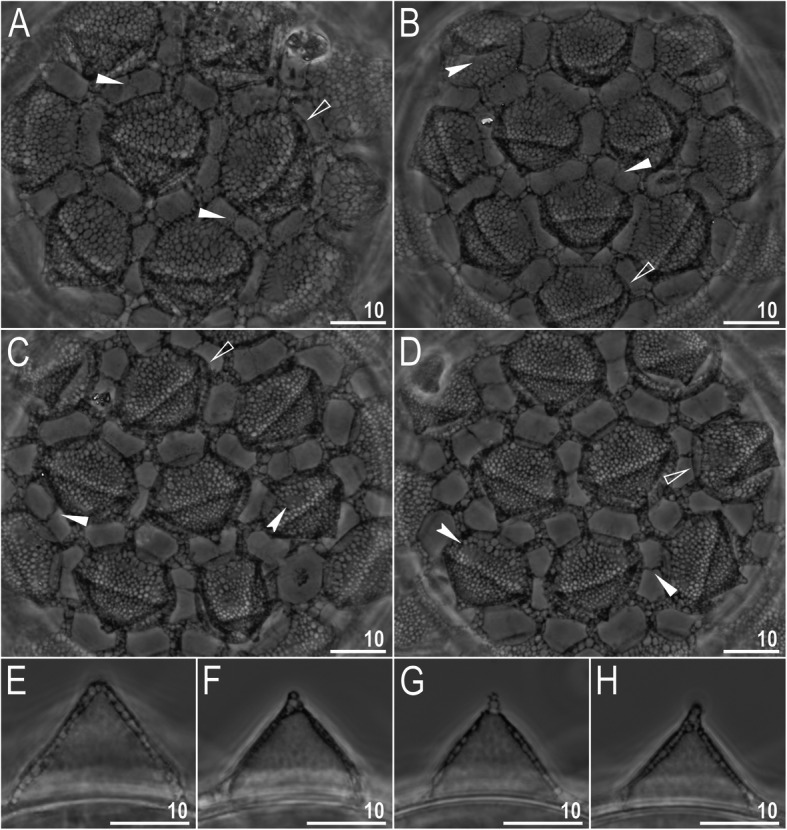
*Macrobiotus pallarii* Maucci, 1954 from the topotypic population (IT.337) – eggs seen with LCM: **a–d** – surface under ×1000 magnification of four different eggs; **e–h** – midsections of four different egg processes. Filled flat arrowheads indicate thickening perpendicular to the process base that divides the areola in the middle, filled indented arrowheads indicate areas of the egg processes without a reticulation/labyrinthine layer, and empty flat arrowheads indicate irregular collar around process bases. Scale bars in μm

**Fig. 10:**
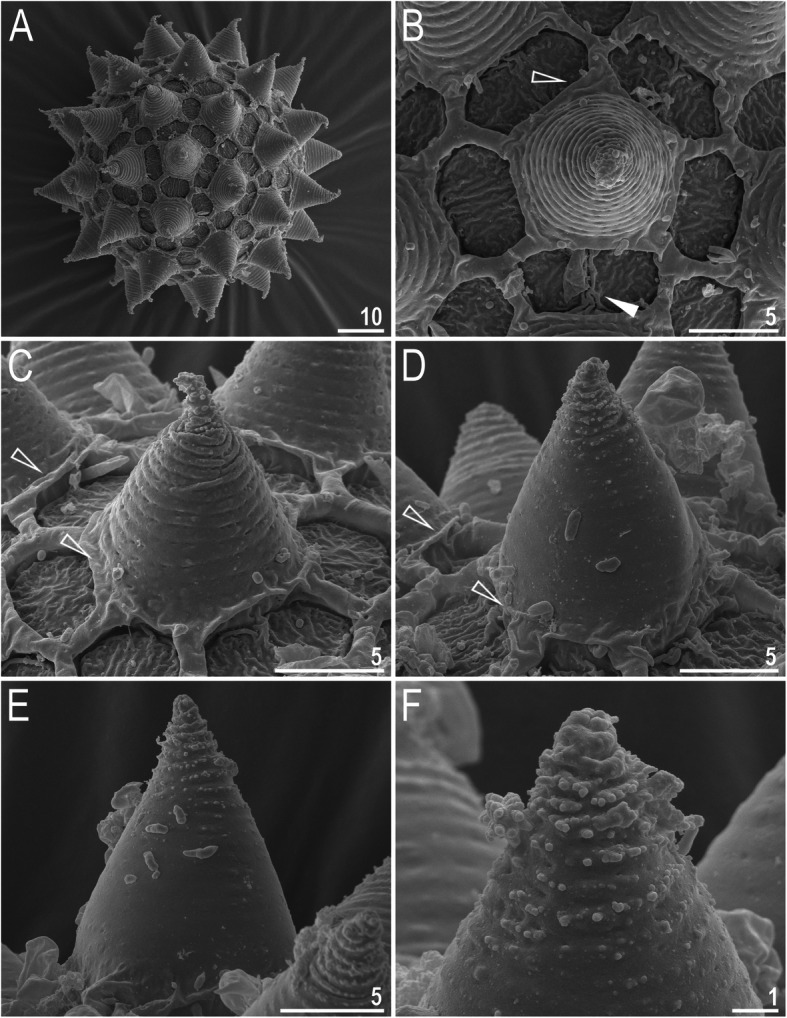
*Macrobiotus pallarii* Maucci, 1954 from the topotypic population (IT.337) – eggs seen with SEM: **a** – entire view of the egg; **b–f** – details of the egg surface between processes, areolation and egg processes. Filled flat arrowheads indicate thickening perpendicular to the process base that divides the areola in the middle, empty flat arrowheads indicate irregular collar around process bases. Scale bars in μm

***Eggs*** *(measurements and statistics in* Table 7*):* Laid freely, white, spherical with conical processes surrounded by one row of areolae (Figs. 9, 10a–f). In SEM, multiple rings of tight annulation were visible on the entire process (Fig. 10a–c), although in some processes, annulation was present only in the upper portion of the process (Fig. 10d–f) (annulation not visible in LCM because it was obscured by the eminent labyrinthine layer). The upper parts of the processes are covered by granulation visible only under SEM, which to a varying extent is distributed on annuli (Fig. 10c–f). The labyrinthine layer between the process walls is present and visible as reticulation with circular meshes throughout the entire process (Fig. 9a–d). Small areas without reticulation are rarely present in some processes (Fig. 9b–d). The upper part of the process is often elongated into short flexible apices (Figs. 9f–h, 10a–c), which are occasionally absent or broken (Figs. 9e, 10d–f). The base of the processes extends into the six (only sometimes five) arms that form areolae rims (Figs. 9a–d, 10a–c). Each process is surrounded by six (only sometimes five) hexagonal areolae (Figs. 9a–d, 10a–c), which are occasionally falsely subdivided in the middle into two areolae by a thin thickening perpendicular to the process base (Figs. 9a–d, 10b). Areolae rims (walls) thick and usually flat (Fig. 10a–d), with the labyrinthine layer inside the rims visible as bubbles in LCM (Fig. 9a–d). Areolae rims also delimit the areolae at the bases of processes, which forms an irregular collar around process bases (Figs. 9a–d, 10a–d) and makes the process bases penta- or hexagonal in the top view (Figs. 9a–d, 10a–b). The areola surface has wrinkles that are faintly visible under LCM (Fig. 9a–d) but clearly visible under SEM (Fig. 10a–d). Micropores are present within the areolae, but they are distributed only around the areolae rims and are usually absent in the central part of the areola (Fig. 10b–d).

**Table 7:** Measurements [in μm] of selected morphological structures of the eggs of *Macrobiotus pallarii* Maucci, 1954 from the topotypic population (IT.337) mounted in Hoyer’s medium (N–number of eggs/structures measured, RANGE refers to the smallest and the largest structures among all measured specimens; SD–standard deviation)

CHARACTER	N	RANGE	MEAN	SD
Egg bare diameter	10	71.3 – 81.1	76.1	4.2
Egg full diameter	10	97.6 – 108.0	102.4	4.3
Process height	30	12.2 – 15.2	13.5	0.8
Process base width	30	14.1 – 21.0	16.4	1.7
Process base/height ratio	30	103% – 146%	121%	12%
Interprocess distance	30	4.5 – 8.1	6.1	0.7
Number of processes on the egg circumference	10	10 – 12	11.2	0.8

### Reproduction

**Fig. 11:**
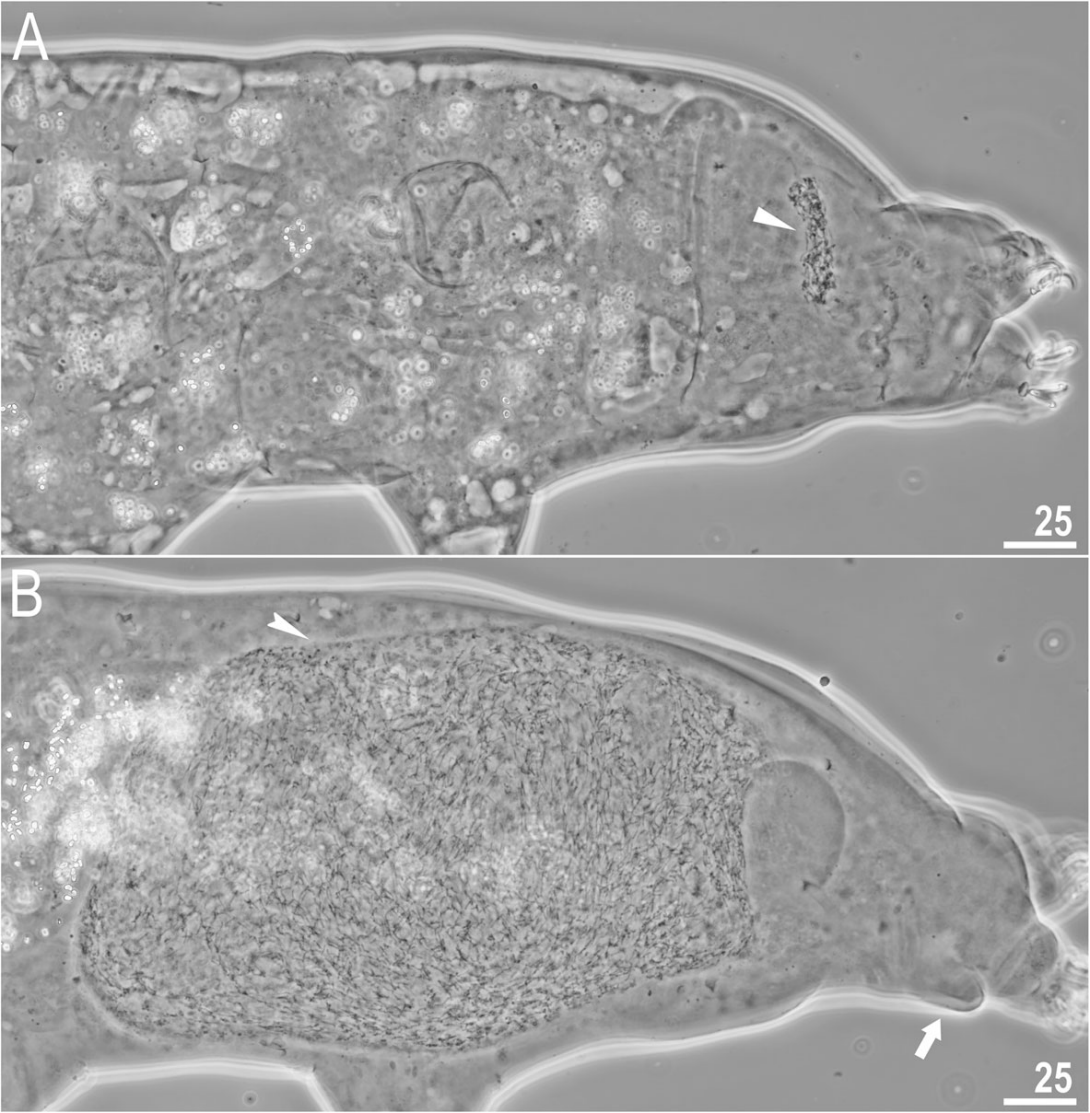
*Macrobiotus pallarii* Maucci, 1954 from the topotypic population (IT.337) – reproduction (LCM): **a** – spermatheca (seminal vesicle) filled with spermatozoa and visible in females freshly mounted in Hoyer’s medium; **b** – testis filled with sperm visible in a male freshly mounted in Hoyer’s medium. The flat arrowhead indicates the female spermathecae, the indented arrowhead indicates the testis, and the arrow indicates the gibbosity on the IV leg. Scale bars in μm

The species is dioecious. Spermathecae in females as well as testes in males have been found to be filled with spermatozoa, clearly visible under PCM up to 24 hours after mounting in Hoyer’s medium (Fig. 11a–b). The species exhibits secondary sexual dimorphism in the form of clearly visible lateral gibbosities on hind legs in males (Figs. 4a, 5a, 11b).

### DNA sequences and intraspecific genetic distances

- **18S rRNA**: GenBank: MT809069–71; 987 bp long; 1 haplotype was found.
- **28S rRNA**: GenBank: MT809081–3; 716 bp long; 1 haplotype was found.
- **ITS-2**: GenBank: MT809094–6; 362 bp long; 1 haplotype was found.
- **COI**: GenBank: MT807924–6; 630 bp long. 1 haplotype was found.

### Phenotypic differential diagnosis

By having the processes surrounded by 5–6 areolae, it resembles four other species of the *Macrobiotus pallarii* complex out of which three are newly described in this study. By the morphology of the animals and eggs, this species can be differentiated specifically from the following:

- ***Macrobiotus pseudopallarii*** **sp. nov.**: by faintly crenulated lunulae IV (lunulae are gently dentate in *M. pseudopallarii* **sp. nov.**) and the sparse granulation on the dorso-caudal end of the body connecting the dense granulation patches between legs III and IV not extending posteriorly to the granulation on leg IV (with sparse granulation extending posteriorly to the granulation on leg IV in *M. pseudopallarii* **sp. nov.**; see Fig. 1).
- ***Macrobiotus ripperi*** **sp. nov.**: by faintly crenulated lunulae IV (lunulae are dentate in *M. ripperi* **sp. nov.**), the presence of two lateral patches of dense granulation between legs III and IV (patches of dense granulation are absent in *M. ripperi* **sp. nov.**; see Fig. 1), the sparse granulation on the dorso-caudal end of the body connecting the dense granulation patches between legs III and IV not extending posteriorly to the granulation on legs IV (with the dense granulation patches between legs III and IV absent and the sparse granulation extending posteriorly to the granulation on legs IV in *M. ripperi* **sp. nov.**; see Fig. 1) and by the presence of granulation on the tips of egg processes (granulation is absent in *M. ripperi* **sp. nov.**; character visible only under SEM).
- ***Macrobiotus margoae*** **sp. nov.**: by faintly crenulated lunulae IV (lunulae are dentate in *M. margoae* **sp. nov.**), the presence of two lateral patches of dense granulation between legs III and IV (the lateral patches of dense granulation are absent in *M. margoae* **sp. nov.**; see Fig 1), the presence of sparse dorsal granulation between legs III and IV (this granulation absent in *M. margoae* **sp. nov.**; see Fig. 1), all three bands of teeth in the oral cavity visible under LCM (the first band of teeth is not visible under LCM in *M. margoae* **sp. nov.**), a higher placoid row *pt* value (*61.3–75.6* in *M. pallarii* vs. *51.1–60.6* in *M. margoae* **sp. nov.**), the labyrinthine meshes within the entire process wall (only small circular bubbles scattered randomly within the process wall are found in *M. margoae* **sp. nov.**), and by the presence of granulation on the tips of egg processes (granulation is absent in *M. margoae* **sp. nov.**; character visible only under SEM).
- ***Macrobiotus caymanensis***, known only from the Cayman Islands: by all three bands of teeth in the oral cavity visible with LCM (the first band of teeth not visible with LCM in *M. caymanensis*), the presence of granulation visible with LCM on all legs (leg granulation is absent or not visible under LCM in *M. caymanensis*), a higher placoid row *pt* value (*61.3–75.6* in *M. pallarii* vs. *47.8–59.9* in *M. caymanensis*), and by the labyrinthine meshes within the entire process wall (only small circular bubbles scattered randomly within the process wall are found in *M. caymanensis*).

### Genotypic differential diagnosis

Interspecific genetic p-distances between *M. pallarii* and other species of the *M. pallarii* complex are as follows:

- **18S rRNA**: 0.0–1.2% (0.7% on average), with the most similar being *Macrobiotus pseudopallarii* **sp. nov.** from Montenegro (MT809065–7), and the least similar being *Macrobiotus margoae* **sp. nov.** from the USA (MT809072–3).
- **28S rRNA**: 0.1–2.7% (1.8% on average), with the most similar being *Macrobiotus pseudopallarii* **sp. nov.** from Montenegro (MT809077–80), and the least similar being *Macrobiotus margoae* **sp. nov.** from the USA (MT809084–5).
- **ITS-2**: 0.8–6.7% (4.1% on average), with the most similar being *Macrobiotus pseudopallarii* **sp. nov.** Haplotype 1 (H1) from Montenegro (MT809090–2), and the least similar being *Macrobiotus margoae* H2 **sp. nov.** from the USA (MT809097).
- **COI**: 14.0–21.1% (17.5% on average), with the most similar being *Macrobiotus pseudopallarii* **sp. nov.** Haplotype 2 (H2) from Montenegro (MT807920), and the least similar being *Macrobiotus margoae* **sp. nov.** from USA (MT807927–8).

***Macrobiotus pseudopallarii*** **Stec, Vecchi & Michalczyk, sp. nov.**

*Macrobiotus* cf. *pallarii* ME.007 [19]

**Zoobank:** urn:lsid:zoobank.org:act:2F0C8594-A645-4146-86A2-99C7BF9C307C

**Etymology:** The name refers to the morphology of the new species, which highly resembles that of *Macrobiotus pallarii* (Latin “*pseudo*” = “false”).

**Material examined:** 96 animals and 130 eggs. Specimens were mounted on microscope slides in Hoyer’s medium (76 animals + 116 eggs), fixed on SEM stubs (15+14), and processed for DNA sequencing (5+0).

**Type locality:** 42°47′57.54″N, 19°27′18.47″E; 1015 m asl: Montenegro: Crkvine; moss on stone; coll. May 2018 by Aleksandra Rysiewska.

**Type depositories:** Holotype (slide ME.007.05 with 9 paratypes), 81 paratypes (slides: ME.007.06–10; SEM stub: 18.09) and 130 eggs (slides: ME.007.01–04; SEM stub: 18.09) were deposited at the Institute of Zoology and Biomedical Research, Jagiellonian University, Gronostajowa 9, 30-387, Kraków, Poland.

## Description of the new species

**Fig. 12:**
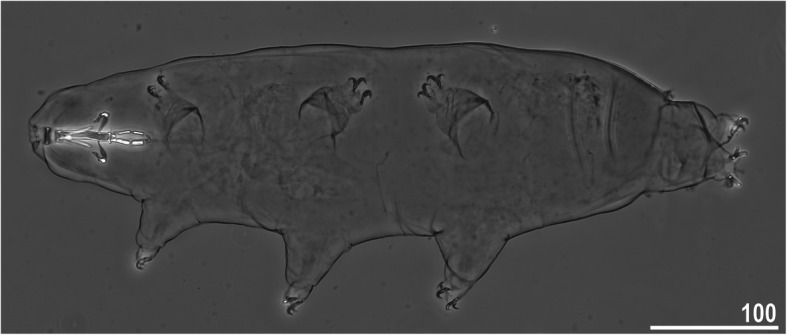
*Macrobiotus pseudopallarii* **sp. nov.** from Montenegro (ME.007) – habitus, adult specimen in dorso-ventral projection (holotype). Scale bar in μm

**Fig. 13:**
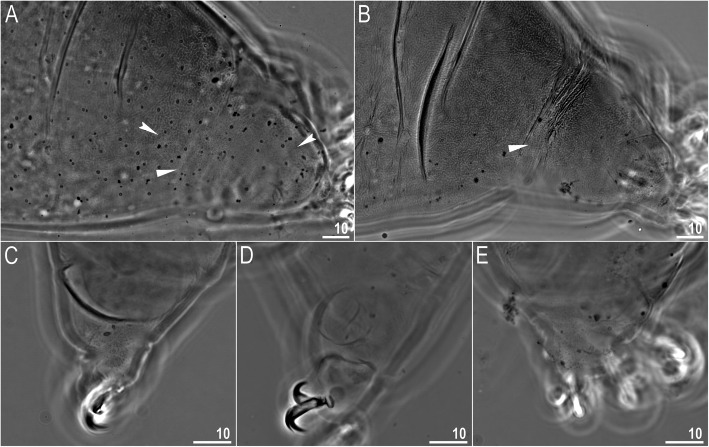
*Macrobiotus pseudopallarii* **sp. nov.** from Montenegro (ME.007) – body and leg cuticle morphology seen with LCM: **a–b** – band of caudal granulation on the last body segment clearly visible in specimen with stretched cuticle (**a**) and hardly visible in specimen with wrinkled cuticle (**b**); **c** – granulation on the external surface of leg III; **d** – internal surface of leg III with evident pulvinus; **e** – granulation on dorsal surface of leg IV. Filled flat arrowheads indicate dense patches of granulation in the caudal band, filled indented arrowheads indicate sparse granulation in the caudal band. Scale bar in μm

**Fig. 14:**
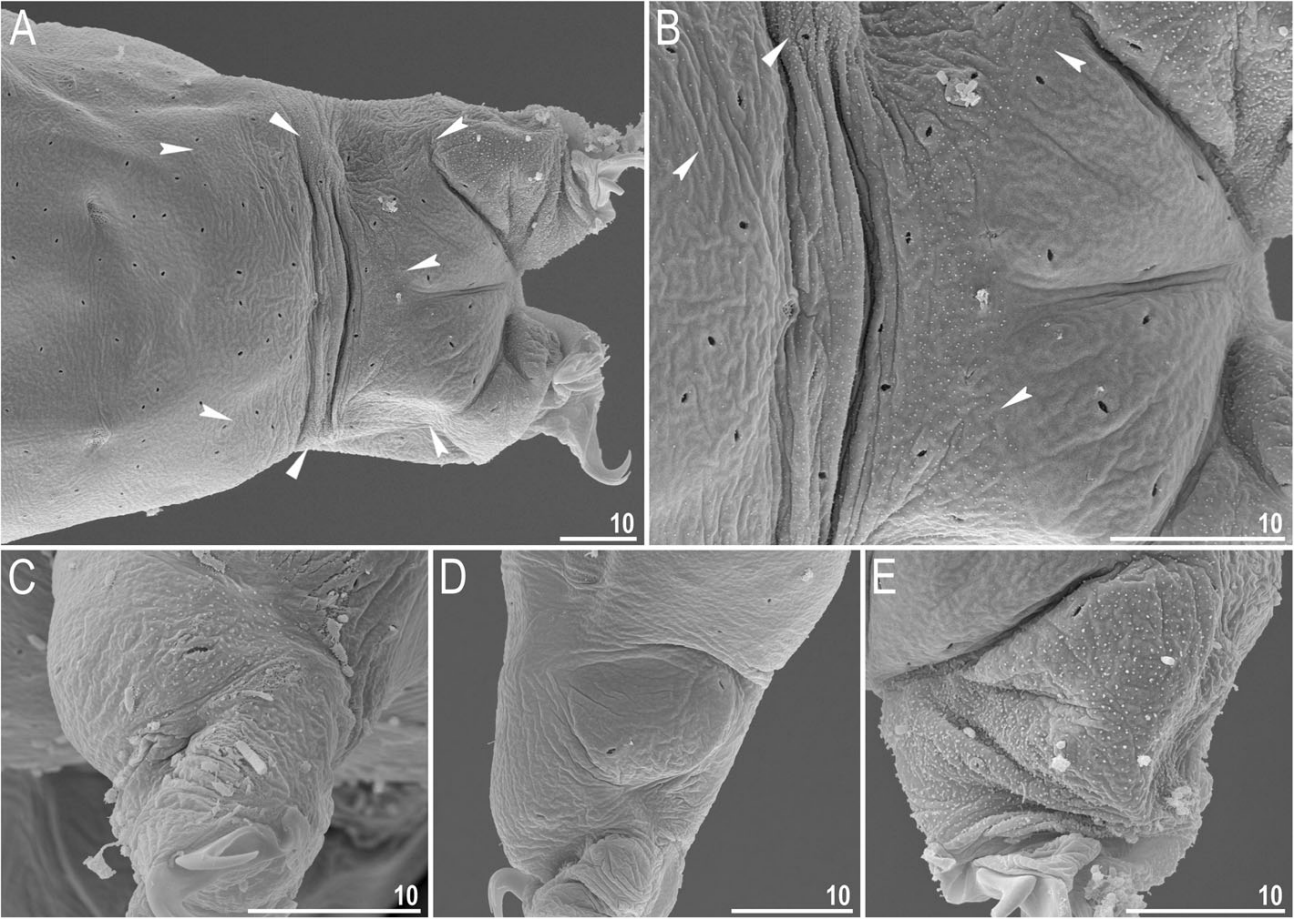
*Macrobiotus pseudopallarii* **sp. nov.** from Montenegro (ME.007) – body and leg cuticle morphology seen with SEM: **a–b** – band of caudal granulation on the last body segment; **c** – granulation on the external surface of leg II; **d** – internal surface of leg II with evident pulvinus; **e** – granulation on dorsal surface of leg IV. Filled flat arrowheads indicate dense patches of granulation in the caudal band, filled indented arrowheads indicate sparse granulation in the caudal band. Scale bar in μm

***Animals*** *(measurements and statistics in* Table 8*):* In live animals, body almost transparent in smaller specimens and whitish in larger animals; transparent after fixation in Hoyer’s medium (Fig. 12). Eyes present in live animals and after fixation in Hoyer’s medium. Small round and oval cuticular pores (0.5–1.2 μm in diameter), visible under both LCM and SEM, scattered randomly throughout the entire body (Figs. 13a–e, 14a–e). Patches of fine granulation on the external surface of legs I–III as well as on the dorsal and dorsolateral sides of leg IV visible with LCM (Fig. 13c, e) and SEM (Fig. 14c, e). A pulvinus is present on the internal surface of legs I–III (Figs. 13d, 14d). In addition to the typical patches of leg granulation, a band of granulation is present on the dorso- and latero-caudal surface of the last body segment (Figs. 2, 13a, 14a–b). It consists of two lateral patches of dense granulation, joined with each other by a band of sparse dorsal granulation (Figs. 2, 13a, 14a–b). The sparse granulation also extends anteriorly and posteriorly from those dense granulation patches with posterior extension, which connects with the granulation on leg IV (Figs. 2, 13a, 14a–b). This granulation is slightly visible under LCM when the cuticle is wrinkled (Fig. 2, 13b).

**Table 8:** Measurements [in μm] of selected morphological structures of individuals of *Macrobiotus pseudopallarii* **sp. nov.** from Montenegro (ME.007) mounted in Hoyer’s medium (N–number of specimens/structures measured, RANGE refers to the smallest and the largest structures among all measured specimens; SD–standard deviation)

CHARACTER	N	RANGE		MEAN		SD		Holotype	
		μm	***pt***	μm	***pt***	μm	***pt***	μm	***pt***
Body length	30	386 – 580	*957 – 1328*	507	*1215*	45	*78*	534	*1275*
Buccal tube									
Buccal tube length	30	36.3 – 47.8	–	41.8	*–*	3.0	*–*	41.9	*–*
Stylet support insertion point	30	27.9 – 37.9	*76.1 – 79.8*	32.5	*77.8*	2.5	*1.1*	32.3	*77.1*
Buccal tube external width	30	5.2 – 8.5	*13.3 – 19.4*	6.8	*16.2*	0.8	*1.4*	6.9	*16.5*
Buccal tube internal width	30	3.7 – 6.4	*10.2 – 14.6*	5.2	*12.3*	0.6	*1.0*	4.9	*11.7*
Ventral lamina length	30	21.6 – 29.7	*58.3 – 64.8*	26.1	*62.4*	2.0	*1.9*	25.2	*60.1*
Placoid lengths									
Macroplacoid 1	30	8.3 – 16.3	*22.1 – 39.4*	13.6	*32.5*	2.0	*4.0*	14.1	*33.7*
Macroplacoid 2	30	6.5 – 10.6	*17.3 – 24.8*	8.9	*21.4*	1.1	*1.8*	8.7	*20.8*
Microplacoid	30	3.3 – 5.6	*7.1 – 12.8*	4.1	*9.9*	0.5	*1.3*	4.3	*10.3*
Macroplacoid row	30	19.3 – 27.0	*52.1 – 62.5*	24.1	*57.7*	2.1	*2.7*	24.1	*57.5*
Placoid row	30	23.8 – 32.9	*65.0 – 75.1*	29.5	*70.6*	2.6	*2.7*	30.2	*72.1*
Claw 1 heights									
External primary branch	24	10.0 – 13.1	*22.8 – 33.1*	11.4	*27.5*	1.0	*2.3*	11.7	*27.9*
External secondary branch	24	7.6 – 12.5	*18.2 – 28.1*	9.1	*21.8*	1.2	*2.7*	10.1	*24.1*
Internal primary branch	26	9.5 – 12.4	*21.9 – 31.2*	11.0	*26.4*	0.8	*2.3*	11.6	*27.7*
Internal secondary branch	26	6.9 – 11.5	*16.0 – 26.5*	8.7	*21.0*	1.2	*2.8*	8.6	*20.5*
Claw 2 heights									
External primary branch	26	10.9 – 13.4	*25.2 – 34.3*	12.0	*28.8*	0.7	*2.3*	12.2	*29.1*
External secondary branch	26	7.8 – 11.8	*18.5 – 29.8*	9.5	*22.9*	1.1	*2.8*	10.2	*24.3*
Internal primary branch	28	9.9 – 12.7	*24.0 – 33.9*	11.2	*26.9*	0.7	*2.2*	11.6	*27.7*
Internal secondary branch	27	7.0 – 10.3	*17.3 – 27.0*	8.6	*20.7*	0.8	*2.2*	9.2	*22.0*
Claw 3 heights									
External primary branch	28	10.0 – 13.6	*24.5 – 35.8*	12.0	*29.0*	0.9	*2.8*	12.3	*29.4*
External secondary branch	26	7.1 – 11.3	*18.5 – 26.5*	9.4	*22.6*	1.0	*2.4*	10.1	*24.1*
Internal primary branch	26	9.5 – 12.9	*22.2 – 32.9*	11.3	*27.0*	0.9	*2.3*	11.5	*27.4*
Internal secondary branch	23	6.7 – 10.8	*16.0 – 26.2*	8.6	*20.7*	1.0	*2.6*	9.0	*21.5*
Claw 4 heights									
Anterior primary branch	16	11.6 – 13.8	*28.2 – 36.0*	13.0	*31.2*	0.6	*2.1*	12.4	*29.6*
Anterior secondary branch	16	7.8 – 10.5	*19.1 – 26.4*	9.3	*22.4*	0.7	*2.2*	10.0	*23.9*
Posterior primary branch	12	11.6 – 15.1	*27.6 – 38.5*	13.4	*32.2*	1.1	*3.0*	14.0	*33.4*
Posterior secondary branch	11	7.1 – 11.5	*16.5 – 27.4*	10.0	*23.5*	1.3	*3.1*	11.5	*27.4*

**Fig. 15:**
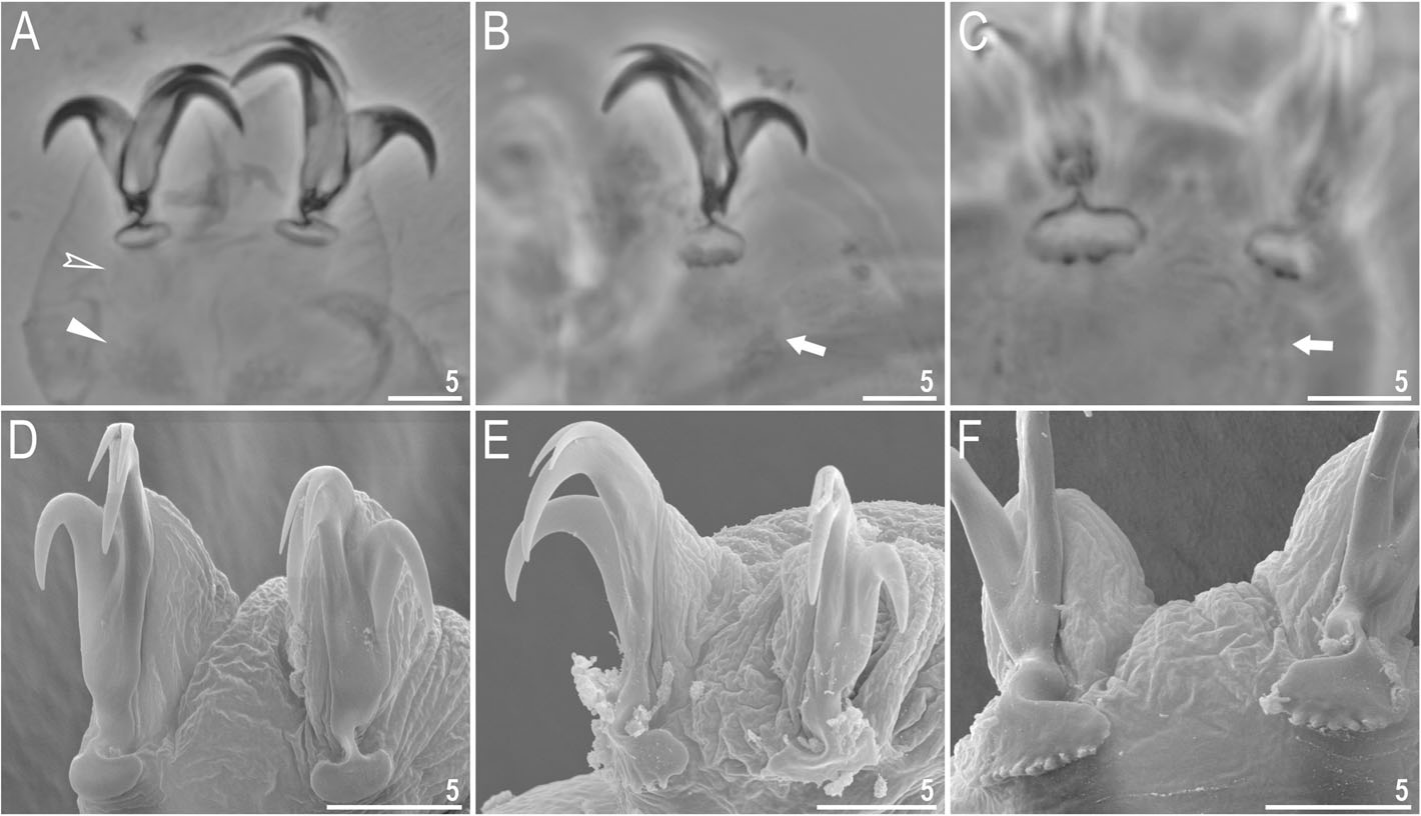
*Macrobiotus pseudopallarii* **sp. nov.** from Montenegro (ME.007) – claw morphology: **a–b** – claws III and IV seen with LCM; **C** – magnification of lunulae IV seen with LCM; **d–e** – claws III and IV seen with SEM; **f** – magnification of lunulae IV seen with SEM. Empty-indented arrowheads indicate dark circular areas under lunulae on the first three pairs of legs, filled flat arrowheads indicate cuticular bars above muscle attachments, and arrows indicate horseshoe structures connecting the anterior and posterior claws. Scale bars in μm

Claws slender, of the *hufelandi* type. Primary branches with distinct accessory points, a long common tract, and an evident stalk connecting the claw to the lunula (Fig. 15a–f). Lunulae on legs I–III smooth, whereas on leg IV gently dentate (Fig. 15a–f). Dark areas under each claw on legs I–III were faintly visible with LCM (Fig. 15a). Paired muscle attachments and faintly visible cuticular bars above them on legs I–III were often visible both with LCM (Fig. 15a) and SEM, whereas the horseshoe-shaped structure connecting anterior and posterior claws IV was visible only under LCM (Fig. 15b–c).

**Fig. 16:**
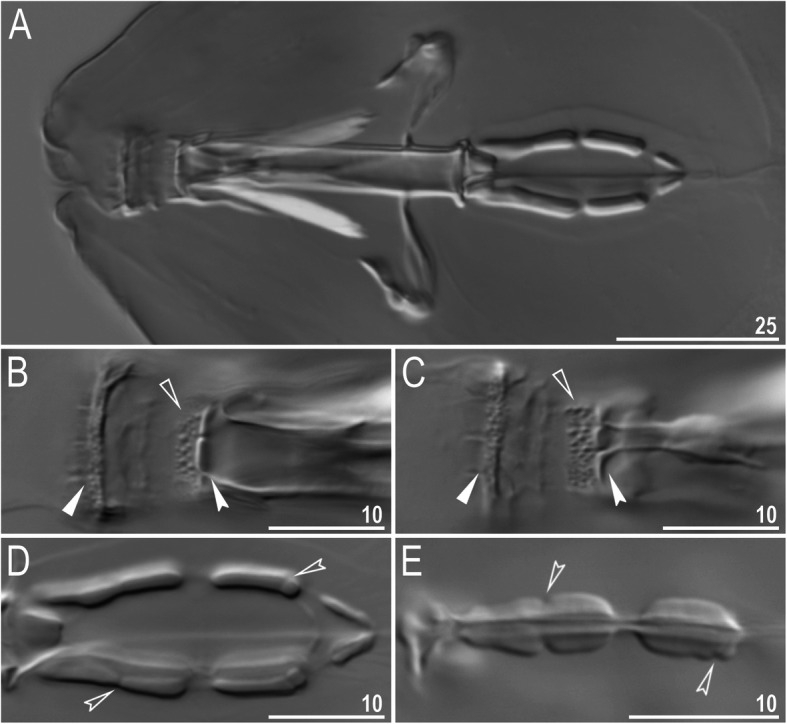
*Macrobiotus pseudopallarii* **sp. nov.** from Montenegro (ME.007) – buccal apparatus seen with LCM: **a** – an entire buccal apparatus; **b–c** – the oral cavity armature, dorsal and ventral teeth, respectively; **d–e** – placoid morphology, dorsal and ventral placoids, respectively. Filled flat arrowheads indicate the first band of teeth, empty flat arrowheads indicate the second band of teeth, filled indented arrowheads indicate the third band of teeth and empty indented arrowheads indicate central and subterminal constrictions in the first and second macroplacoid. Scale bars in μm

**Fig. 17:**
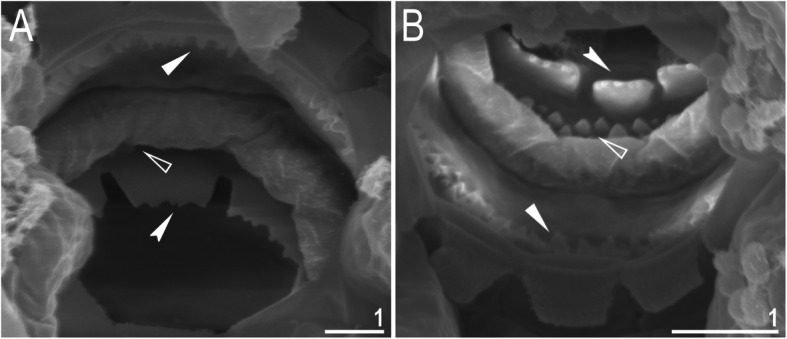
*Macrobiotus pseudopallarii* **sp. nov.** from Montenegro (ME.007) – the oral cavity armature seen with SEM: **a–b** – the oral cavity armature of a single specimen seen with SEM from different angles showing dorsal and ventral portion, respectively. Filled flat arrowheads indicate the first band of teeth, empty flat arrowheads indicate the second band of teeth, and filled indented arrowheads indicate the third band of teeth. Scale bars in μm

Mouth antero-ventral. Buccal apparatus of the *Macrobiotus* type (Fig. 16a), with the ventral lamina and ten peribuccal lamellae (Fig. 17a–b). The oral cavity armature was well developed and composed of three bands of teeth, all always clearly visible under LCM (Fig. 16b–c). The first band of teeth is composed of numerous small teeth visible with LCM as granules (Fig. 16b–c) and with SEM as cones (Fig. 17a–b), arranged in several rows, situated anteriorly in the oral cavity, just behind the bases of the peribuccal lamellae. The second band of teeth is situated between the ring fold and the third band of teeth and comprises 3–4 rows of teeth visible with LCM as granules (Fig. 16b–c) and with SEM as cones (Fig. 17a–b) but larger than those in the first band. The most anterior row of teeth within the second band comprises larger teeth than the subsequent posterior rows (Fig. 16b–c). The teeth of the third band are located within the posterior portion of the oral cavity, between the second band of teeth and the buccal tube opening (Figs. 16b–c, 17a–b). The third band of teeth is divided into the dorsal and ventral portions. Under both LCM and SEM, the dorsal teeth are seen as three distinct transverse ridges, whereas the ventral teeth appear as two separate lateral transverse ridges, between which one large tooth (sometimes circular in LCM) is visible (Figs. 16b–c, 17a–b). In SEM, teeth of the third band have indented margins (Fig. 17a–b). Pharyngeal bulb spherical, with triangular apophyses, two rod-shaped macroplacoids (2<1) and a microplacoid positioned close to them (i.e., the distance between the second macroplacoid and the microplacoid is shorter than the microplacoid length; Fig. 16d–e). The first macroplacoid is anteriorly narrowed and constricted in the middle, whereas the second has a subterminal constriction (Fig. 16d–e).

**Fig. 18:**
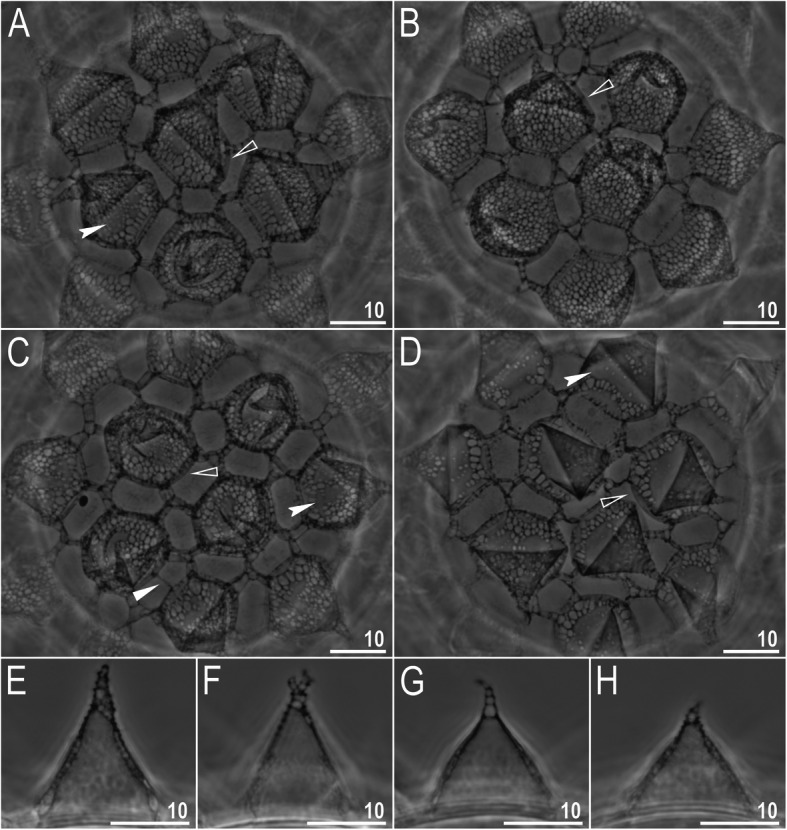
*Macrobiotus pseudopallarii* **sp. nov.** from Montenegro (ME.007) – eggs seen with LCM: **a–d** – surface under ×1000 magnification of four different eggs; **e–h** – midsections of four different egg processes. The filled flat arrowhead indicates thickening perpendicular to the process base that divides the areola in the middle, filled indented arrowheads indicate areas of the egg processes without a reticulation/labyrinthine layer, and empty flat arrowheads indicate irregular collars around process bases. Scale bars in μm

**Fig. 19:**
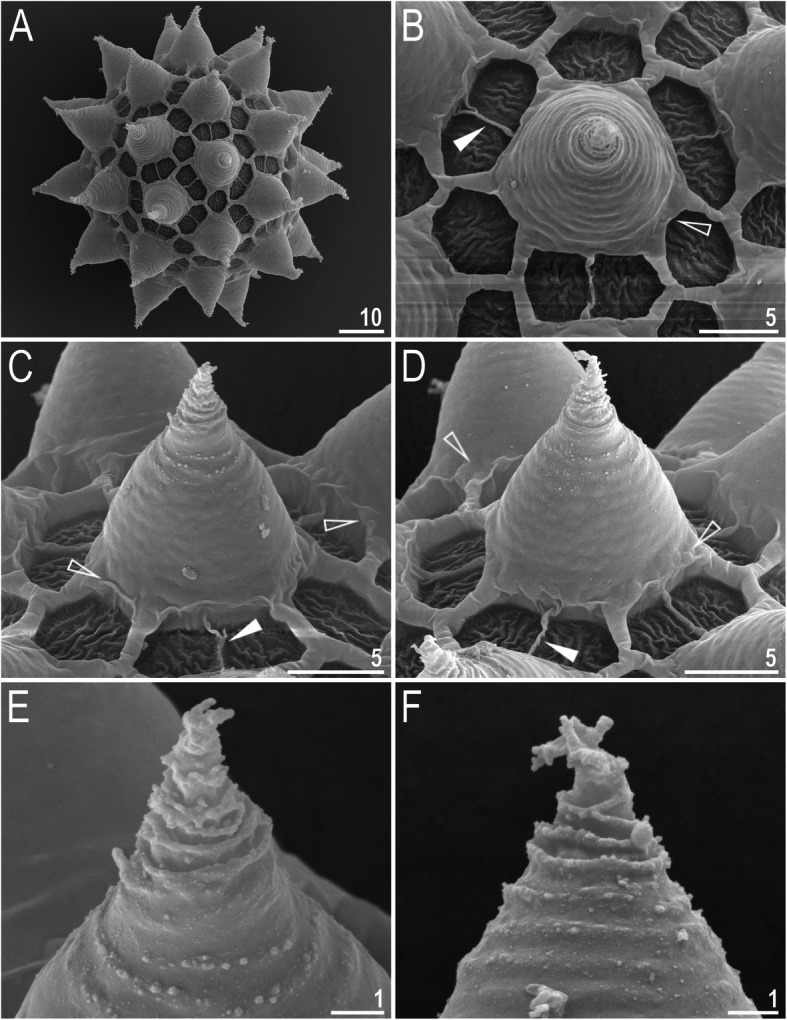
*Macrobiotus pseudopallarii* **sp. nov.** from Montenegro (ME.007) – eggs seen with SEM: **a** – entire view of the egg; **b–f** – details of the egg surface between processes, areolation and egg processes. Filled flat arrowheads indicate thickening perpendicular to the process base that divides the areola in the middle, empty flat arrowheads indicate irregular collars around process bases. Scale bars in μm

***Eggs*** *(measurements and statistics in* Table 9*):* Laid freely, white, spherical with conical processes surrounded by one row of areolae (Figs. 18a–h, 19a–f). In SEM, multiple rings of tight annulation on the entire process surface were visible (Fig. 19a–b), although in some processes, annulation was present only in the upper portion of the process (Fig. 19c–f) (annulation not visible with LCM because it was obscured by the eminent labyrinthine layer). The upper parts of the processes are covered by granulation, which to a varying extent is distributed on annuli, visible only under SEM (Fig. 19c–f). The labyrinthine layer within the process walls is present and visible as reticulation with circular/ellipsoidal meshes throughout the entire process (Fig. 18a–c). Only sometimes small areas without reticulation are present in some processes (Fig. 18a, c); however, very rarely, the reticulation can also be considerably reduced (Fig. 18d). The upper part of the process is often elongated into short flexible apices (Figs. 18e–h, 19c–f), which can be occasionally broken. The bottom part of the processes is flattened and extends into the six (only sometimes five) arms that form areolae rims (Figs. 18a–d, 19a–d). Each process is surrounded by six (only sometimes five) hexagonal areolae (Figs. 18a–d, 19a–d), which are occasionally falsely subdivided in the middle into two areolae by a thin thickening perpendicular to the process base (Figs. 18c, 19b–d). Areolae rims (walls) thick and usually flat (Fig. 19a–d), with the labyrinthine layer inside the rims visible as bubbles under LCM (Fig. 18a–d). Areolae rims also delimit the areolae at the bases of processes, which forms an irregular collar around process bases (Figs. 18a–d, 19a–d) and makes the process bases penta- or hexagonal in the top view (Figs. 18a–d, 19a–b). The areola surface has wrinkles that are faintly visible under LCM (Fig. 18a–d) but clearly visible under SEM (Fig. 19a–d). Micropores are present within the areolae, but they are distributed only around the areola rims and are usually absent in the central part of the areola (Fig. 19b–d).

**Table 9:** Measurements [in μm] of selected morphological structures of the eggs of *Macrobiotus pseudopallarii* **sp. nov.** from Montenegro (ME.007) mounted in Hoyer’s medium (N–number of eggs/structures measured, RANGE refers to the smallest and the largest structures among all measured specimens; SD–standard deviation)

CHARACTER	N	RANGE	MEAN	SD
Egg bare diameter	30	63.8 – 81.8	73.8	4.3
Egg full diameter	30	85.7 – 115.4	103.0	7.4
Process height	90	12.1 – 23.4	16.2	2.4
Process base width	90	10.2 – 21.0	15.5	1.9
Process base/height ratio	90	67% – 139%	97%	16%
Interprocess distance	90	2.0 – 8.4	4.5	1.5
Number of processes on the egg circumference	30	10 – 14	12.5	1.0

### Reproduction

**Fig. 20:**
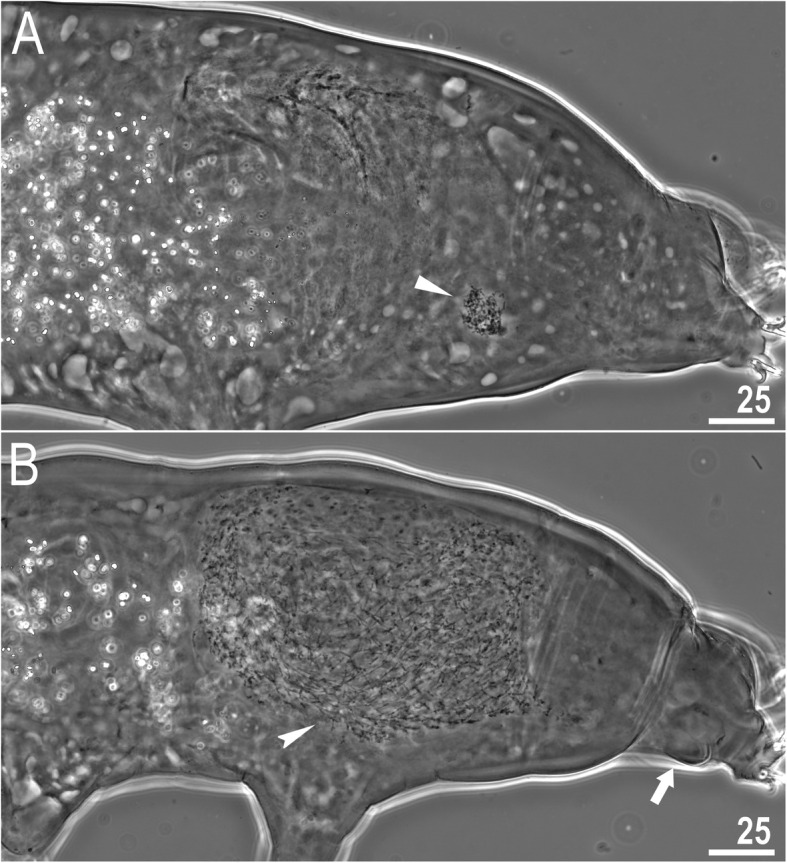
*Macrobiotus pseudopallarii* **sp. nov.** from Montenegro (ME.007) – reproduction (LCM): **a** – spermatheca (seminal vesicle) filled with spermatozoa and visible in females freshly mounted in Hoyer’s medium; **b** – testis filled with sperm visible in a male freshly mounted in Hoyer’s medium. The flat arrowhead indicates the female spermathecae, the indented arrowhead indicates the testis, and the arrow indicates the gibbosity on the IV leg. Scale bars in μm

The species is dioecious. Spermathecae in females as well as testes in males were found to be filled with spermatozoa, clearly visible under PCM up to 24 hours after mounting in Hoyer’s medium (Fig. 20a–b). The species exhibits secondary sexual dimorphism in the form of clearly visible lateral gibbosities on hind legs in males (Fig. 20b).

### DNA sequences and intraspecific genetic distances

- **18S rRNA** sequences (GenBank: MT809065–8), 798–897 bp long; 1 haplotype was found.
- **28S rRNA** sequences (GenBank: MT809077–80), 690–716 bp long; 1 haplotype was found.
- **ITS-2** sequences (GenBank: MT809090–2), 362 bp long; 2 haplotypes were found, separated by a p-distance of 0.6%.
- **COI** sequences (GenBank: MT807920–2), 630 bp long; 2 haplotypes were found, separated by a p-distance of 0.3%.

### Phenotypic differential diagnosis

By having the processes surrounded by 5–6 areolae, it resembles four other species of the *Macrobiotus pallarii* complex out of which two are newly described in this study. By the morphology of the animals and eggs, this species can be differentiated from the following:

- ***Macrobiotus pallarii***: by gently dentate lunulae IV (lunulae are faintly crenulated in *M. pallarii*) and a sparse granulation connecting the dense granulation patches between legs III and IV extending posteriorly to the granulation on legs IV (sparse granulation does not extend posteriorly to the granulation on legs IV in *M. pallarii*; see Fig. 1)*.*
- ***Macrobiotus ripperi*** **sp. nov.**: by gently dentate lunulae IV (lunulae are clearly dentate in *M. ripperi* **sp. nov.**), by the presence of two lateral patches of dense granulation between legs III and IV (dense granulation patches between legs III and IV are absent in *M. ripperi* **sp. nov.**; see Fig. 1) and by the presence of granulation on the egg process tips (granulation is absent in *M. ripperi* **sp. nov.**; character visible only under SEM).
- ***Macrobiotus margoae*** **sp. nov.**: by having an oral cavity armature well developed and composed of three bands of teeth visible under LCM (the oral cavity armature is less developed, and the first band of teeth are not visible under LCM in *M. margoae* **sp. nov.**), by having lunulae IV gently dentate (lunulae are clearly dentate in *M. margoae* **sp. nov.**), by the presence of two lateral patches of dense granulation between legs III and IV (dense granulation patches between legs III and IV are absent in *M. margoae* **sp. nov.**; see Fig. 1), by the presence of sparse dorsal granulation between legs III and IV (sparse granulation is absent in *M. margoae* **sp. nov.**; see Fig. 1), by a higher placoid row *pt* value (*65.0–75.1* in *M. pseudopallarii* **sp. nov.** vs. *51.1–60.6* in *M. margoae* **sp. nov.**), by the presence of meshes within the entire process walls (only small circular bubbles scattered randomly within the process are found in *M. margoae* **sp. nov.**), and by the presence of granulation on the egg process tips (granulation is absent in *M. margoae* **sp. nov.**; character visible only under SEM).
- ***Macrobiotus caymanensis***: by having an oral cavity armature well developed and composed of three bands of teeth visible under LCM (the oral cavity armature is less developed, and the first band of teeth is not visible under LCM in *M. caymanensis*), by the presence of granulation visible with LCM in all legs (granulation is not visible with LCM in *M. caymanensis*), by having lunulae IV gently dentate (the lunulae are smooth in *M. caymanensis*), by a higher placoid row *pt* value (*65.6–75.1* in *M. pseudopallarii* **sp. nov.** vs. *47.8–59.9* in *M. caymanensis*) and by the presence of meshes within the entire process (only small circular bubbles scattered randomly within the process walls are found in *M. caymanensis*).

### Genotypic differential diagnosis

Interspecific genetic p-distances between *M. pseudopallarii* **sp. nov.** and other species of the *M. pallarii* complex are as follows:

- **18S rRNA**: 0.0–1.4% (0.7% on average), with the most similar being *Macrobiotus pallarii* from Italy (MT809069–71) and the least similar being *Macrobiotus margoae* **sp. nov.** from the USA (MT809072–3).
- **28S rRNA**: 0.1–2.6% (1.7% on average), with the most similar being *Macrobiotus pallarii* from Italy (MT809081–3) and the least similar being *Macrobiotus margoae* **sp. nov.** from the USA (MT809084–5).
- **ITS-2**: 0.8–6.9% (5.1% on average), with the most similar being *Macrobiotus pallarii* from Italy (MT809094–6) and the least similar being *Macrobiotus margoae* **sp. nov.** H2 from USA (MT809097).
- **COI**: 14.0–22.5% (19.9% on average), with the most similar being *Macrobiotus pallarii* from Italy (MT807924–6) and the least similar being *Macrobiotus ripperi* **sp. nov.** Haplotype 3 (H3) from Finland (MT807930–2).

***Macrobiotus ripperi*** **Stec, Vecchi & Michalczyk, sp. nov.**

*Macrobiotus pallarii* in [81]

*Macrobiotus* cf. *pallarii* in [82]

*Macrobiotus* cf. *pallarii* PL.015 [19]

*Macrobiotus* cf. *pallarii* FI.066 [19]

**Zoobank:** urn:lsid:zoobank.org:act:EBA3D361-F598-4679-8503-11862D10240D

**Etymology:** Named after “Ripper”, the giant tardigrade-like creature from the TV series “Star Trek: Discovery” to celebrate the presence of tardigrades in pop culture.

**Material examined:** 202 animals and 77 eggs. Specimens were mounted on microscope slides in Hoyer’s medium (178 animals + 62 eggs), fixed on SEM stubs (20+15), and processed for DNA sequencing (4+0).

**Type locality:** 49°42′09″N, 21°55′53″E; 389 m asl: Poland: Malinówka, Yew Reserve; moss from forest; coll. April 2014 by Piotr Gąsiorek.

**Type depositories:** Holotype (slide PL.015.06 with 14 paratypes), 183 paratypes (slides: PL.015.07–14; SEM stub: 18.06) and 77 eggs (slides: PL.015.01–05; SEM stub: 18.06) were deposited at the Institute of Zoology and Biomedical Research, Jagiellonian University, Gronostajowa 9, 30-387, Kraków, Poland.

**Additional material:**

46 animals and 52 eggs. Specimens were mounted on microscope slides in Hoyer’s medium (42 animals + 52 eggs) and processed for DNA sequencing (4+0). Locality: 62°13′24.6″N, 25°46′20.4″E; 84 m asl: Finland: Jyväskylä, Graniitti, moss on rock at the entrance to a bunker; coll. 8^th^ Feb 2019 by Matteo Vecchi. Specimen depositories: Forty-two animals (slides: FI.066.05–08) and 52 eggs (slides: FI.066.01–04) were deposited at the Institute of Zoology and Biomedical Research, Jagiellonian University, Gronostajowa 9, 30-387, Kraków, Poland.

## Description of the new species

**Fig. 21:**
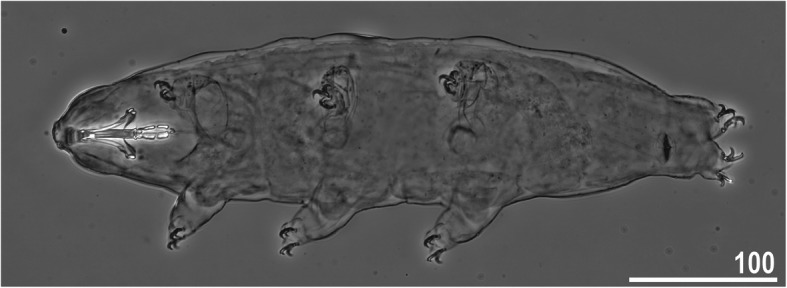
*Macrobiotus ripperi* **sp. nov.** from Poland (PL.015) – habitus, adult specimen in dorso-ventral projection (holotype). Scale bar in μm

**Fig. 22:**
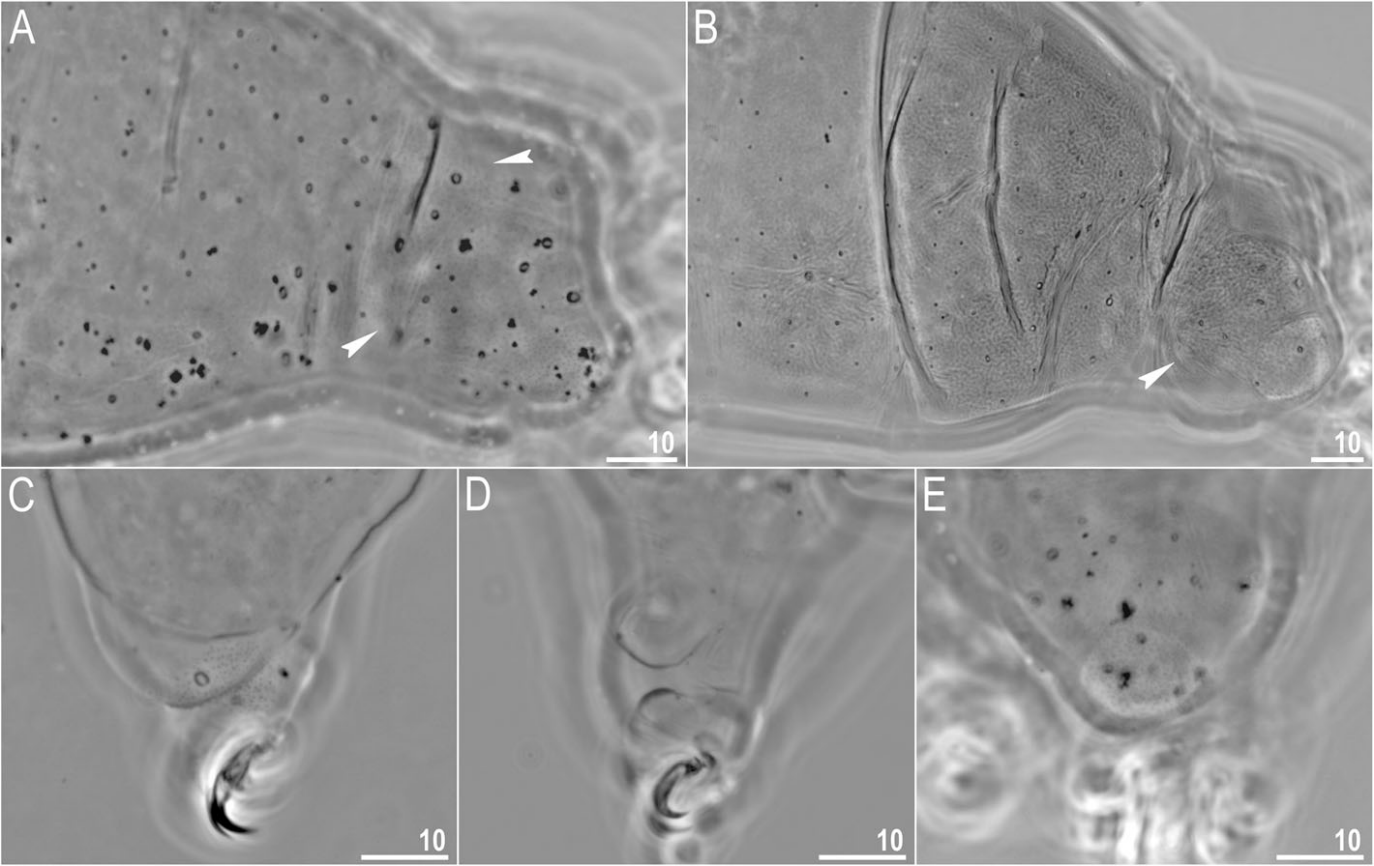
*Macrobiotus ripperi* **sp. nov.** from Poland (PL.015) – body and leg cuticle morphology seen with LCM: **a–b** – band of caudal granulation on the last body segment clearly visible in specimen with stretched cuticle (**a**) and hardly visible in specimen with wrinkled cuticle (**b**); **c** – granulation on the external surface of leg III; **d** – internal surface of leg II with evident pulvinus; **e** – granulation on dorsal surface of leg IV. Filled indented arrowheads indicate sparse granulation in the caudal band. Scale bar in μm

**Fig. 23:**
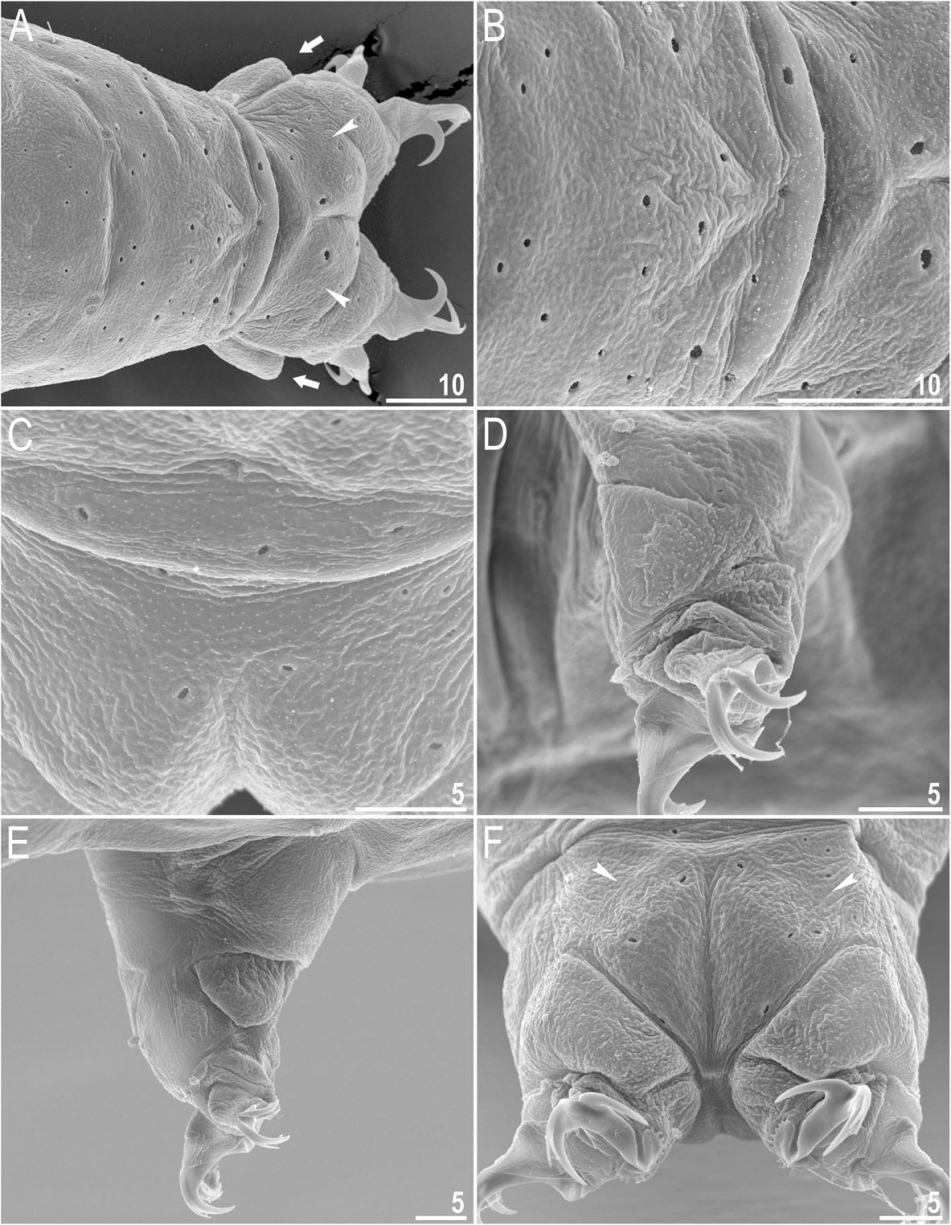
*Macrobiotus ripperi* **sp. nov.** from Poland (PL.015) – body and leg cuticle morphology seen with SEM: **a–c** – band of caudal granulation on the last body segment; **d** – granulation on the external surface of leg II; **e** – internal surface of leg III with evident pulvinus; **f** – granulation on dorsal surface of leg IV. Filled indented arrowheads indicate sparse granulation in the caudal band, arrows indicate lateral gibbosities on the IV leg – a male secondary sexual character. Scale bar in μm

***Animals*** *(measurements and statistics in* Table 10*):* In live animals, body almost transparent in smaller specimens and whitish in larger animals; transparent after fixation in Hoyer’s medium (Fig. 21). Eyes present in live animals and after fixation in Hoyer’s medium. Small round and oval cuticular pores (0.5–1.4 μm in diameter), visible under both LCM and SEM, scattered randomly throughout the entire body (Figs. 22a–e, 23a–f). Patches of fine granulation on the external surface of legs I–III as well as on the dorsal and dorsolateral sides of leg IV visible with LCM (Fig. 22c, e) and SEM (Fig. 23d, f). A pulvinus is present on the internal surface of legs I–III (Figs. 22d, 23e). In addition to the typical patches of leg granulation, sparse and uniformly distributed granulation is also present on the dorso- and latero-caudal surface of the last body segment (Figs. 22a, 23a–c). The sparse granulation connects with denser granulation patches on leg IV (Figs. 2, 22a, 23a–c). This granulation is slightly visible with LCM when the cuticle is wrinkled (Fig. 22b).

**Table 10:** Measurements [in μm] of selected morphological structures of individuals of *Macrobiotus ripperi* **sp. nov.** from Poland (PL.015) mounted in Hoyer’s medium (N–number of specimens/structures measured, RANGE refers to the smallest and the largest structures among all measured specimens; SD–standard deviation)

CHARACTER	N	RANGE		MEAN		SD		Holotype	
		μm	***pt***	μm	***pt***	μm	***pt***	μm	***pt***
Body length	30	384 – 491	*936 – 1233*	447	*1133*	28	*69*	444	*1223*
Buccal tube									
Buccal tube length	30	35.4 – 43.4	–	39.5	*–*	1.7	*–*	36.3	*–*
Stylet support insertion point	30	28.0 – 33.1	*75.3 – 79.1*	30.2	*76.6*	1.3	*0.9*	28.1	*77.4*
Buccal tube external width	30	5.1 – 6.6	*13.3 – 16.4*	6.0	*15.1*	0.4	*0.8*	5.7	*15.7*
Buccal tube internal width	30	3.9 – 5.0	*10.0 – 12.7*	4.5	*11.5*	0.3	*0.8*	4.2	*11.6*
Ventral lamina length	30	20.6 – 26.3	*54.1 – 63.6*	23.2	*58.7*	1.5	*2.5*	20.6	*56.7*
Placoid lengths									
Macroplacoid 1	30	10.0 – 14.4	*26.6 – 34.8*	12.3	*31.2*	1.0	*2.2*	11.5	*31.7*
Macroplacoid 2	30	6.8 – 9.7	*17.5 – 23.3*	7.8	*19.9*	0.7	*1.6*	7.1	*19.6*
Microplacoid	30	2.4 – 4.9	*6.5 – 12.4*	3.7	*9.4*	0.6	*1.3*	3.5	*9.6*
Macroplacoid row	30	19.4 – 25.3	*50.1 – 61.1*	21.7	*55.1*	1.3	*2.5*	20.3	*55.9*
Placoid row	30	24.0 – 29.1	*62.1 – 71.9*	26.8	*67.9*	1.5	*2.8*	24.7	*68.0*
Claw 1 heights									
External primary branch	29	9.4 – 12.5	*24.4 – 30.6*	11.1	*28.2*	0.8	*1.8*	11.1	*30.6*
External secondary branch	29	6.2 – 9.8	*16.0 – 24.1*	7.9	*20.1*	1.0	*2.3*	7.8	*21.5*
Internal primary branch	28	8.7 – 11.9	*22.7 – 30.5*	10.4	*26.5*	0.7	*1.7*	9.9	*27.3*
Internal secondary branch	28	6.3 – 9.0	*16.1 – 22.6*	7.5	*19.2*	0.5	*1.5*	7.5	*20.7*
Claw 2 heights									
External primary branch	29	10.0 – 13.3	*25.1 – 32.8*	11.7	*29.8*	0.8	*2.0*	11.3	*31.1*
External secondary branch	28	6.8 – 10.1	*17.5 – 26.4*	8.4	*21.4*	0.9	*2.2*	8.2	*22.6*
Internal primary branch	29	7.7 – 13.6	*19.9 – 34.1*	10.7	*27.2*	1.0	*2.4*	10.6	*29.2*
Internal secondary branch	30	6.2 – 9.7	*16.3 – 24.3*	7.9	*19.9*	0.9	*1.9*	7.8	*21.5*
Claw 3 heights									
External primary branch	30	10.0 – 13.0	*24.2 – 33.4*	11.7	*29.6*	0.9	*2.2*	11.4	*31.4*
External secondary branch	29	7.0 – 10.0	*17.5 – 25.2*	8.7	*21.9*	0.8	*2.0*	8.4	*23.1*
Internal primary branch	30	9.3 – 12.6	*24.2 – 32.2*	10.8	*27.5*	0.7	*1.9*	10.7	*29.5*
Internal secondary branch	30	5.8 – 9.6	*15.1 – 24.3*	7.9	*19.9*	1.0	*2.2*	7.9	*21.8*
Claw 4 heights									
Anterior primary branch	30	11.2 – 14.8	*28.2 – 36.1*	12.7	*32.3*	0.9	*2.4*	13.1	*36.1*
Anterior secondary branch	27	6.9 – 10.9	*18.2 – 26.9*	9.0	*22.8*	1.0	*2.4*	9.4	*25.9*
Posterior primary branch	28	12.4 – 15.8	*30.9 – 41.0*	14.0	*35.5*	1.0	*2.8*	14.9	*41.0*
Posterior secondary branch	13	7.0 – 10.4	*17.9 – 25.7*	8.9	*22.8*	1.0	*2.6*	?	*?*

**Fig. 24:**
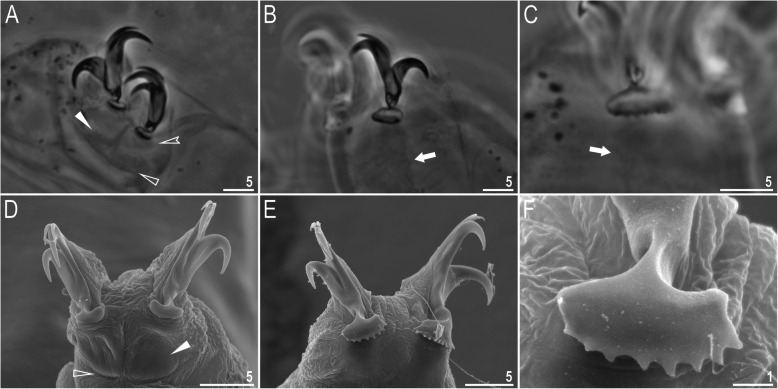
*Macrobiotus ripperi* **sp. nov.** from Poland (PL.015) – claw morphology: **a–b** – claws II and IV seen with LCM; **c** – magnification of lunulae IV seen with LCM; **d–e** – claws I and IV seen with SEM; **f** – magnification of lunulae IV seen with SEM. Empty indented arrowhead indicates dark circular areas under lunulae on the first three pairs of legs, filled flat arrowheads indicate cuticular bar above muscle attachments, empty flat arrowheads indicate double muscle attachments under claws, arrows indicate horseshoe structure connecting the anterior and the posterior claw. Scale bars in μm

Claws slender, of the *hufelandi* type. Primary branches with distinct accessory points, a long common tract, and an evident stalk connecting the claw to the lunula (Fig. 24a–f). Lunulae on legs I–III smooth, whereas on legs IV usually clearly dentate (Fig. 24a, c–f). Dentation was rarely absent or most likely not visible under LCM (Fig. 24b). Dark areas under each claw on legs I–III were faintly visible under LCM (Fig. 24a). Paired muscle attachments and faintly visible cuticular bars above them on legs I–III were often visible with both LCM (Fig. 24a) and SEM (Fig. 24d), whereas the horseshoe-shaped structure connecting anterior and posterior claw IV was visible only with LCM (Fig. 24b–c).

**Fig. 25:**
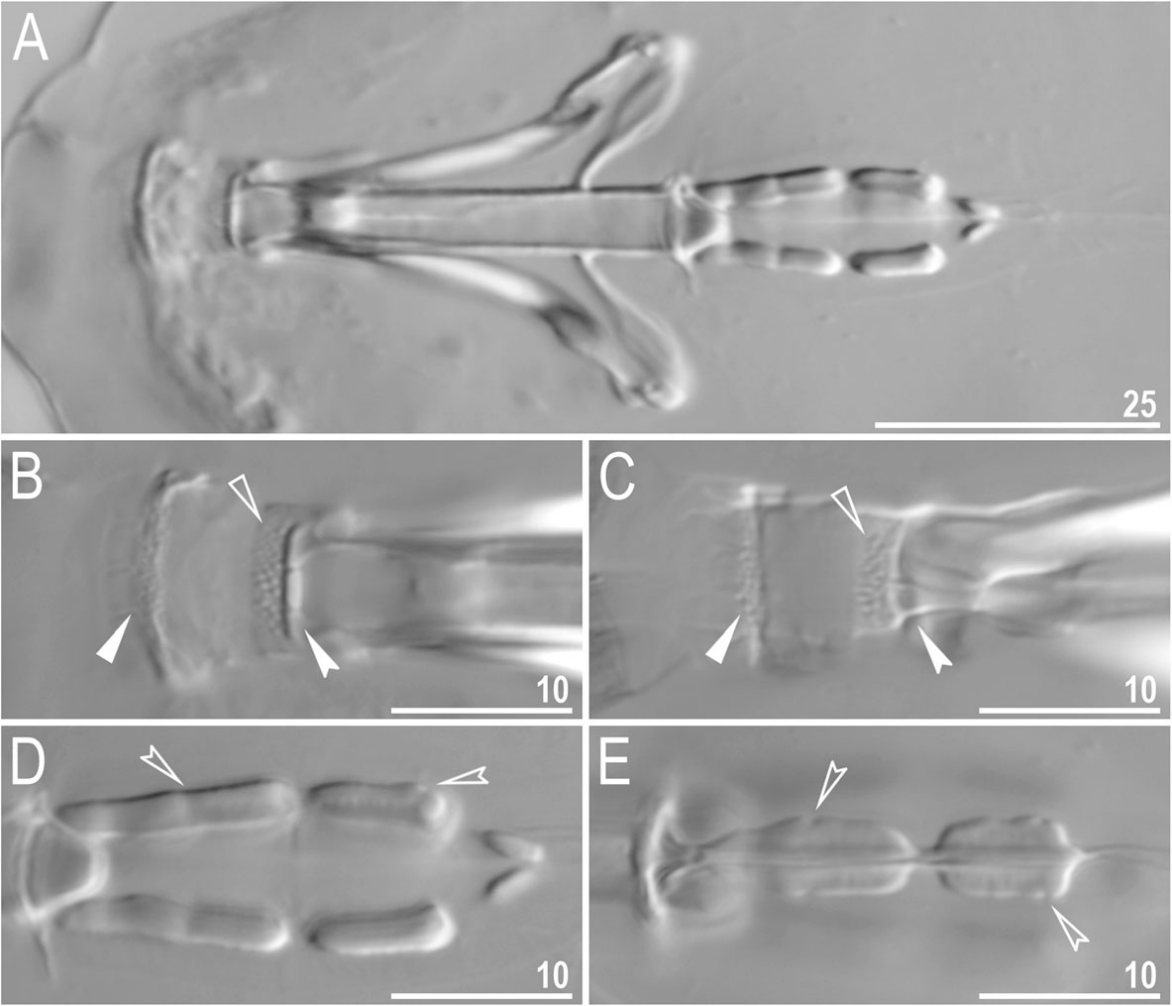
*Macrobiotus ripperi* **sp. nov.** from Poland (PL.015) – buccal apparatus seen with LCM: **a** – an entire buccal apparatus; **b–c** – the oral cavity armature, dorsal and ventral teeth, respectively; **d–e** – placoid morphology, dorsal and ventral placoids, respectively. Filled flat arrowheads indicate the first band of teeth, empty flat arrowheads indicate the second band of teeth, filled indented arrowheads indicate the third band of teeth, and empty indented arrowheads indicate central and subterminal constrictions in the first and second macroplacoids. Scale bars in μm

**Fig. 26:**
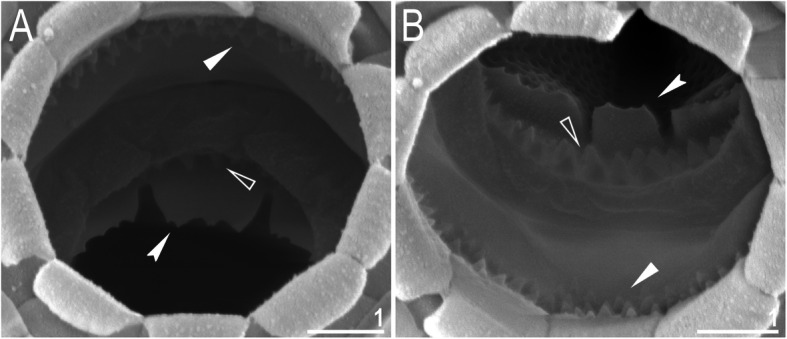
*Macrobiotus ripperi* **sp. nov.** from Poland (PL.015) – the oral cavity armature seen with SEM: **a–b** – the oral cavity armature of a single specimen seen with SEM from different angles showing dorsal and ventral portion, respectively. Filled flat arrowheads indicate the first band of teeth, empty flat arrowheads indicate the second band of teeth, and filled indented arrowheads indicate the third band of teeth. Scale bars in μm

Mouth antero-ventral. Buccal apparatus of the *Macrobiotus* type (Fig. 25a), with the ventral lamina and ten peribuccal lamellae (Fig. 26a–b). The oral cavity armature was well developed and composed of three bands of teeth, all always clearly visible under LCM (Fig. 25b–c). The first band of teeth is composed of numerous small teeth visible under LCM as granules (Fig. 25b–c) and under SEM as cones (Fig. 26a–b), arranged in several rows, situated anteriorly in the oral cavity, just behind the bases of the peribuccal lamellae. The second band of teeth is situated between the ring fold and the third band of teeth and comprises 3–4 rows of teeth visible under LCM as granules (Fig. 25b–c) and under SEM as cones (Fig. 26a–b) but larger than those in the first band. The most anterior row of teeth within the second band comprises larger teeth than the subsequent posterior rows (Fig. 25b–c). The teeth of the third band are located within the posterior portion of the oral cavity, between the second band of teeth and the buccal tube opening (Figs. 25b–c, 26a–b). The third band of teeth is divided into the dorsal and ventral portions. Under both LCM and SEM, the dorsal teeth are seen as three distinct transverse ridges, whereas the ventral teeth appear as two separate lateral transverse ridges, between which one large tooth (sometimes circular in LCM) is visible (Figs. 25b–c, 26a–b). In SEM, teeth of the third band have indented margins (Fig. 26a–b). Pharyngeal bulb spherical, with triangular apophyses, two rod-shaped macroplacoids (2<1) and a microplacoid positioned close to them (i.e., the distance between the second macroplacoid and the microplacoid is shorter than the microplacoid length; Fig. 25d–e). The first macroplacoid is anteriorly narrowed and constricted in the middle, whereas the second has a subterminal constriction (Fig. 25d–e).

**Fig. 27:**
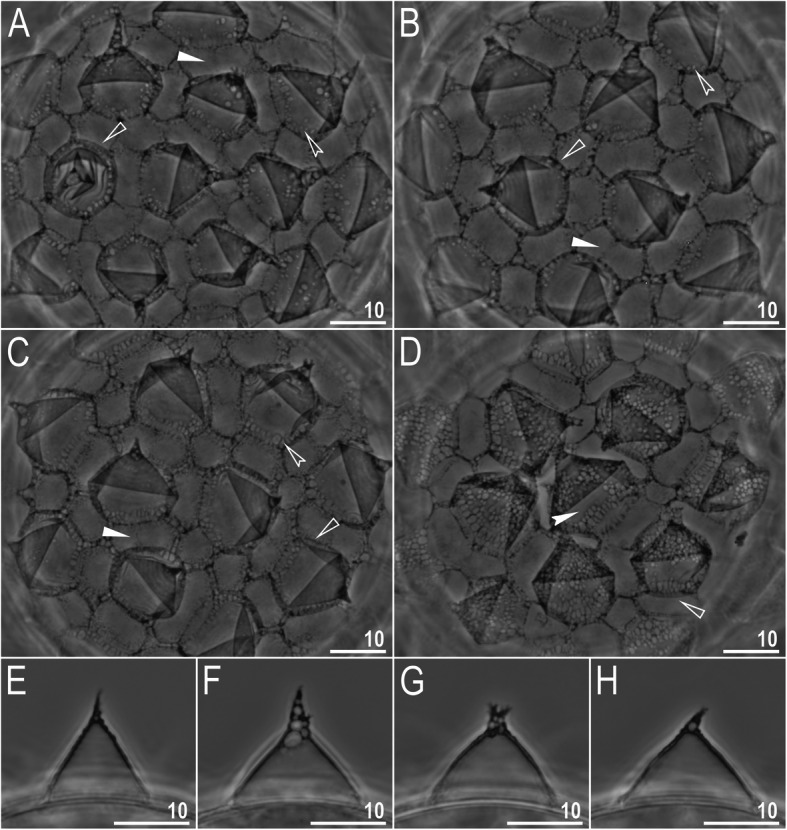
*Macrobiotus ripperi* **sp. nov.** from Poland (PL.015) – eggs seen in LCM: **a–d** – surface under ×1000 magnification of four different eggs; **e–h** – midsections of four different egg processes. The filled flat arrowhead indicates thickening perpendicular to the process base that divides the areola in the middle, the filled indented arrowhead indicates areas of the egg processes without a reticulation/labyrinthine layer, empty indented arrowheads indicate rings of the reticulation/labyrinthine layer in the bottom part of egg processes, and empty flat arrowheads indicate irregular collars around process bases. Scale bars in μm

**Fig. 28:**
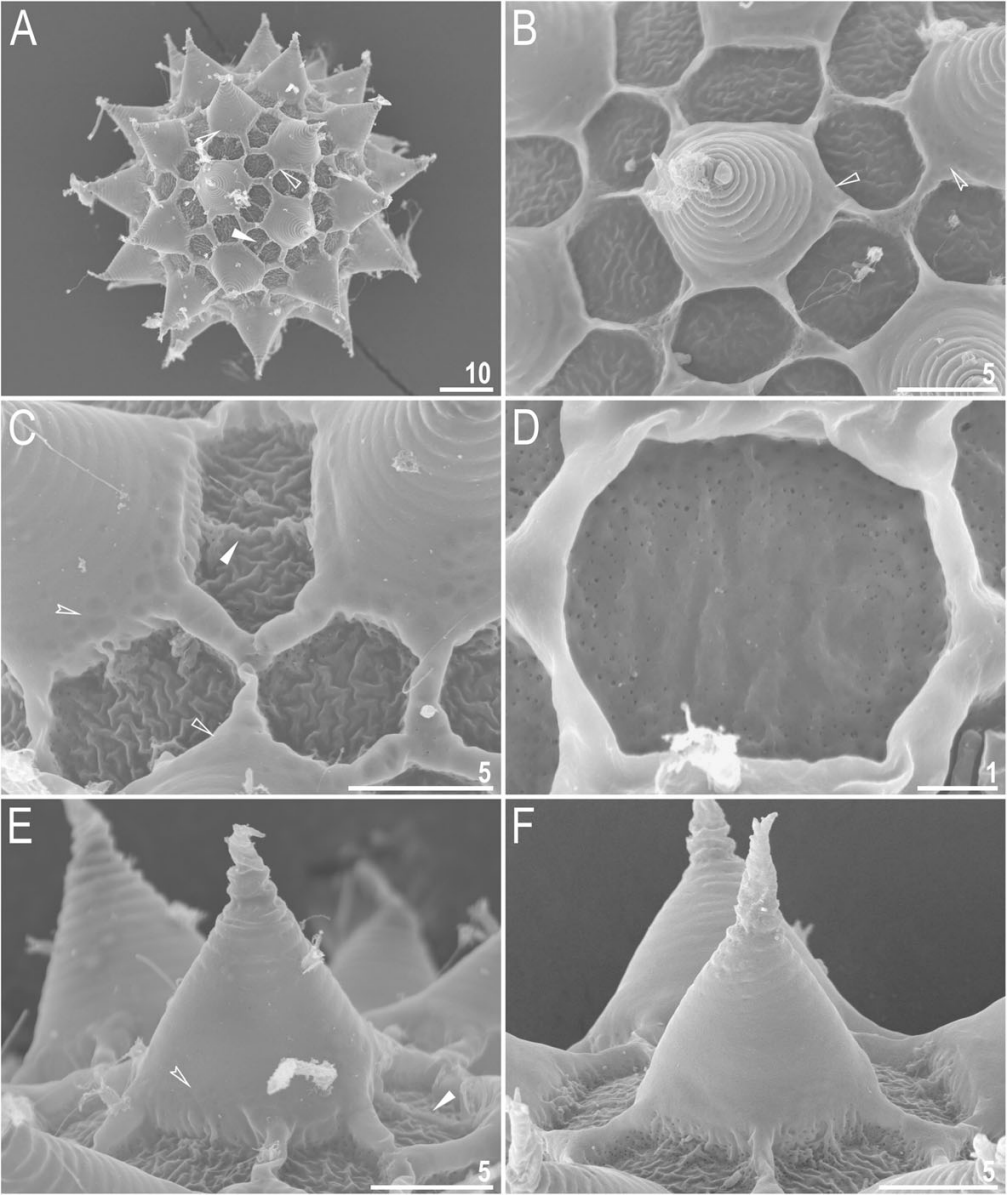
*Macrobiotus ripperi* **sp. nov.** from Poland (PL.015) – eggs seen with SEM: **a** – entire view of the egg; **b–f** – details of the egg surface between processes, areolation and egg processes. Filled flat arrowheads indicate thickening perpendicular to the process base, which divides the areola in the middle, empty flat arrowheads indicate irregular collar around process bases, and empty indented arrowheads indicate rings of the reticulation/labyrinthine layer in the bottom part of egg processes. Scale bars in μm

***Eggs*** *(measurements and statistics in* Table 11*):* Laid freely, white, spherical with conical processes surrounded by one row of areolae (Figs. 27a–h, 28a–f). In SEM, multiple rings of tight annulation on the entire process surface were visible (Fig. 28a–b), although in some processes, annulation was present only in the upper portion of the process (Fig. 28e–f) (annulation not visible in LCM because it was obscured by the eminent labyrinthine layer). The upper parts of the processes are smooth and not covered with granulation (Fig. 28e–f). The labyrinthine layer between the process walls was present and usually visible only in the bottom part of egg processes as one to three rows of meshes and some bubbles scattered randomly on the remaining process (Fig. 27a–c). These rings of reticulation can also sometimes be visible even with SEM (Fig. 28c, e). Sometimes well-developed reticulation with circular/ellipsoidal meshes is observed in the entire process but still with clearly visible places deprived of the reticulation (Fig. 27d). The upper part of the process surface is often elongated into short flexible apices that can be occasionally broken and that can sometimes be bifurcated and have bubble-like structures (Figs. 27e–h, 28a, e–f). The base of the processes extends into the six (only sometimes five) arms that form areolae rims (Figs. 27a–d, 28a–f). Each process is surrounded by six (only sometimes five) hexagonal areolae (Figs. 27a–d, 28a–d), which are occasionally falsely subdivided in the middle into two areolae by a thin thickening perpendicular to the process base (Figs. 27a–c, 28a, c). Areolae rims (walls) are usually thick and flat (Fig. 28a, c–d), but sometimes they can also be very thin (Fig. 28b), with the labyrinthine layer inside the rims visible as bubbles under LCM (Fig. 27a–d). The areola surface has wrinkles that are faintly visible under LCM (Fig. 27a–d) but clearly visible under SEM (Fig. 28a–f). Micropores are present within the areolae, but they are distributed only around the areolae rims and usually absent in the central part of the areola (Fig. 28b–f).

**Table 11:** Measurements [in μm] of selected morphological structures of the eggs of *Macrobiotus ripperi* **sp. nov.** from Poland (PL.015) mounted in Hoyer’s medium (N–number of eggs/structures measured, RANGE refers to the smallest and the largest structure among all measured specimens; SD–standard deviation)

CHARACTER	N	RANGE	MEAN	SD
Egg bare diameter	30	70.7 – 82.1	76.6	3.0
Egg full diameter	30	89.2 – 108.8	99.4	4.4
Process height	90	8.8 – 16.7	13.2	1.7
Process base width	90	12.1 – 18.8	15.3	1.5
Process base/height ratio	90	83% – 177%	118%	18%
Interprocess distance	90	3.6 – 9.0	5.6	1.2
Number of processes on the egg circumference	28	11 – 13	11.9	0.7

### Reproduction

**Fig. 29:**
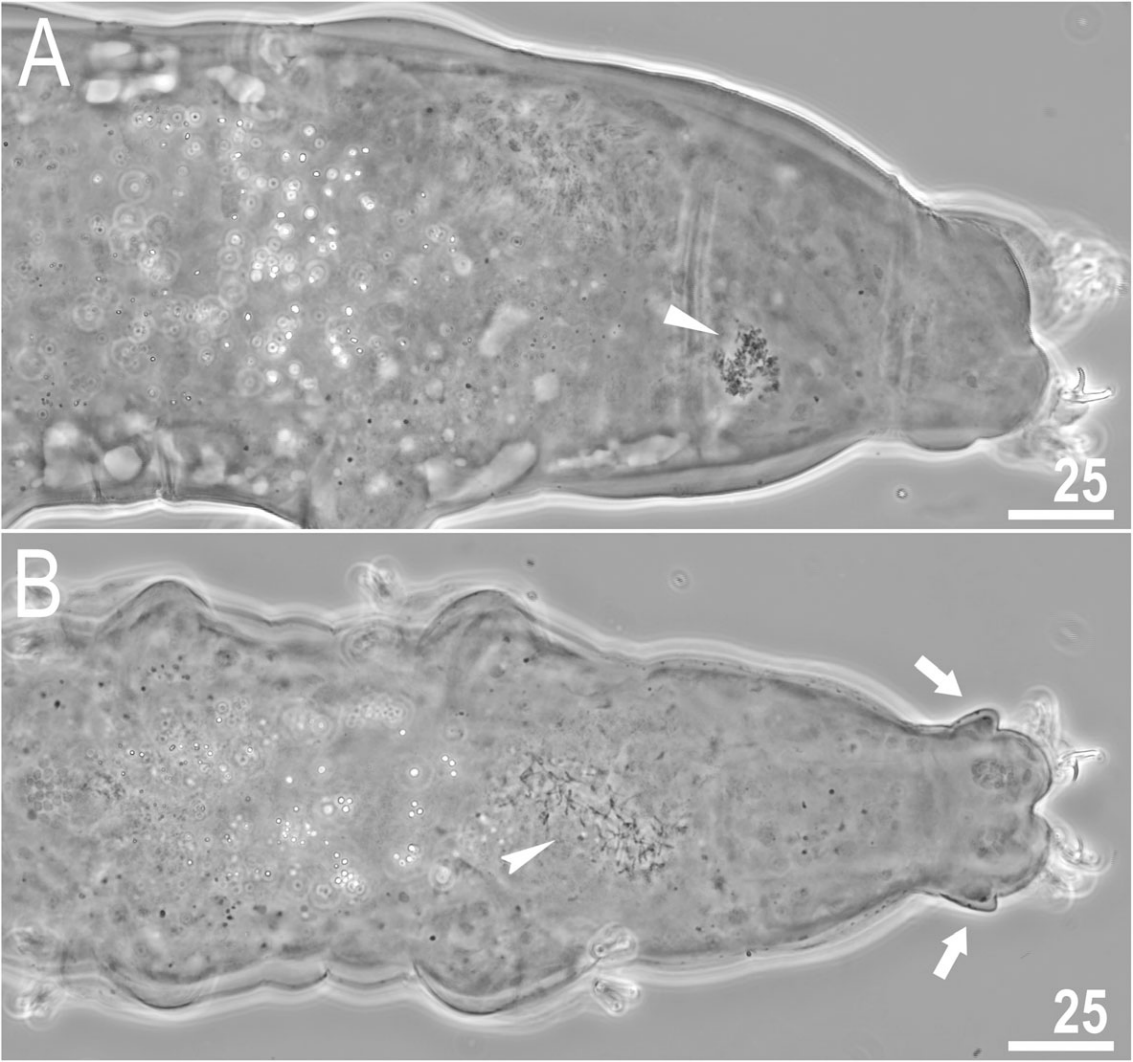
*Macrobiotus ripperi* **sp. nov.** from Poland (PL.015) – reproduction (LCM): **a** – spermatheca (seminal vesicle) filled with spermatozoa and visible in females freshly mounted in Hoyer’s medium; **b** – testis filled with sperm visible in a male freshly mounted in Hoyer’s medium. The flat arrowhead indicates the female spermathecae, the indented arrowhead indicates the testis, and arrows indicate gibbosities on the IV legs. Scale bars in μm

The species is dioecious. Spermathecae in females as well as testes in males were found to be filled with spermatozoa, clearly visible under PCM up to 24 hours after mounting in Hoyer’s medium (Fig. 29a–b). The species exhibits secondary sexual dimorphism in the form of clearly visible lateral gibbosities on hind legs in males (Fig. 29b).

### DNA sequences and intraspecific genetic distances

- **18S rRNA** sequences (GenBank: MT809074–9), 987 bp long; 1 haplotype was found.
- **28S rRNA** sequences (GenBank: MT809086–9), 716 bp long; 1 haplotype was found.
- **ITS-2** sequences (GenBank: MT809100–2; MT807929–32), 360 bp long; 2 haplotypes were found, separated by a p-distance of 0.8%.
- **COI** sequences (GenBank: MT807933–5; MT809103–5) were 630 bp long; 3 haplotypes were found, with p-distances ranging from 1.0% to 2.7%.

### Phenotypic differential diagnosis

By having the processes surrounded by 5–6 areolae, it resembles four other species of the *Macrobiotus pallarii* complex out of which two are newly described in this study. By the morphology of the animals and eggs, this species can be differentiated from the following:

- ***Macrobiotus pallarii***: by lunulae IV being dentate (the lunulae are only faintly crenulated in *M. pallarii*), by the absence of two lateral patches of dense granulation between legs III and IV (dense granulation patches between legs III and IV are present in *M. pallarii*; see Fig. 1), by the presence of a sparse granulation connecting the dense granulation patches between legs III and IV extending posteriorly to the granulation on legs IV (sparse granulation does not extend posteriorly to the granulation on legs IV in *M. pallarii*; see Fig. 1) and by and the absence of granulation on the egg processes tips (granulation is present in *M. pallarii*.; character visible only under SEM).
- ***Macrobiotus pseudopallarii*** **sp. nov.**: by lunulae IV being dentate (the lunulae are only gently dentate in *M. pseudopallarii* **sp. nov.**), by the absence of two lateral patches of dense granulation between legs III and IV (dense granulation patches between legs III and IV are present in *M. pseudopallarii* **sp. nov.**; see Fig. 1) and by and the absence of granulation on the egg process tips (granulation present is in *M. pseudopallarii* **sp. nov.**; character visible only under SEM).
- ***Macrobiotus margoae*** **sp. nov.:** by having an oral cavity armature that is well developed and composed of three bands of teeth visible with LM (the oral cavity armature is less developed, and the first band of teeth not visible with LM in *M. margoae* **sp. nov**), by the presence of sparse dorsal granulation between legs III and IV (sparse granulation is absent in *M. margoae* **sp. nov.**; see Fig. 1).
- ***Macrobiotus caymanensis***: by having an oral cavity armature well developed and composed of three bands of teeth visible with LCM (the oral cavity armature is less developed, and the first band of teeth is not visible with LCM in *M. caymanensis*), by the presence of granulation visible with LCM in all legs (granulation is not visible in *M. caymanensis*), by the presence of a sparse dorsal granulation between legs III and IV (sparse granulation is absent or not visible in *M. caymanensis*) and by lunulae IV being dentate (the lunulae are smooth in *M. caymanensis*).

### Genotypic differential diagnosis

Interspecific genetic p-distances between *M. ripperi* **sp. nov.** and other species of the *M. pallarii* complex are as follows:

- **18S rRNA**: 1.0–1.2% (1.1% on average), with the most similar being *Macrobiotus pallarii* from Italy (MT809069–71) and *Macrobiotus pseudopallarii* **sp. nov.** from Montenegro (MT809065–6), and the least similar being *Macrobiotus margoae* **sp. nov.** from the USA (MT809072–3).
- **28S rRNA**: 2.1–2.5% (2.3% on average), with the most similar being *Macrobiotus margoae* **sp. nov.** from the USA (MT809084–5), and the least similar being *Macrobiotus pallarii* from Italy (MT809081-3).
- **ITS-2**: 5.3–8.6% (6.8% on average), with the most similar (to H2) being *Macrobiotus pallarii* from Italy (MT809094–6) and the least similar (to H1) being *Macrobiotus margoae* **sp. nov.** H2 from the USA (MT809097).
- **COI**: 18.6–22.7% (21.4% on average), with the most similar (to H1) being *Macrobiotus pallarii* from Italy (MT807924–6) and the least similar (to H3) being *Macrobiotus margoae* **sp. nov.** from the USA (MT807927–8).

***Macrobiotus margoae*** **Stec, Vecchi & Bartels, sp. nov.**

*Macrobiotus* n. sp. 9 in [83, 84]

*Macrobiotus* sp. in [85]

*Macrobiotus pallarii* in [86–88]

*Macrobiotus* cf. *pallarii* US.057 [19]

**Zoobank:** urn:lsid:zoobank.org:act:ECD1BAC6-AC99-4991-B901-F0E5BDD92CC0

**Etymology:** This species is named in honor of PB's life partner, Margo Nottoli, who has shown tardigrade-like tolerance toward pressure, heat, and stress through 20 years of her husband's research obsession.

**Material examined:** 101 animals and 36 eggs. Specimens were mounted on microscope slides in Hoyer’s medium (90 animals + 29 eggs), fixed on SEM stubs (7+7), and processed for DNA sequencing (4+0).

**Type locality:** 35°35′7.84″N, 83°4′26.47″W; 1492 m asl: United States: Great Smoky Mountains National Park, Purchase Knob; Haywood County, North Carolina; moss on a tree trunk in the forest; coll. Nov 2018 by Nate Gross & Mackenzie McClay.

**Type depositories:** Holotype (slide US.057.07 with 1 paratype), 95 paratypes (slides: US.057.02–06; SEM stub: 18.12) and 36 eggs (slides: US.057.01, 08–09; SEM stub: 18.12) are deposited at the Institute of Zoology and Biomedical Research, Jagiellonian University, Gronostajowa 9, 30-387, Kraków, Poland.

## Description of the new species

**Fig. 30:**
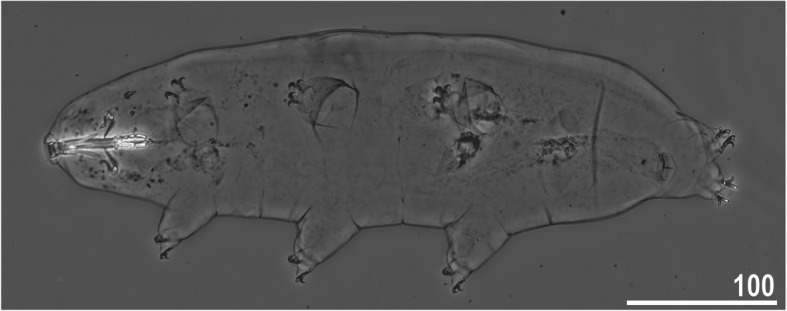
*Macrobiotus margoae* **sp. nov.** from the USA (US.057) – habitus, adult specimen in dorsoventral projection (holotype). Scale bar in μm

**Fig. 31:**
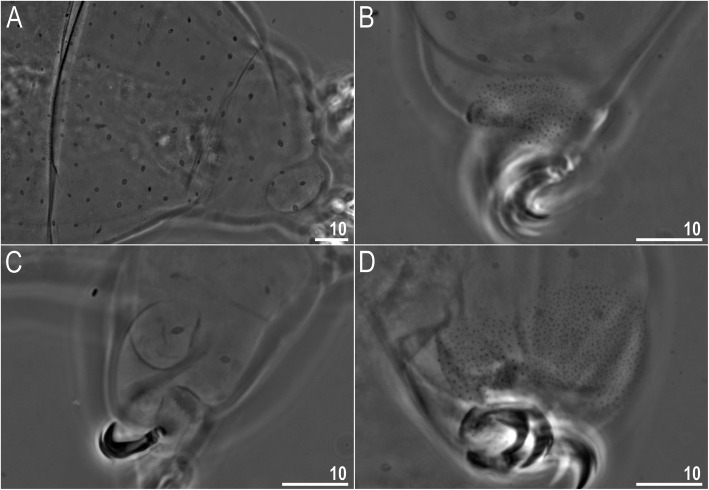
*Macrobiotus margoae* **sp. nov.** from USA (US.057) – body and leg cuticle morphology seen with LCM: **a** – cuticle on the last body segment without caudal band of granulation; **b** – granulation on the external surface of leg III; **c** – internal surface of leg I with evident pulvinus; **d** – granulation on dorsal surface of leg IV. Scale bar in μm

**Fig. 32:**
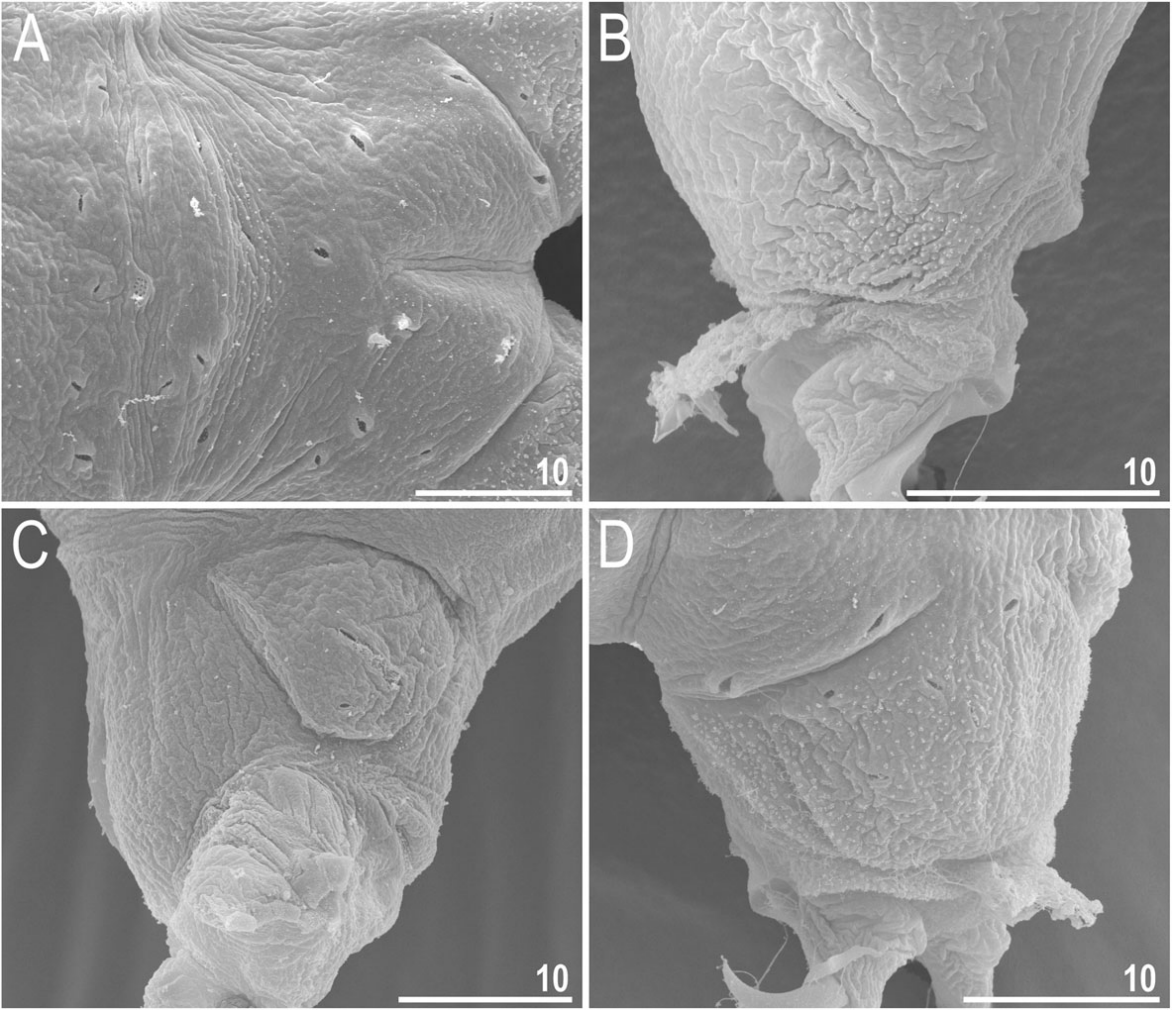
*Macrobiotus margoae* **sp. nov.** from USA (US.057) – body and leg cuticle morphology seen with SEM: **a** – cuticle on the last body segment without caudal band of granulation; **b** – granulation on the external surface of leg III; **c** – internal surface of leg III with evident pulvinus; **d** – granulation on dorsal surface of leg IV. Scale bar in μm.

***Animals*** *(measurements and statistics in* Table 12*):* In live animals, body almost transparent in smaller specimens and whitish in larger animals; transparent after fixation in Hoyer’s medium (Fig. 30). Eyes present in live animals and after fixation in Hoyer’s medium. Small round and oval cuticular pores (0.7–1.7 μm in diameter), visible under both LCM and SEM, scattered randomly throughout the entire body (Figs. 31a–d, 32a–d). Patches of fine granulation on the external surface of legs I–III as well as on the dorsal and dorsolateral sides of leg IV visible under LCM (Fig. 2, 31b, d) and SEM (Fig. 32b, d). A pulvinus is present on the internal surface of legs I–III (Figs. 31c, 32c). In addition to the typical patches of leg granulation, other types of cuticular granulation are absent.

**Table 12:** Measurements [in μm] of selected morphological structures of individuals of *Macrobiotus margoae* **sp. nov.** from the USA (US.057) mounted in Hoyer’s medium (N–number of specimens/structures measured, RANGE refers to the smallest and largest structures among all measured specimens; SD–standard deviation)

CHARACTER	N	RANGE		MEAN		SD		Holotype	
		μm	***pt***	μm	***pt***	μm	***pt***	μm	***pt***
Body length	30	239 – 443	*690 – 1220*	338	*973*	47	*114*	443	*1220*
Buccal tube									
Buccal tube length	30	29.4 – 41.9	–	34.8	*–*	2.9	*–*	36.3	*–*
Stylet support insertion point	30	23.4 – 32.7	*76.2 – 79.8*	27.1	*77.8*	2.3	*1.0*	28.4	*78.2*
Buccal tube external width	30	3.1 – 6.3	*9.5 – 16.8*	4.9	*14.1*	0.7	*1.7*	6.1	*16.8*
Buccal tube internal width	30	2.8 – 4.9	*8.6 – 15.7*	3.8	*11.0*	0.5	*1.4*	4.2	*11.6*
Ventral lamina length	30	18.4 – 25.2	*55.0 – 65.8*	21.6	*62.0*	1.9	*2.7*	20.7	*57.0*
Placoid lengths									
Macroplacoid 1	30	6.3 – 11.8	*20.0 – 29.9*	9.0	*25.8*	1.2	*2.2*	10.1	*27.8*
Macroplacoid 2	30	4.1 – 7.1	*11.1 – 19.7*	5.8	*16.6*	0.9	*2.1*	7.1	*19.6*
Microplacoid	30	1.7 – 6.1	*4.8 – 16.8*	3.0	*8.5*	0.8	*2.0*	3.5	*9.6*
Macroplacoid row	30	11.7 – 20.3	*40.0 – 55.9*	16.0	*45.9*	2.0	*3.4*	20.3	*55.9*
Placoid row	30	16.0 – 24.7	*52.1 – 68.0*	19.8	*56.8*	2.1	*3.1*	24.7	*68.0*
Claw 1 heights									
External primary branch	25	6.3 – 11.1	*20.4 – 30.6*	8.5	*24.2*	0.9	*2.0*	11.1	*30.6*
External secondary branch	25	5.0 – 8.0	*16.6 – 24.7*	6.9	*20.0*	0.7	*2.2*	7.8	*21.5*
Internal primary branch	24	6.8 – 9.9	*18.7 – 27.3*	8.0	*22.9*	0.7	*1.9*	9.9	*27.3*
Internal secondary branch	22	5.5 – 7.5	*14.8 – 21.4*	6.5	*18.7*	0.5	*1.8*	7.5	*20.7*
Claw 2 heights									
External primary branch	24	7.7 – 10.4	*20.3 – 28.6*	8.9	*25.5*	0.7	*2.0*	10.1	*27.8*
External secondary branch	18	5.9 – 8.1	*16.5 – 25.2*	7.1	*20.1*	0.7	*2.4*	7.6	*20.9*
Internal primary branch	22	6.4 – 9.7	*18.3 – 27.8*	8.3	*23.8*	0.8	*2.2*	9.5	*26.2*
Internal secondary branch	20	5.4 – 7.7	*15.3 – 22.2*	6.7	*19.3*	0.6	*2.1*	7.5	*20.7*
Claw 3 heights									
External primary branch	22	7.2 – 10.3	*21.0 – 27.6*	8.9	*25.2*	0.9	*2.0*	10.0	*27.5*
External secondary branch	24	5.8 – 8.2	*16.8 – 23.9*	7.1	*20.3*	0.7	*2.1*	7.9	*21.8*
Internal primary branch	23	7.1 – 9.4	*20.0 – 28.0*	8.3	*23.6*	0.7	*1.9*	8.2	*22.6*
Internal secondary branch	18	5.4 – 8.0	*14.4 – 22.3*	6.5	*18.3*	0.8	*2.2*	?	*?*
Claw 4 heights									
Anterior primary branch	16	8.2 – 11.3	*22.7 – 31.1*	9.5	*27.6*	0.8	*2.2*	11.3	*31.1*
Anterior secondary branch	13	6.1 – 8.9	*16.4 – 24.7*	7.4	*21.2*	0.9	*2.6*	8.9	*24.5*
Posterior primary branch	14	8.4 – 11.5	*24.5 – 32.6*	9.9	*28.8*	0.8	*2.6*	11.5	*31.7*
Posterior secondary branch	18	6.4 – 9.0	*20.1 – 26.7*	7.6	*22.0*	0.8	*1.7*	?	*?*

**Fig. 33:**
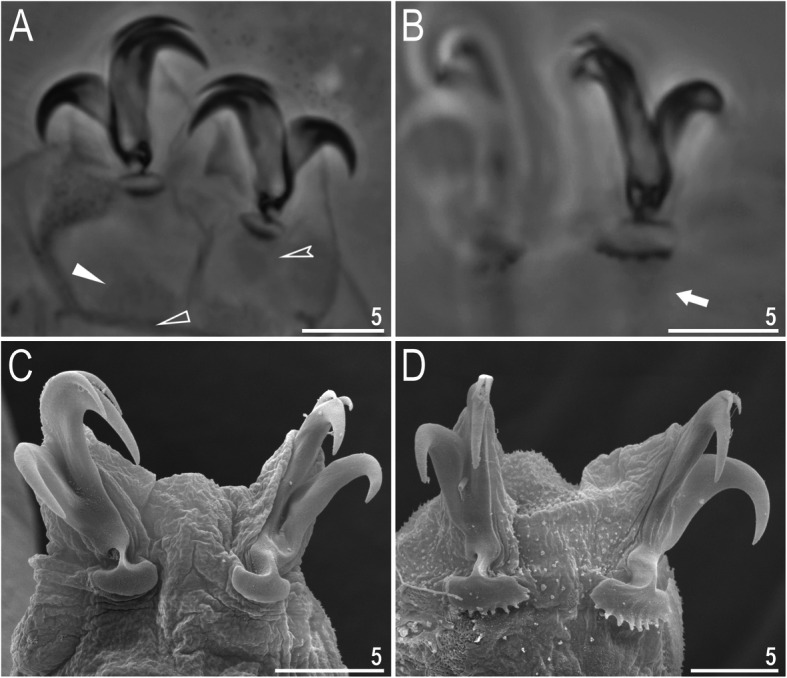
*Macrobiotus margoae* **sp. nov.** from USA (US.057) – claw morphology: **a–b** – claws II and IV seen with LCM; **c–d** – claws III and IV seen with SEM. Empty indented arrowhead indicates dark circular areas under lunulae on the first three pairs of legs, filled flat arrowhead indicates cuticular bar above muscle attachments, empty flat arrowhead indicates double muscle attachments under claws, arrow indicates horseshoe structure connecting the anterior and the posterior claws. Scale bars in μm

Claws slender, of the *hufelandi* type. Primary branches with distinct accessory points, a long common tract, and an evident stalk connecting the claw to the lunula (Fig. 33a–d). Lunulae on legs I–III smooth, whereas those on legs IV clearly dentate (Fig. 33a–d). Dark areas under each claw on legs I–III faintly visible under LCM (Fig. 33a). Paired muscle attachments and internal strengthening above them on legs I–III were often visible under both LCM (Fig. 24a) and SEM, whereas the horseshoe-shaped structure connecting anterior and posterior claws IV was visible only with LCM (Fig. 33b).

**Fig. 34:**
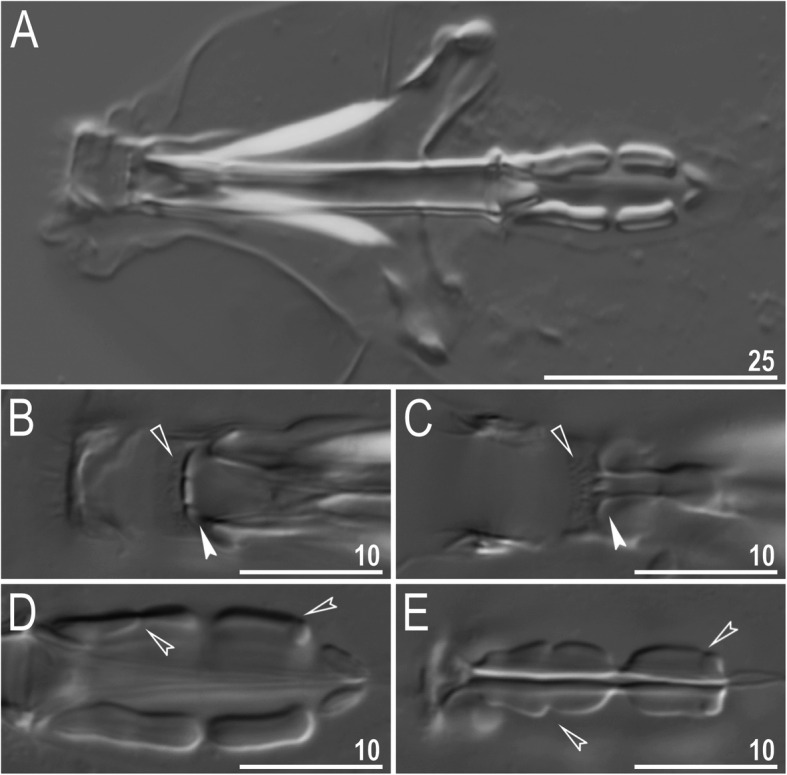
*Macrobiotus margoae* **sp. nov.** from USA (US.057) – buccal apparatus seen with LCM: **a** – an entire buccal apparatus; **b–c** – the oral cavity armature, dorsal and ventral teeth, respectively; **d–e** – placoid morphology, dorsal and ventral placoids, respectively. Empty flat arrowheads indicate the second band of teeth, filled indented arrowheads indicate the third band of teeth, and empty indented arrowheads indicate central and subterminal constrictions in the first and second macroplacoid. Scale bars in μm

**Fig. 35:**
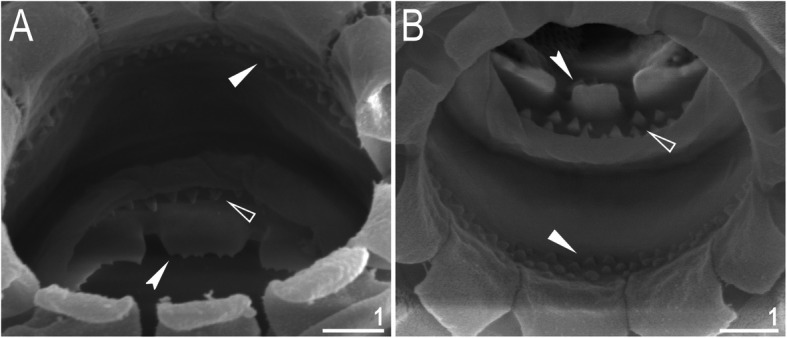
*Macrobiotus margoae* **sp. nov.** from USA (US.057) – the oral cavity armature seen with SEM: **a–b** – the oral cavity armature of a single specimen seen in SEM from different angles showing dorsal and ventral portion, respectively. Filled flat arrowheads indicate the first band of teeth, empty flat arrowheads indicate the second band of teeth, and filled indented arrowheads indicate the third band of teeth. Scale bars in μm

Mouth antero-ventral. Buccal apparatus of the *Macrobiotus* type (Fig. 35a), with the ventral lamina and ten peribuccal lamellae (Fig. 35a–b). The oral cavity armature was composed of three bands of teeth, from which only the second and third bands were always clearly visible under LCM (Fig. 34b–c), whereas the first band was only visible under SEM (Fig. 35a–b). The first band of teeth is composed of numerous small teeth visible as globular cones with SEM (Fig. 35a–b), arranged in several rows, and situated anteriorly in the oral cavity, just behind the bases of the peribuccal lamellae. The second band of teeth is situated between the ring fold and the third band of teeth and comprises 3–4 rows of teeth visible with LCM as granules (Fig. 34b–c) and with SEM as cones (Fig. 35a–b) but larger than those in the first band. The posterior row of teeth within the second band seems to comprise larger teeth than the previous anterior rows (Fig. 34b–c). The teeth of the third band are located within the posterior portion of the oral cavity, between the second band of teeth and the buccal tube opening (Figs. 34b–c, 35a–b). The third band of teeth is divided into the dorsal and ventral portions. Under both LCM and SEM, the dorsal teeth are seen as three distinct transverse ridges, whereas the ventral teeth appear as two separate lateral transverse ridges, between which one large tooth (sometimes circular in LCM) is visible (Figs. 34b–c, 35a–b). In SEM, only teeth of the dorsal portion in the third band have clearly indented margins (Fig. 35a). Pharyngeal bulb spherical, with triangular apophyses, two rod-shaped macroplacoids (2<1) and a microplacoid positioned close to them (i.e., the distance between the second macroplacoid and the microplacoid is shorter than the microplacoid length; Fig. 34d–e). The first macroplacoid is anteriorly narrowed and constricted in the middle, whereas the second has a subterminal constriction (Fig. 34d–e).

**Fig. 36:**
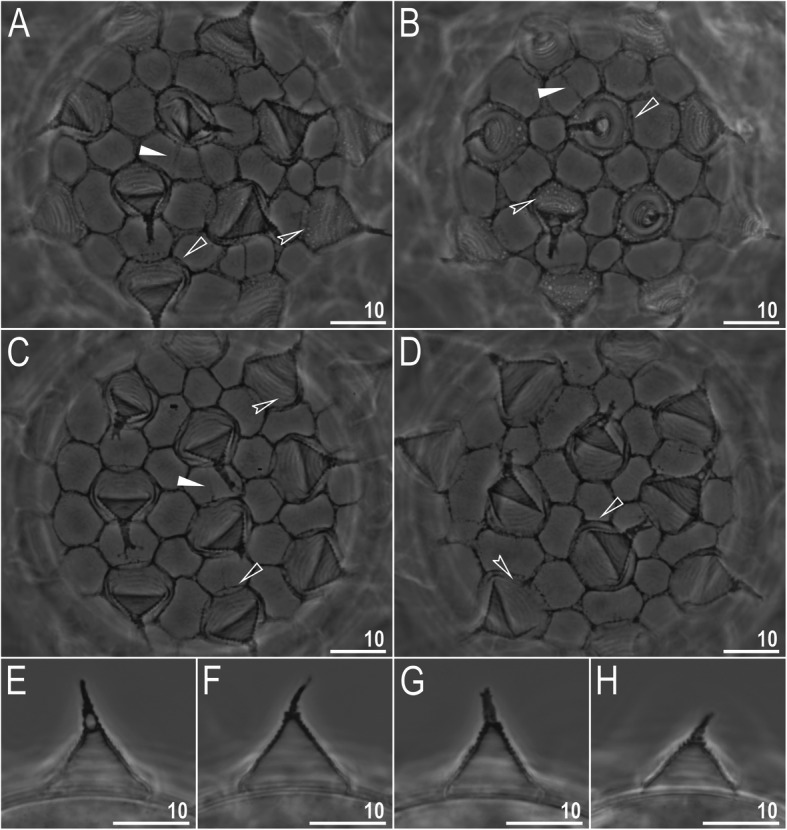
*Macrobiotus margoae* **sp. nov.** from the USA (US.057) – eggs seen with LCM: **a–d** – surface under ×1000 magnification of four different eggs; **e–h** – midsections of four different egg processes. Filled flat arrowheads indicate thickenings perpendicular to the process base that divide the areola in the middle, empty indented arrowheads indicate bubbles within egg process walls, and empty flat arrowheads indicate irregular collar around process bases. Scale bars in μm

**Fig. 37:**
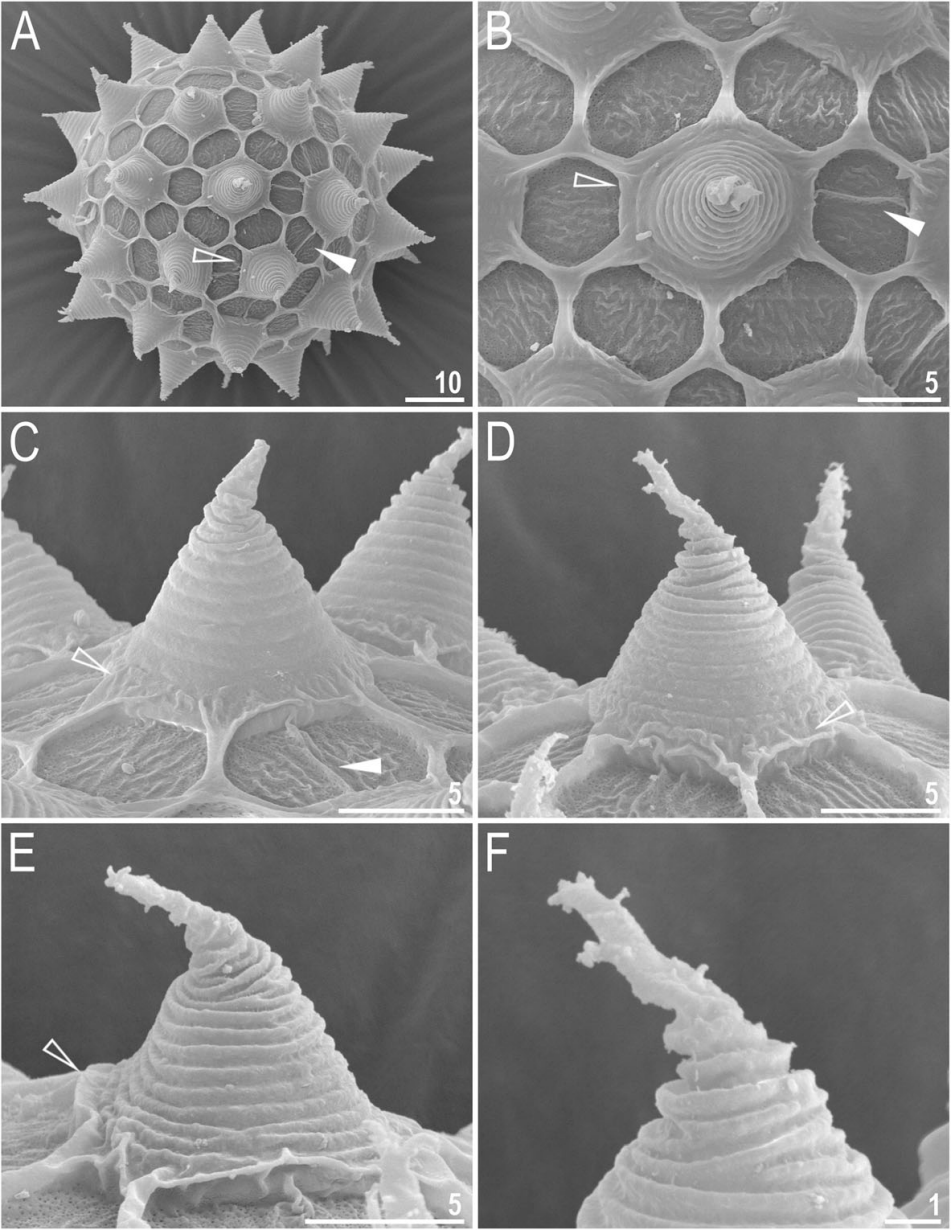
*Macrobiotus margoae* **sp. nov.** from the USA (US.057) – eggs seen with SEM: **a** – entire view of the egg; **b–f** – details of the egg surface between processes, areolation and egg processes. Filled flat arrowheads indicate thickenings perpendicular to the process base that divide the areola in the middle, empty flat arrowheads indicate irregular collar around process bases. Scale bars in μm

***Eggs*** *(measurements and statistics in* Table 13*):* Laid freely, white, spherical with conical processes surrounded by one row of areolae (Figs. 36a–h, 37a–f). In SEM, multiple rings of tight annulation on the entire process surface were visible (Fig. 37a–f) (annulation not visible in LCM because it was obscured by the eminent labyrinthine layer). The upper parts of the processes are smooth and not covered by granulation (Fig. 37c–f). The labyrinthine layer between the process walls was present and usually visible only as small circular bubbles scattered randomly on the process (Fig. 36a–b). Rarely, these bubbles are almost not visible (Fig. 36c–d). The upper part of the process is often elongated into short flexible apices that can be occasionally broken and that can sometimes be bifurcated and have bubble-like structures (Figs. 36e–h, 37c–f). The base of the processes extends into the six (only sometimes five) arms that form areolae rims (Figs. 36a–d,37a–e). When this flattened part is properly developed, the process base can be seen as hexagonal or pentagonal from the top view (Figs. 36a–d, 37a–b). Each process is surrounded by six (only sometimes five) hexagonal areolae (Figs. 37a–d, a–e), which are occasionally falsely subdivided in the middle into two areolae by a narrow thickening perpendicular to the process base (Figs. 36a–c, 37a–c). Areolae rims (walls) are usually thin and flat (Fig. 36a–d, 37a–e), with the labyrinthine layer inside the rims sometimes visible as bubbles under LCM (Fig. 36a–d). Areolae rims delimit the areolae at the bases of processes, which forms an irregular collar around process bases (Figs. 36a–d, 37a–d) and makes the process bases penta- or hexagonal in the top view (Figs. 36a–d, 37a–b). The areola surface has wrinkles that are faintly visible under LCM (Fig. 36a–d) but clearly visible under SEM (Fig. 37a–e). Micropores are present within the areolae, but they are distributed only around the areolae rims and are usually absent in the central part of the areola (Fig. 37b–e).

**Table 13:** Measurements [in μm] of selected morphological structures of the eggs of *Macrobiotus margoae* **sp. nov.** from the USA (US.057) mounted in Hoyer’s medium (N–number of eggs/structures measured, RANGE refers to the smallest and largest structures among all measured specimens; SD–standard deviation)

CHARACTER	N	RANGE	MEAN	SD
Egg bare diameter	28	63.3 – 76.3	69.3	3.4
Egg full diameter	28	72.9 – 96.9	86.5	7.2
Process height	84	8.4 – 15.6	12.3	2.0
Process base width	84	8.2 – 16.5	12.5	1.5
Process base/height ratio	84	80% – 137%	104%	15%
Interprocess distance	84	4.1 – 8.3	6.1	1.2
Number of processes on the egg circumference	28	10 – 14	12.1	1.0

### Reproduction

**Fig. 38:**
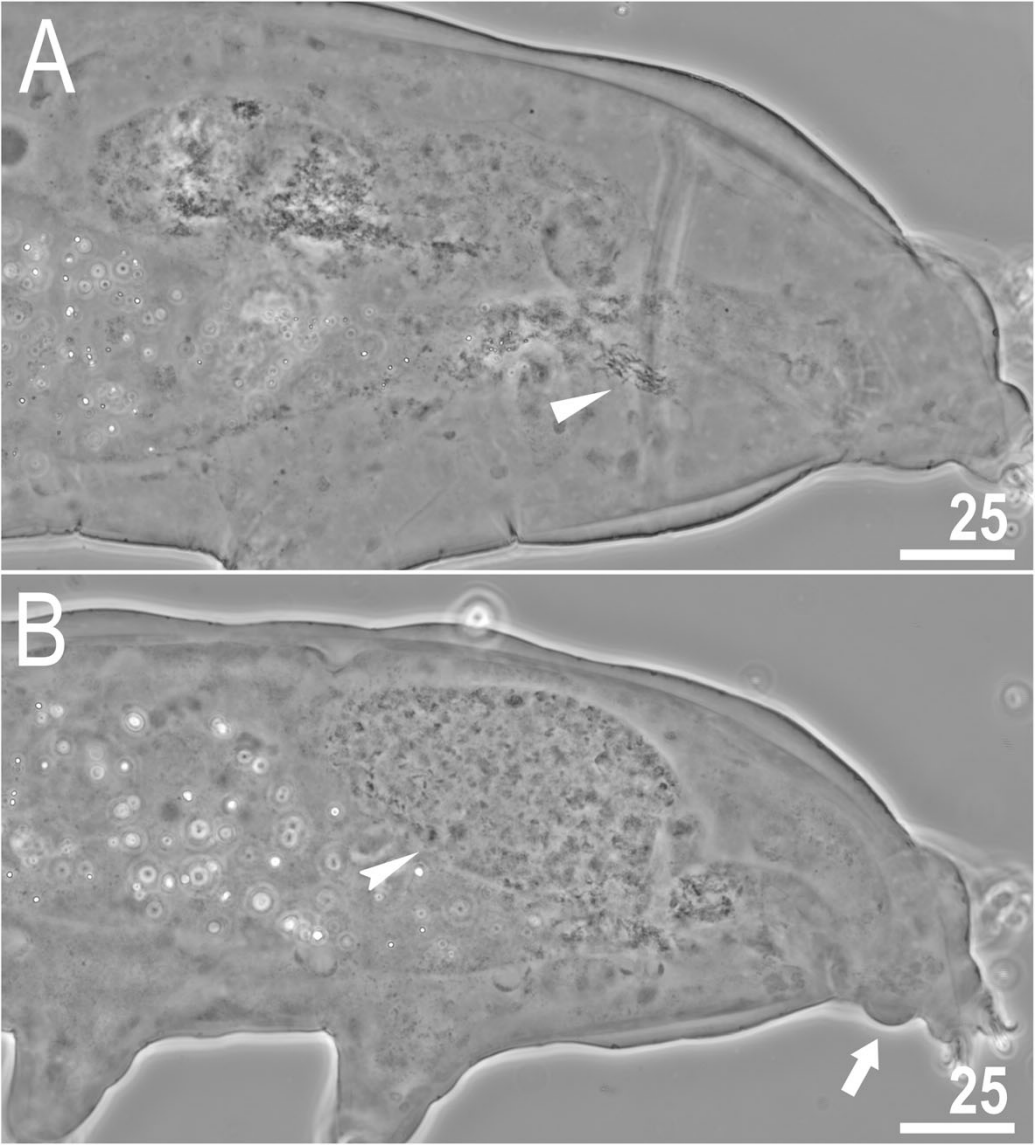
*Macrobiotus margoae* **sp. nov.** from the USA (US.057) – reproduction (LCM): **a** – spermatheca (seminal vesicle) filled with spermatozoa and visible in females freshly mounted in Hoyer’s medium; **b** – testis filled with sperm visible in a male freshly mounted in Hoyer’s medium. The flat arrowhead indicates the female spermathecae, the indented arrowhead indicates the testis, and the arrow indicates gibbosity on the IV leg. Scale bars in μm

The species is dioecious. Spermathecae in females as well as testes in males have been found to be filled with spermatozoa, clearly visible under PCM up to 24 hours after mounting in Hoyer’s medium (Fig. 38a–b). The species exhibits secondary sexual dimorphism in the form of clearly visible lateral gibbosities on hind legs in males (Fig. 38b).

### DNA sequences and intraspecific genetic distances

- **18S rRNA** sequences (GenBank: MT809072–3), 987 bp long; 1 haplotype was found.
- **28S rRNA** sequences (GenBank: MT809084–5), 715 bp long; 1 haplotype was found.
- **ITS-2** sequences (GenBank: MT809097–9), 361 bp long; 2 haplotypes were found, separated by a p-distance of 0.8%.
- **COI** sequences (GenBank: MT807927–8), 630 bp long; 1 haplotype was found.

### Phenotypic differential diagnosis

By having the processes surrounded by 5–6 areolae, it resembles four other species of the *Macrobiotus pallarii* complex out of which two are newly described in this study. By the morphology of the animals and eggs, this species can be differentiated from the following:

- ***Macrobiotus pallarii***: by a weakly developed oral cavity armature, with the first band of teeth not visible under LCM (the oral cavity armature is well developed, and the first band of teeth is visible under LCM in *M. pallarii*), by dentate lunulae IV (lunulae are faintly crenulate in *M. pallarii*), by the absence of two lateral patches of dense granulation between legs III and IV (dense granulation patches between legs III and IV are present in *M. pallarii*; see Fig. 1), by the absence of a sparse dorsal granulation between legs III and IV (sparse granulation is present in *M. pallarii*; see Fig. 1), by a lower placoid row *pt* value (*51.1–60.6* in *M. margoae* **sp. nov.** vs. *61.3–75.6* in *M. pallarii*), by the presence of small circular bubbles scattered randomly within the egg process walls (meshes are present within the entire process walls in *M. pallarii*), and by the absence of granulation on the egg process tips (granulation is present in *M. pallarii*; character visible only under SEM).
- ***Macrobiotus pseudopallarii*** **sp. nov.**: by a weakly developed oral cavity armature, with the first band of teeth not visible under LM (the oral cavity armature is well developed, and the first band of teeth is visible under LM in *M. pseudopallarii* **sp. nov.**), by dentate lunulae IV (lunulae are gently dentate in *M. pseudopallarii* **sp. nov.**), by the absence of two lateral patches of dense granulation between legs III and IV (the dense granulation patches between legs III and IV are present in *M. pseudopallarii* **sp. nov.**; see Fig. 1), by the absence of sparse dorsal granulation between legs III and IV (the sparse granulation is present in *M. pseudopallarii* **sp. nov.**; see Fig. 1), by a lower placoid row *pt* value (*51.1–60.6* in *M. margoae* **sp. nov.** vs. *65.0–75.1* in *M. pseudopallarii* **sp. nov.**), by the presence of small circular bubbles scattered randomly within the process walls (meshes are present within the walls of the entire process in *M. pseudopallarii* **sp. nov.**), and by and the absence of granulation on the egg processes tips (granulation is present in *M. pseudopallarii* **sp. nov.**; character visible only under SEM).
- ***Macrobiotus ripperi*** **sp. nov.**: by a weakly developed oral cavity armature, with the first band of teeth not visible under LCM (the oral cavity armature is well developed, and the first band of teeth is visible under LCM in *M. ripperi* **sp. nov.**) and by the absence of sparse dorsal granulation between legs III and IV (sparse granulation is present in *M. ripperi* **sp. nov.**; see Fig. 1).
- ***Macrobiotus caymanensis***: by the presence of granulation visible under LCM on all legs (granulation is not visible in *M. caymanensis*) and by dentate lunulae IV (lunulae are smooth in *M. caymanensis*).

### Genotypic differential diagnosis

Interspecific genetic p-distances between *M. margoae* **sp. nov.** and other species of the *M. pallarii* complex are as follows:

- **18S rRNA**: 1.2–1.4% (1.2% on average), with the most similar being *Macrobiotus pallarii* from Italy (MT809069–71) and *Macrobiotus ripperi* **sp. nov.** from Poland and Finland (MT809074–6), and the least similar being *Macrobiotus pseudopallarii* **sp. nov.** from Montenegro (MT809067–8),
- **28S rRNA**: 2.1–2.7% (2.3% on average), with the most similar being *Macrobiotus ripperi* **sp. nov.** from Poland and Finland (MT809086–9), and the least similar being *Macrobiotus pallarii* from Italy (MT809081–3).
- **ITS-2**: 6.1–8.6% (7.8% on average), with the most similar (to H2) being *Macrobiotus pallarii* from Italy (MT809094–6) and the least similar (to H2) being *Macrobiotus ripperi* **sp. nov.** H1 from Poland and Finland (MT809100–3).
- **COI**: 21.1–22.7% (21.4% on average), with the most similar being *Macrobiotus pallarii* from Italy (MT807924–6) and the least similar being *Macrobiotus ripperi* **sp. nov.** H3 from Finland (MT807930–2).

## Dichotomous diagnostic key to species of the *Macrobiotus pallarii* complex

To facilitate species identification, we provide a dichotomous key to the valid species of the *M. pallarii* complex. When differentiating these species, we stress the need for analyzing the largest possible number of individuals and eggs, as some characters, such as egg process reticulation, show intraspecific variability (see the Taxonomic Account above), and some more conservative characters, such as dorso-caudal granulation, may not always be easily detected due to suboptimal orientation of specimens on slides or/and microscope quality.

- **1** Egg processes surrounded by 5–6 areolae..............2
- Egg processes surrounded by 11–12 areolae ***M. ragonesei*** Binda et al., 2001 [26]
- **2(1)** Granulation on all legs present.................3
- Granulation on all legs absent or not visible with LCM ***M. caymanensis*** Meyer, 2011 [28]
- **3(2)** Two lateral patches of dense granulation between legs III and IV present................................4
- Two lateral patches of dense granulation between legs III and IV absent..................................5
- **4(3)** Lunules IV faintly crenulate, sparse granulation connecting the dense granulation patches between legs III and IV not extending posteriorly.......................***M. pallarii*** Maucci, 1954 [22]
- Lunules IV dentate, sparse granulation connecting the dense granulation patches between legs III and IV extending posteriorly to the granulation on legs IV........***M. pseudopallarii*** **sp. nov.**
- **5(3)** Sparse dorsal granulation between legs III and IV present............................***M. ripperi*** **sp. nov.**
- Sparse dorsal granulation between legs III and IV absent.........................***M. margoae*** **sp. nov.**

## Discussion

*Macrobiotus pallarii* was described from Italy in 1954, and it has been reported from Europe and North America [25, 89, 90]. We recovered a population of this species from the type locality (Silvana Mansio, Cosenza, Italy) in an environment matching the original species description (moss on rock on the ground in a sparse forest [22]). The assignment of population IT.337 to *M. pallarii* was confirmed by a comparison with the type series from the Maucci collection (Natural History Museum of Verona). The analysis of the topotypes of *M. pallarii*, with four distinct populations (that would have been attributed to the same species prior to this study), showed that four pseudocryptic species can be distinguished from morphological, morphometric, and genetic viewpoints. Due to the presence of these morphological and genetic differences, we described them as three new species (see the Taxonomic Account above).

### Species delineation in the *Macrobiotus pallarii* complex

Congruent with the results of species delimitation of different populations of the *Paramacrobiotus areolatus* group by [7], the COI showed higher divergence than ITS-2, and a strikingly similar pattern was found for a pair of closely related *P. areolatus* group populations (IT.048 and PT.006 in [7]) with contrasting species delimitation results based on the two analyzed markers (distinct species according to COI, same species according to ITS-2). Similar differences in species delimitation based on COI and ITS-2 markers were obtained for some species in the genus *Milnesium*, although in contrast to the present study, the precedence was given to ITS-2, as no phenotypic differences between units delineated with COI were detected [12, 91]. Thus, it seems that there is no simple rule to decide which genetic marker is better suited for species delineation in tardigrades. Rather, both phenotypic and genetic traits should be used in tandem in taxonomic analyses.

### Species diversity in the *Macrobiotus pallarii* complex

**Fig. 39:**
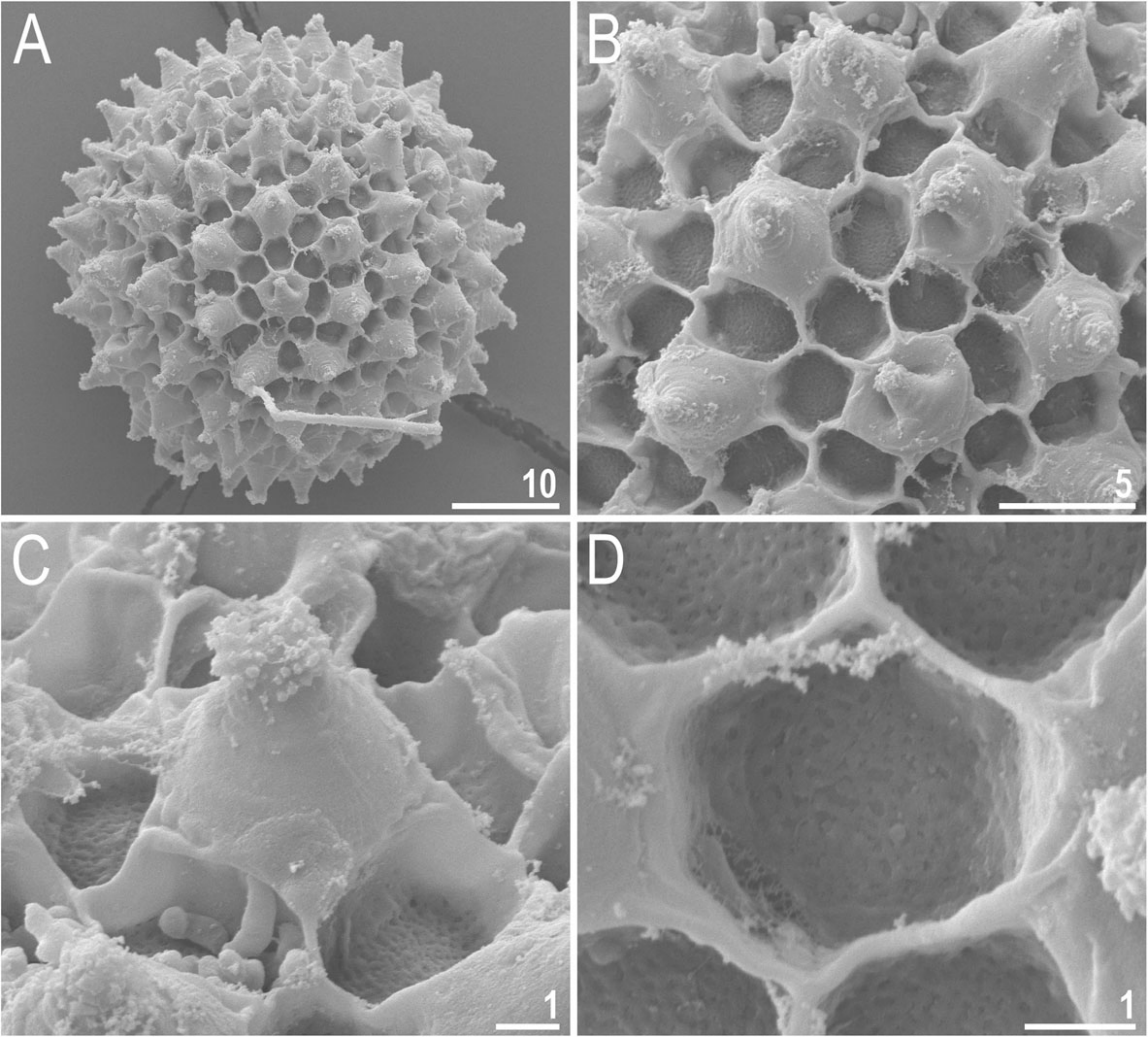
*Macrobiotus* aff. *polyopus* species from Papua New Guinea – eggs seen with SEM: **a** – entire egg; **b–d** – details of the egg surface between the processes, areolation and egg processes. Scale bars in μm

*Macrobiotus pallarii, M. ragonesei*, *M. caymanensis*, and the three new species form the *M. pallarii* complex (defined by [19] as having cone-shaped egg processes separated by one row of areolation). The identification of pseudocryptic species in the *M. pallarii* complex reopens the question about the identity of *M. aviglianae*, which has been synonymised with *M. pallarii* by [24]. The analysis of the *M. aviglianae*-type material (slides CT1954, CT1955, CT1966, CT4051 and CT4485 of the Maucci collection) showed that it was not in good condition. Thus, it is not possible to verify whether *M. aviglianae* does not differ from *M. pallarii* in any subtle traits, such as in the pattern of dorso-caudal granulation or granulation on the tips of egg processes. Moreover, it is also not possible to test whether *M. aviglianae* represents an independent phyletic lineage or if it falls within the intraspecific genetic variability of *M. pallarii*. Thus, only the discovery of a new population of *M. aviglianae* in its type locality will allow for a proper test of its synonymy with *M. pallarii*. The morphometric characters of the *M. caymanensis* type overlap with those of the newly described *M. margoae* **sp. nov.**, with which it also shares a similar appearance of the egg process wall. Thus, it is probable that these two species are phylogenetically close; however, it will be possible to test this hypothesis only after genetic data for *M. caymanensis* are obtained. For *M. ragonesei*, detailed morphometric data are not available, precluding its inclusion in the PCA, but based on morphology alone, it is clear that the species deviates from all other known members of the complex by a much higher number of areolae around each egg process (11–12 *vs*. 5–6 in the remaining species). *Macrobiotus ragonesei* was collected only once in the type locality in the Democratic Republic of Congo (Central Africa); however, it is possible that the population collected and identified as *Macrobiotus* cf. *pallarii* by [92] from Swaziland (South Africa) represents *M. ragonesei* or a related species because eggs from the Congolese population resembled those of *M. ragonesei* (“processes are rounded rather than tapering, and the number of areolae surrounding them is ten to twelve rather than eight or nine” [92]). Another species, *Macrobiotus insularis* Pilato, 2006 [93], known only from the Andaman Islands in the Indian Ocean, is currently assigned to the *Macrobiotus polyopus* group [93]. However, because of its similarity with the species of the *M. pallarii* complex, it would not be surprising if after a reanalysis of the type material, the species is moved to the latter complex. Additionally, *M. caymanensis* was originally described as a member of the *M. polyopus* group, which suggests that both groups have not been sufficiently differentiated and doubts about the assignment to one or the other group are possible. In our opinion, the *M. polyopus* group can be differentiated from the *M. pallarii* complex by the morphology of egg processes and areola walls (rims) (Fig. 39a). Specifically, process base width and interprocess distances were similar in the *M. polyopus* group (Fig. 39b), whereas process bases were much wider than the distance between neighboring processes in the *M. pallarii* complex (Fig. 37b). Moreover, areolae are always delimited by high and thin walls in the *M. polyopus* group (usually nearly half the process height, Fig. 39c–d), whereas areolae walls are low and often thick, with the labyrinthine layer visible in LCM, in the *M. pallarii* complex (Fig. 37c–d). Thus, currently, there are six confirmed species in the *M. pallarii* complex, with further potential members: *M. insularis* (pending the verification of the type material) and *M. aviglianae* (pending an examination of a topotypic population). However, the number of species constituting the complex is likely to be much higher, as has been shown in other eutardigrade groups when tools of integrative taxonomy and large-scale sampling have been employed, for example, in the genus *Milnesium* [94], the genera *Astatumen* and *Platicrista* [95], the *Ramazzottius oberhaeuseri* complex [38], the genus *Macrobiotus* (e.g., [19]), the *Paramacrobiotus richtersi* and *areolatus* complexes ([5, 7], respectively), or in the genus *Richtersius* [6, 13].

### Biogeography of the *Macrobiotus pallarii* complex

Species of the *Macrobiotus pallarii* complex are not commonly found, but the complex has a wide, possibly global distribution. Although the majority of records are concentrated in Europe (Finland, Greece, Hungary, Italy, Montenegro, Norway, Poland, Republic of Bulgaria, Russian Federation, Turkey, Yugoslavia [25, 82, 96–98]), there are also reports from Asia (North Korea, Russian Federation [25]), North America (United States, Cayman Islands [28, 86–88]), and Africa (Democratic Republic of the Congo, Swaziland [27, 92]). In light of our findings, all these records except the type localities should be considered dubious and be designated as species of the *Macrobiotus pallarii* complex, i.e., as *Macrobiotus* aff. *pallarii* spp., keeping open the possibility they could represent yet undescribed new species.

### The importance of disentangling pseudocryptic species complexes

Thanks to the use of integrative taxonomy, pseudocryptic and cryptic tardigrade species complexes are being detected with increasing frequency (chronologically [6, 7, 11, 13, 14, 19, 38, 99–103]). Other than for purely taxonomic purposes, acknowledging and discerning such entities may significantly impact studies concerning biodiversity, biogeography, ecology or physiology. Specifically, biodiversity studies and taxonomic checklists need precise taxonomic identifications to be comparable across different geographical areas. However, as noted, e.g., by [82], numerous identifications cannot be confirmed due to the presence of (pseudo) cryptic species complexes. Furthermore, biogeographical studies supported by molecular data on tardigrades are still scarce. In one of them, Gąsiorek et al. [104] showed evidence suggesting allopatric speciation in a complex of tardigrades (*Echiniscus virginicus* complex) that are almost indistinguishable morphologically but genetically divergent, underlining the need for genetic verification of faunistic records to draw sound biogeographic conclusions. Regarding ecology, for example, Faurby et al. [105] found that *Echiniscoides* species have different geographical distributions and thus also various ecological requirements despite being very closely related. The *M. pallarii* complex also seems to be ecologically and physiologically diversified, as *M. ripperi* **sp. nov.** performs very well in laboratory culture (D.S. and M.V. personal observation), whereas the other examined species (*M. pallarii*, *M. pseudopallarii* **sp. nov.**, *M. margoae* **sp. nov.**), while able to reproduce in culture, they did not thrive. Whether these differences in reproductive success under uniform laboratory conditions are a result of natural variability in the physiology or different levels of alignment of the ecology of these species and laboratory conditions (*i.e.,* the similarity of laboratory conditions, such as temperature, fodder type or photoperiod, to the conditions to which the species is adapted in nature) has yet to be tested. Nevertheless, in both cases, species identity, despite the striking morphological similarity, is an important variable in the equation and cannot be ignored.

Differences in ecological and physiological traits in species that exhibit very little morphological differentiation have also been demonstrated for cryptic species complexes in other taxa (e.g., bryozoans, diatoms and butterflies [106–108]). The presence of cryptic species that show physiological and life history divergence can also influence laboratory studies when the adopted model species belong to one of those complexes. For example, studies on the model chordate sea squirt *Ciona intestinalis* (Linnaeus, 1767 )[109] assumed that all worldwide natural populations belong to a single species [110]. However, it was later demonstrated that cryptic species that produce sterile hybrid offspring are present [111]. Acknowledgment of the presence of cryptic species in *C. intestinalis* turned out to be essential for experimental design and data management [110], as, for example, new laboratory strains could not be obtained by crossing the cryptic species. There are also similar examples concerning tardigrades. Specifically, individuals attributed to *Paramacrobiotus richtersi* were used by [112] to test the effects of a lower earth orbit environment on tardigrade physiology and reproduction. However, it was later revealed that individuals of that population belong to a different cryptic species of the *P. richtersi* complex, *Paramacrobiotus spatialis* Guidetti et al., 2019 [5]. As species in the *P. richtersi* complex show different geographic distributions [5] and are likely to have different environmental requirements and tolerances, their misidentification (due to the recognition of the species complex years later) could affect the replicability of the 2011 study if the experiment was replicated with another species of the complex identified as “*P. richtersi*”. Similarly, more tardigrade species used in experimental studies were found to belong to cryptic (or pseudocryptic) species complexes and misidentified for the nominal species of the complex, for example *Hypsibius exemplaris* was misidentified as *H. dujardini* [113], *Milnesium inceptum* was misidentified as *M. tardigradum* [114] and *Richtersius* sp. 4 was misidentified as *R. coronifer* [6]. Importantly, while discrepancies stemming from results obtained with different (pseudo) cryptic species could be a problem when not recognized, such species may also provide a useful tool to investigate tardigrade speciation and how isolation between such morphologically similar species is maintained by using the tools of experimental taxonomy, such as interpopulation crosses [7] and mate choice assays, as well as omics techniques.

## Conclusions

Integrative taxonomy revealed that multiple pseudocryptic species, each probably with a limited geographic distribution, are present under what was once considered a single cosmopolitan species, “*Macrobiotus pallarii*”. Species of the *Macrobiotus pallarii* complex can be differentiated principally by dorsal granulation and egg ornamentation but also statistically by morphometric traits. Other than for purely taxonomic reasons, disentangling tardigrade cryptic and pseudocryptic species complexes is fundamental for the proper interpretation of biogeographical and ecological studies and the replicability of experimental works. Due to the rising popularity of tardigrade species as laboratory models (e.g., [17, 115–124]), the presence of cryptic or pseudocryptic complexes must be taken into account to avoid incongruent results stemming from taxonomic misidentifications.

## Supplementary Information

**Additional file 1: SM.01.** Saturation plots for COI and ITS markers.

**Additional file 2: SM.02.** MrBayes analysis input file with the alignment.

**Additional file 3: SM.03.** The p-distances between species of the *Macrobiotus pallarii* complex.

**Additional file 4: SM.04a.** R data for morphometric analysis.

**Additional file 5: SM.04b.** R script for morphometric analysis.

**Additional file 6: SM.05.** Raw morphometric data for *Macrobiotus pallarii* Maucci, 1954 from the topotypic population (IT.337).

**Additional file 7: SM.06.** Raw morphometric data for *Macrobiotus pseudopallarii* sp. nov. from Montenegro (ME.007, type population).

**Additional file 8: SM.07.** Raw morphometric data for *Macrobiotus ripperi* sp. nov. from Poland (PL.015, type population).

**Additional file 9: SM.08.** Raw morphometric data for *Macrobiotus ripperi* sp. nov. from Finland (FI.066, additional population).

**Additional file 10: SM.09.** Raw morphometric data for *Macrobiotus margoae* sp. nov. from USA (US.057, type population).

**Additional file 11: SM.10.** Results of PCA randomization tests.

## Data Availability

All data generated or analyzed during this study are included in this published article [and its supplementary information files]. Genetic data are deposited in GenBank, NCBI.
