# Supplementary material for: Integrative taxonomy resolves species identities within the Macrobiotus pallarii complex (Eutardigrada: Macrobiotidae)
Source: Zoological Lett. 2021 May 27;7:9. doi: 10.1186/s40851-021-00176-w (PMC8162020; doi:10.1186/s40851-021-00176-w)
Supplement: Supplementary file 1 — Additional file 1: SM.01. Saturation plots for COI and ITS markers. [file 40851_2021_176_MOESM1_ESM.pdf]

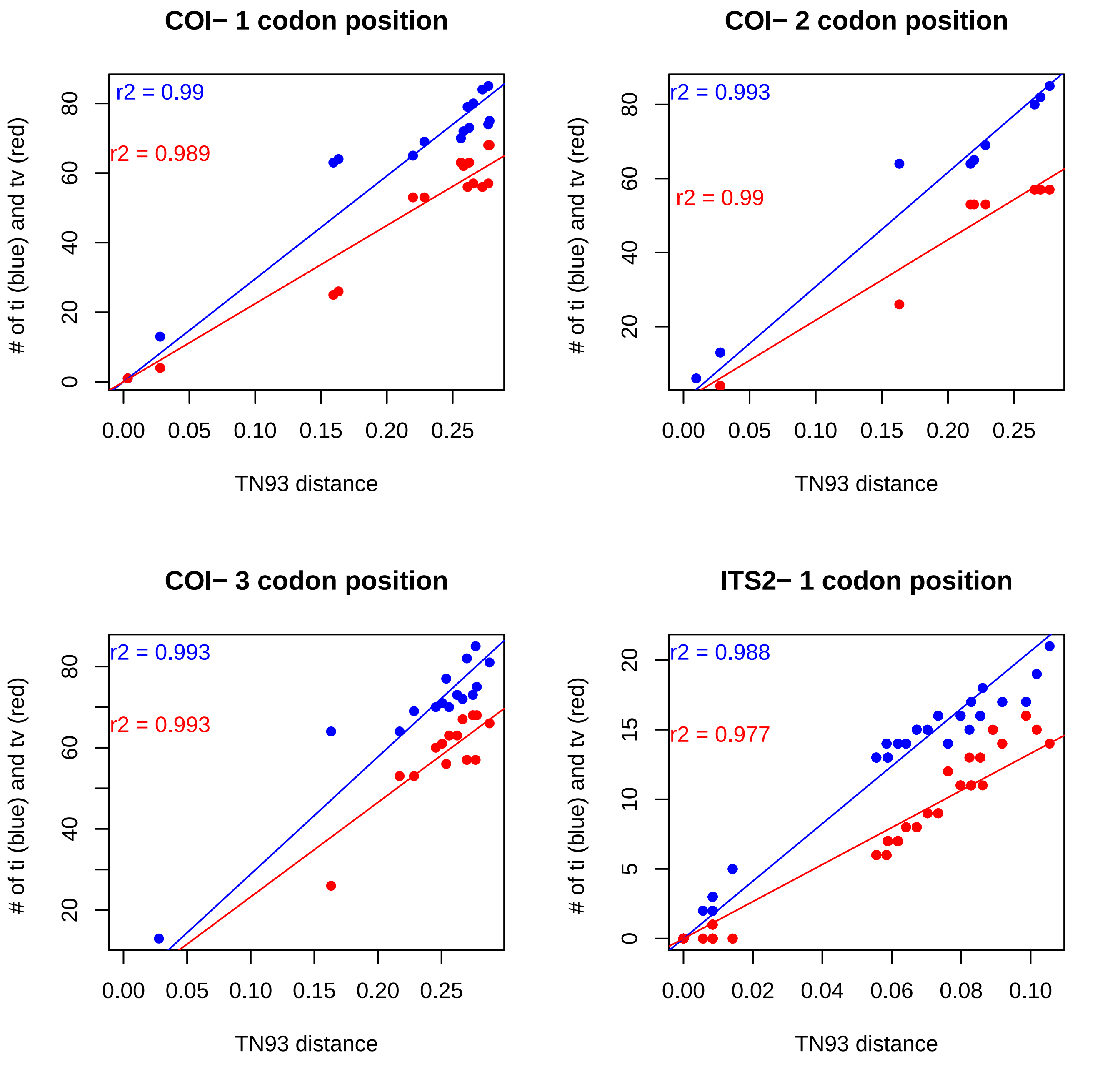
COI− 1 codon position
r2 = 0.99
r2 = 0.989
# of ti (blue) and tv (red)
0
20
40
60
80
0.00
0.05
0.10
0.15
0.20
0.25
TN93 distance
COI− 2 codon position
r2 = 0.993
r2 = 0.99
# of ti (blue) and tv (red)
20
40
60
80
0.00
0.05
0.10
0.15
0.20
0.25
TN93 distance
COI− 3 codon position
r2 = 0.993
r2 = 0.993
# of ti (blue) and tv (red)
20
40
60
80
0.00
0.05
0.10
0.15
0.20
0.25
TN93 distance
ITS2− 1 codon position
r2 = 0.988
r2 = 0.977
# of ti (blue) and tv (red)
0
5
10
15
20
0.00
0.02
0.04
0.06
0.08
0.10
TN93 distance

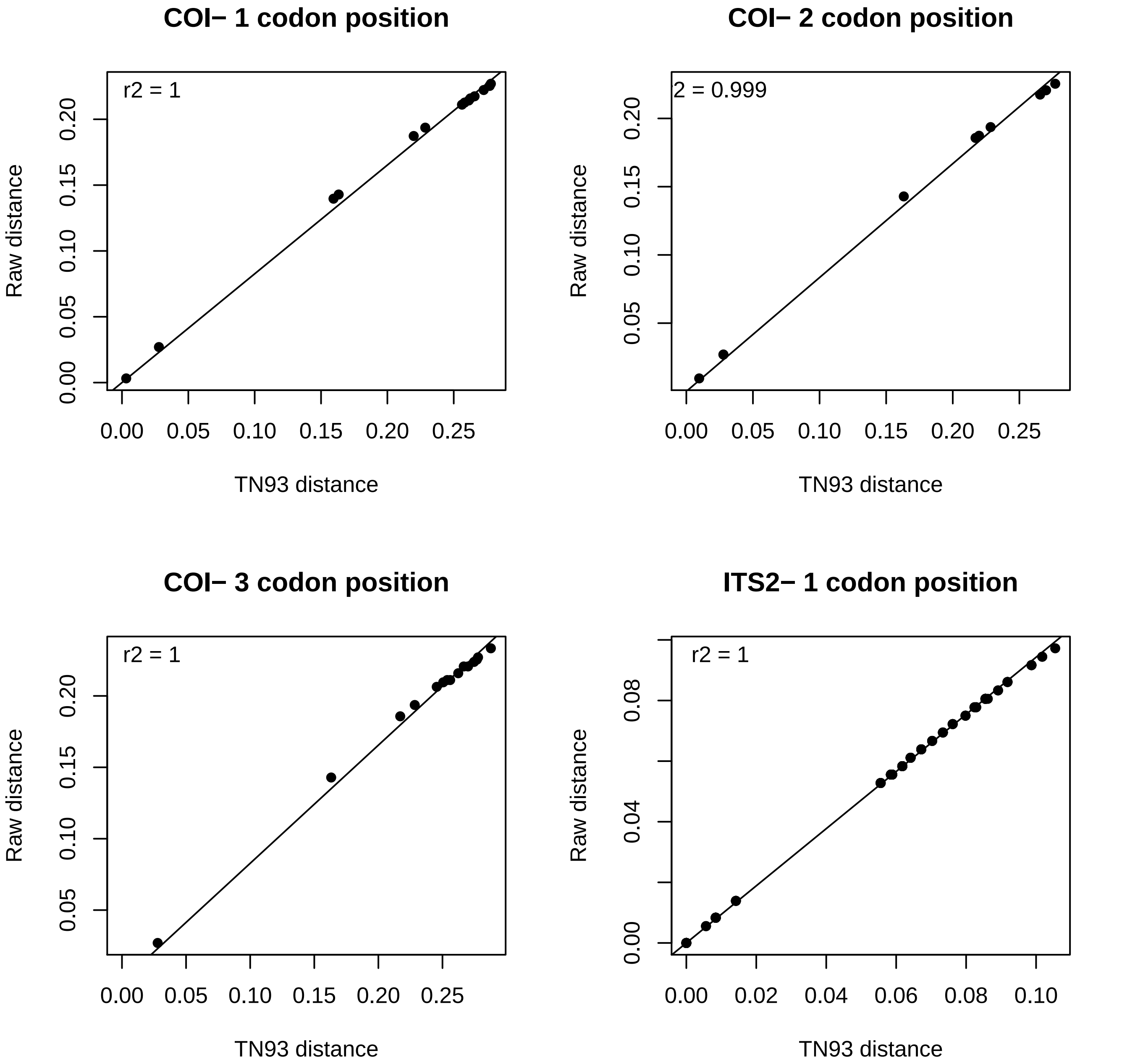
COI− 1 codon position
r2 = 1
Raw distance
0.00
0.05
0.10
0.15
0.20
0.25
TN93 distance
COI− 2 codon position
2 = 0.999
Raw distance
TN93 distance
COI− 3 codon position
r2 = 1
Raw distance
TN93 distance
ITS2− 1 codon position
r2 = 1
Raw distance
0.02
0.04
0.06
0.08
TN93 distance
