# Supplementary material for: Integrative taxonomy resolves species identities within the Macrobiotus pallarii complex (Eutardigrada: Macrobiotidae)
Source: Zoological Lett. 2021 May 27;7:9. doi: 10.1186/s40851-021-00176-w (PMC8162020; doi:10.1186/s40851-021-00176-w)

Integrative taxonomy resolves species identities within the Macrobiotus pallarii complex (Eutardigrada: Macrobiotidae)

DANIEL STEC[a], MATTEO VECCHI[b], MAGDALENA DUDZIAK[a], PAUL J. BARTELS[c], SARA CALHIM[b] & ŁUKASZ MICHALCZYK[a]

[a]Institute of Zoology and Biomedical Research, Jagiellonian University, Gronostajowa 9, 30-387 Kraków, Poland
[b]Department of Biological and Environmental Science, University of Jyvaskyla, PO Box 35, FI-40014, Jyvaskyla, Finland
[c]Department of Biology, Warren Wilson College, Asheville, NC 28815, USA
Correspondence: daniel_stec@interia.eu, matteo.vecchi15@gmail.com

SM.11. Results of PCA randomisation tests.

Figure SM11.1. Observed ψ statistic of the PCA on animals morphometrics dataset and null distribution of the same parameter for 1000 PCAs on randomly permuted animals morphometrics datasets.

Figure SM11.2. Observed ϕ statistic of the PCA on animals morphometrics dataset and null distribution of the same parameter for 1000 PCAs on randomly permuted animals morphometrics datasets.

Figure SM11.3. Observed explained variances of the PCA on animals morphometrics dataset and null distribution of the same parameters for 1000 PCAs on randomly permuted animals morphometrics datasets.

Figure SM11.4. Observed ψ statistic of the PCA on eggs morphometrics dataset and null distribution of the same parameter for 1000 PCAs on randomly permuted eggs morphometrics datasets.

Figure SM11.5. Observed ϕ statistic of the PCA on eggs morphometrics dataset and null distribution of the same parameter for 1000 PCAs on randomly permuted eggs morphometrics datasets.

Figure SM11.6. Observed explained variances of the PCA on eggs morphometrics dataset and null distribution of the same parameters for 1000 PCAs on randomly permuted eggs morphometrics datasets.

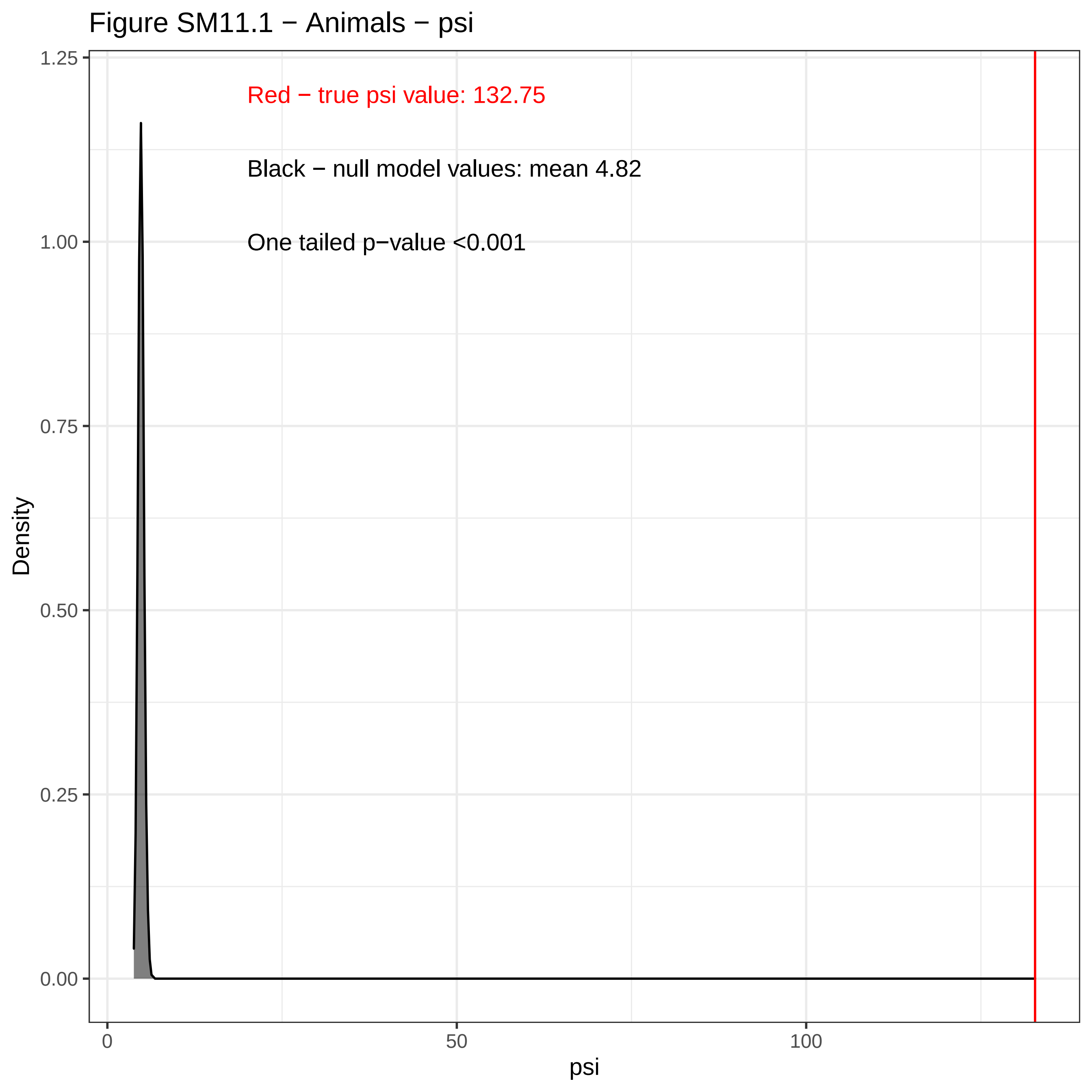

Figure SM11.1 − Animals − psi
Red − true psi value: 132.75
Black − null model values: mean 4.82
One tailed p−value <0.001
Density
psi
0.00
0.25
0.50
0.75
1.00
1.25
0
50
100

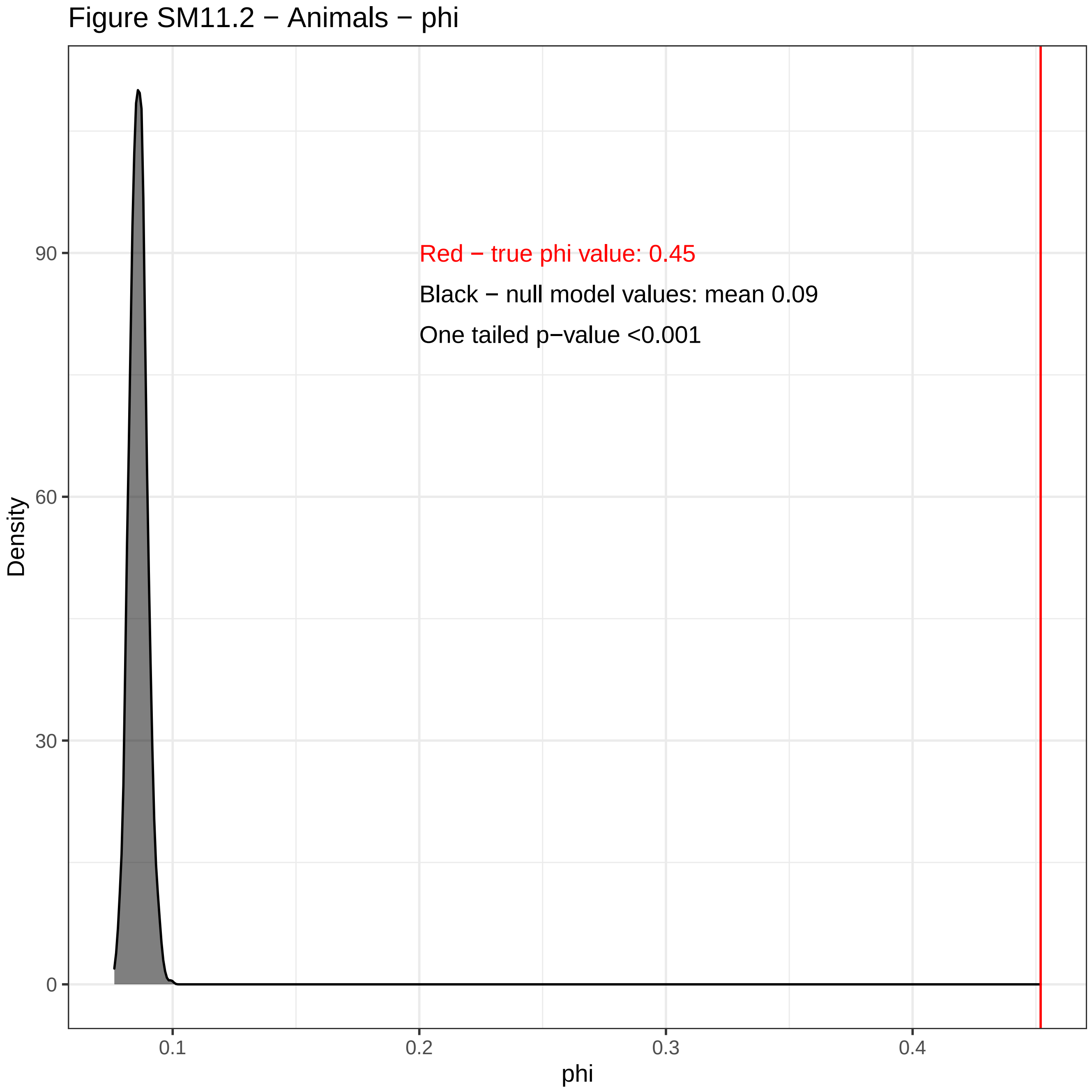
Figure SM11.2 − Animals − phi
Red − true phi value: 0.45
Black − null model values: mean 0.09
One tailed p−value <0.001
Density
phi
0
30
60
90
0.1
0.2
0.3
0.4

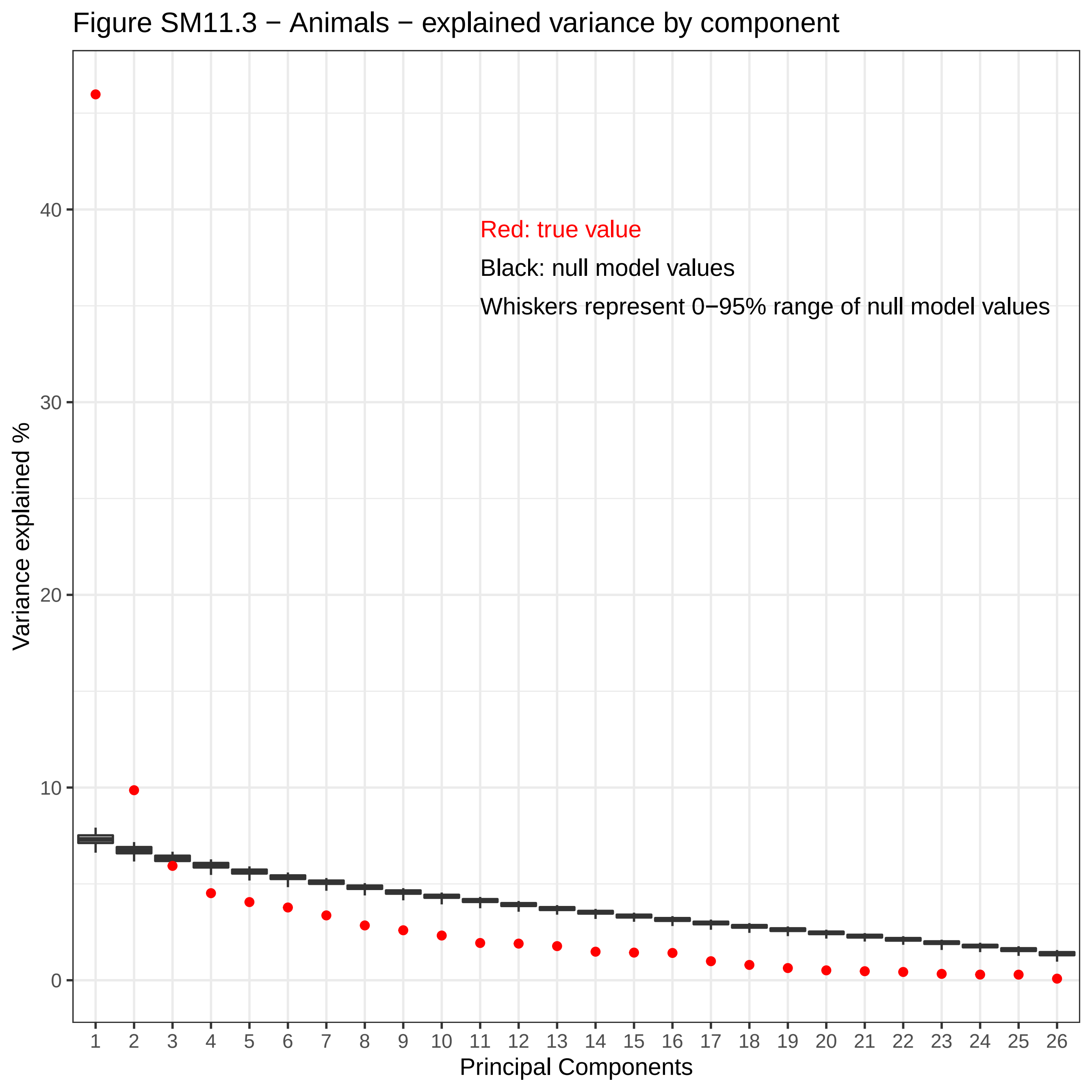
Figure SM11.3 − Animals − explained variance by component
Red: true value
Black: null model values
Whiskers represent 0−95% range of null model values
Variance explained %
0
10
20
30
40
1
2
3
4
5
6
7
8
9
10
11
12
13
14
15
16
17
18
19
20
21
22
23
24
25
26
Principal Components

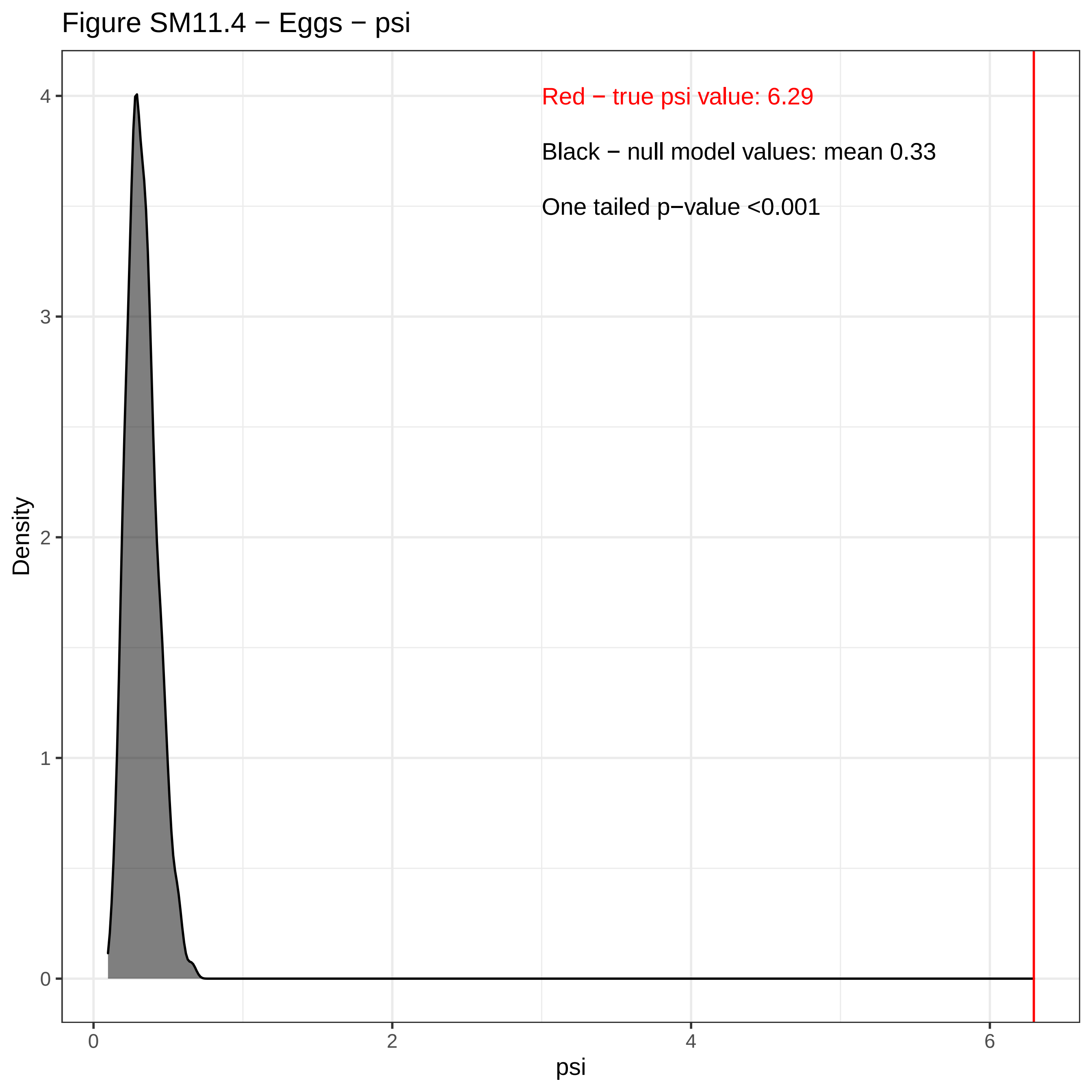
Figure SM11.4 − Eggs − psi
Red − true psi value: 6.29
Black − null model values: mean 0.33
One tailed p−value <0.001
Density
psi
0
1
2
3
4
0
2
4
6

Figure SM11.5 − Eggs − phi

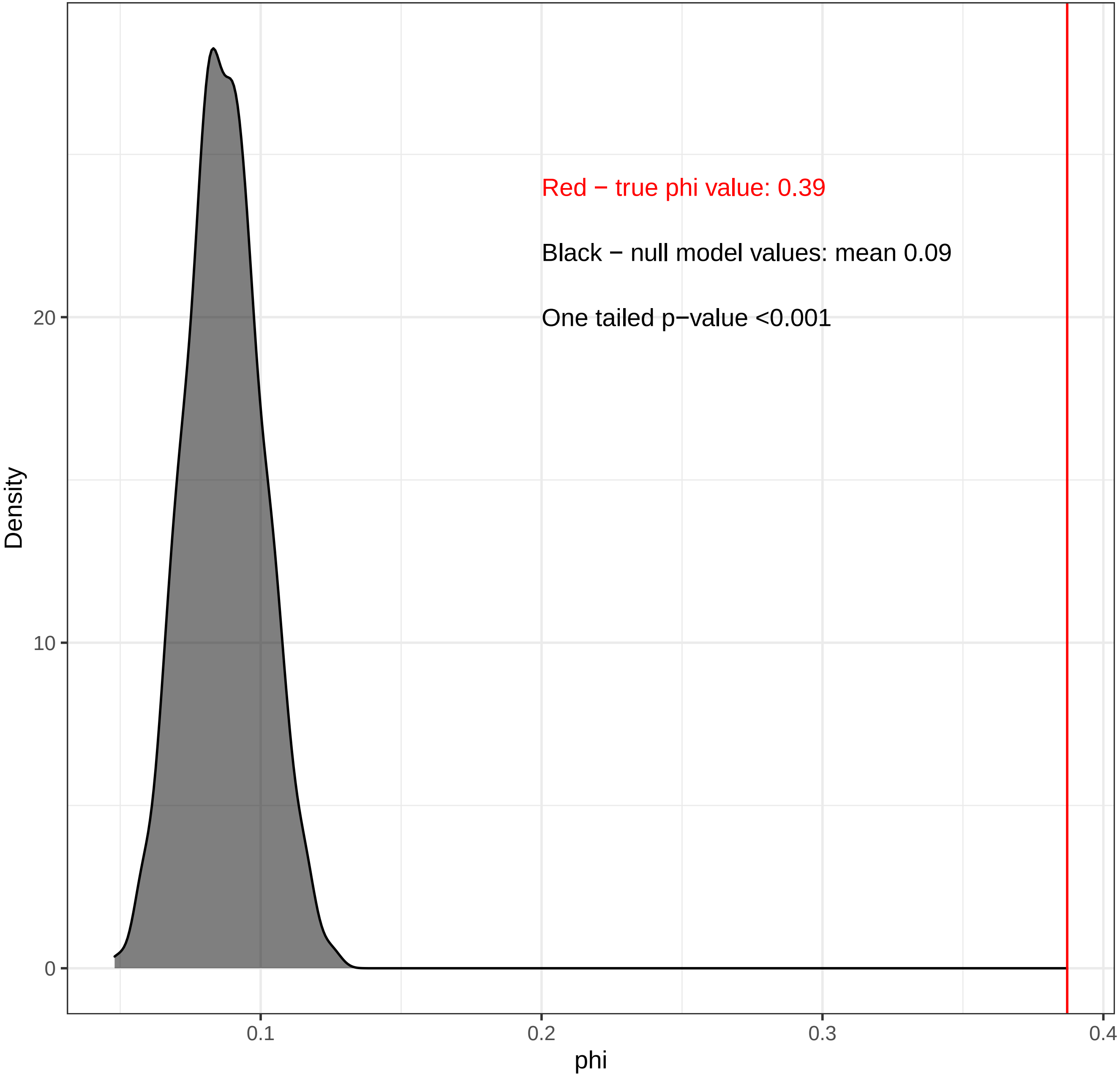

Figure SM11.6 − Eggs − explained variance by component

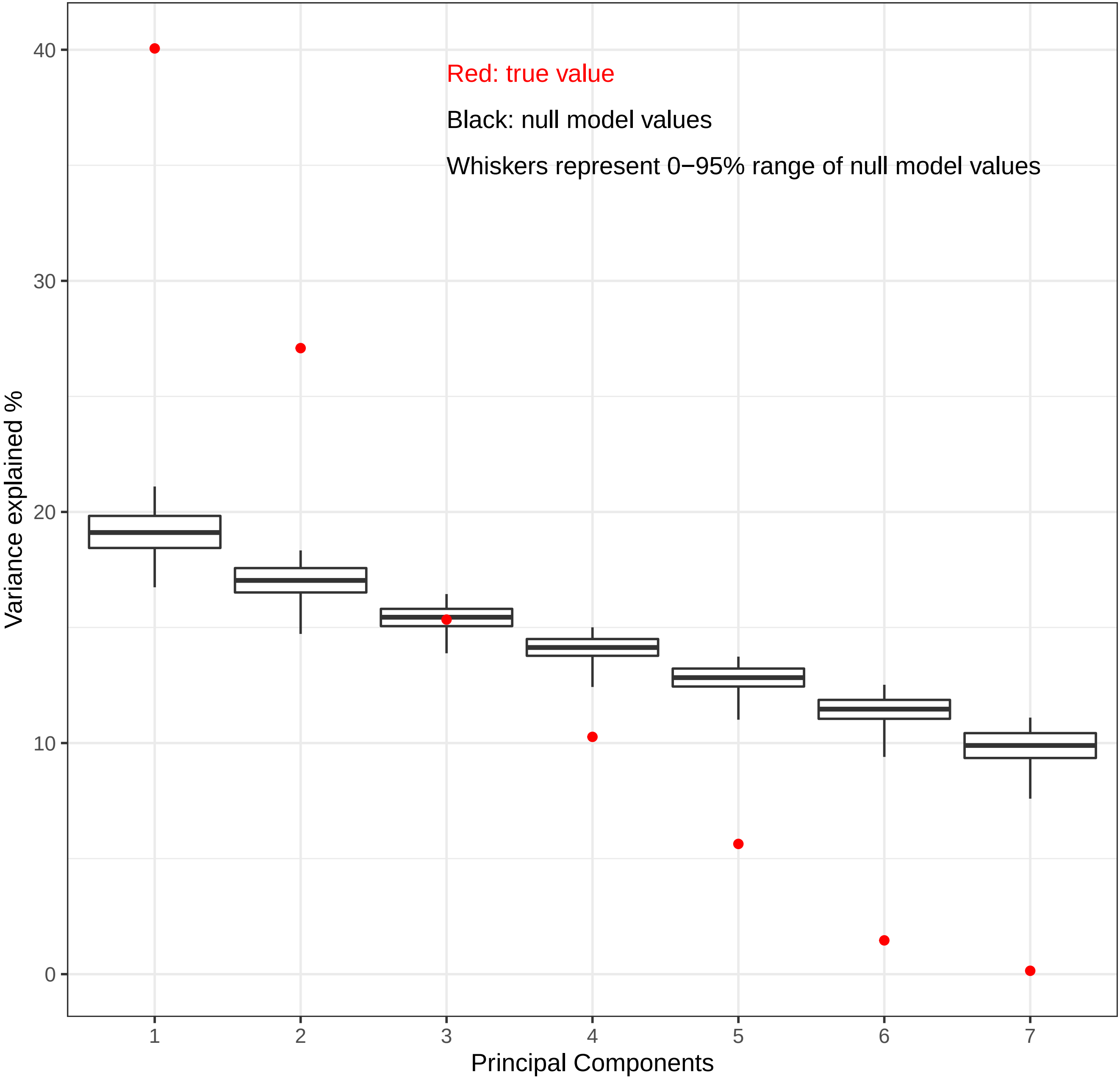

Supplement: Supplementary file 11 — Additional file 11: SM.10. Results of PCA randomization tests. [file 40851_2021_176_MOESM11_ESM.pdf]
